# Computational Validation of Multi‐Epitope mRNA Vaccine Targeting *Streptococcus anginosus* Surface Protein (TMPC) as an Effective Alternative Treatment to Reduce Gastric Cancer

**DOI:** 10.1002/mbo3.70230

**Published:** 2026-02-10

**Authors:** Fei Zhu, Yuying Luo, Ziyou Zhou, Rongliu Qin, Shiyang Ma, Yizhong Xu, Jie Chen, Pinhua Pan

**Affiliations:** ^1^ Department of Respiratory Medicine, National Key Clinical Specialty, Branch of National Clinical Research Center for Respiratory Disease, Xiangya Hospital Central South University Changsha Hunan China; ^2^ National Clinical Research Center for Geriatric Disease Xiangya Hospital Changsha Hunan China; ^3^ FuRong Laboratory Changsha Hunan China

**Keywords:** gastric cancer, molecular modeling, mRNA vaccine, multi‐epitopes, *Streptococcus anginosus*

## Abstract

*Streptococcus anginosus* is a Gram‐positive coccus that can increase gastric cancer risk through interaction with the TMPC‐ANXA2‐MAPK axis in gastric epithelial cells. There is currently no commercially available vaccine, and prolonged antibiotic treatment may increase drug resistance. We developed a *Treponema pallidum* membrane protein C (TMPC)‐based multi‐epitope vaccine targeting nine TMPC‐positive streptococcal species dominated by *S. anginosus*. B‐cell and T‐cell epitopes were chosen based on their binding affinity, antigenicity, immunogenicity, and safety, with adjuvants and linker sequences improving construct stability and immune response. Immune simulations predicted robust humoral and cellular responses, such as cytokine production and memory cell activation. Molecular docking and molecular dynamics analysis further confirmed stable interactions between the vaccine construct and key immune receptors (HLA‐A*02:01, HLA‐DRB1*01:01, TLR2, and TLR4). The antigen was further modified as a messenger RNA vaccine to enhance cytotoxic T‐cell induction; however, animal research is needed to confirm its immunogenicity and protective effectiveness.

## Graphical abstract

Design process for messenger RNA vaccines. Candidate proteins are initially screened using immunoinformatics, after which selected epitopes are assembled into a multi‐epitope vaccine. The vaccine construct was evaluated through molecular docking, molecular dynamics simulations, and in silico immune simulations, followed by population coverage analysis and messenger RNA vaccine design.

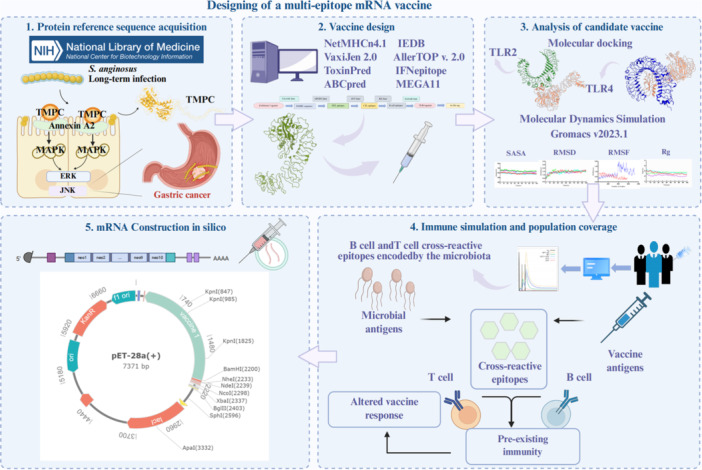

## 1 Introduction

One major factor in cancer‐related death is gastric carcinomas, the fifth most common cancer globally (Sung et al. 2021). Gastric cancer develops through a complex, multistage, and complicated process. *Helicobacter pylori* infection is classified as a Group I carcinogen and has historically been identified as a major risk factor for gastric adenocarcinoma (Wang et al. 2014). However, approximately 1%–3% of *H. pylori* infections result in stomach cancer (Herrera and Parsonnet 2009). It is hypothesized that other microorganisms may also contribute to the development of stomach cancer (Marshall and Warren 1984). As early as 2018, a study discovered that the stomach mucosa of Chinese gastric cancer patients who tested negative for *H. pylori* was enriched with five different oral pathogens, including *Streptococcus anginosus* (Coker et al. 2018). Additionally, new studies have shown that *S. anginosus* is a key factor in the development of stomach cancer (Fu et al. 2024).

*S. anginosus*, a Gram‐positive coccus, exhibits significant tolerance to acidic environments (pH 3.0–5.0), a trait that may enhance its survival within the gastric mucosa (Sasaki et al. 2018). Clinical studies indicate systemic *S. anginosus* infections in patients with esophageal, gastric, and oral cancers, implying a possible association between prolonged colonization and tumorigenesis (Fu et al. 2024; Lin et al. 2008; Sasaki et al. 2005). Recent metagenomic analyses reveal that *S. anginosus* leverages its surface protein *Treponema pallidum* membrane protein C (TMPC) to interact with Annexin A2 (ANXA2) receptor on gastric epithelial cells, promoting its colonization and invasion and activating mitogen‐activated protein kinase (MAPK) signaling in gastric cancer (Fu et al. 2024). The MAPK signaling pathway controls important cellular functions, like, differentiation, proliferation, and programmed cell death. Its abnormal activation can lead to epithelial hyperplasia and may play a role in the onset of gastric cancer (Fang and Richardson 2005). Research indicates that, within the gastric cancer‐enriched microbiota, besides *S. anginosus*, eight additional *Streptococcus* species (*Streptococcus intermedius*, *Streptococcus constellatus*, *Streptococcus gordonii*, *Streptococcus oralis*, *Streptococcus sanguinis*, *Streptococcus parasanguinis*, *Streptococcus koreensis*, and *Streptococcus gwangjuense*) exhibiting high TMPC expression were identified, suggesting that these strains may also contribute to disease onset and progression via TMPC‐related pathways (Fu et al. 2024). All of these species belong to the *S. anginosus* group (SAG), formerly known as the *Streptococcus milleri* group (Clarridge et al. 1999; Gobbetti and Calasso 2014; Lim et al. 2019; Park et al. 2019; Whiley and Beighton 1991). Although these microorganisms are normally part of the symbiotic microbiota of the human oral cavity and gastrointestinal tract, they can cause severe, invasive purulent infections (Junckerstorff et al. 2014). From 2010 to 2017, the incidence of SAG infections steadily rose, peaking at 3.7 cases per 100,000 individuals (Laupland et al. 2018). Penicillin G and cephalosporins are the preferred medications for treating SAG (Giuliano et al. 2012); however, rising resistance to macrolides and tetracyclines presents clinical challenges (Clarridge et al. 2001). Consequently, the development of a vaccine against this bacterial infection can effectively control the pathogen and avert gastric cancer resulting from prolonged colonization.

To explore a novel strategy for preventing gastric cancer, we designed a multi‐epitope messenger RNA (mRNA) vaccine targeting the TMPC protein of nine TMPC‐positive *Streptococcus* species, predominantly *S. anginosus*. Following vaccine construction, we first evaluated its basic physicochemical properties and overall structural organization. We subsequently conducted a comprehensive analysis of its interactions with immune receptors, structural stability, and potential to elicit robust immunological responses. These evaluations employed a suite of computational approaches, including molecular docking, molecular dynamics (MD) simulations, and various immunoinformatics analyses. This combination of methodologies constitutes an efficient pathway that can facilitate vaccine design and bolster the advancement of novel preventive techniques for SAG‐associated gastric cancer.

## 2 Methods and Materials

### 2.1 Protein Reference Sequence Acquisition

The reference protein sequences of the TMPC from nine TMPC‐positive streptococci adhering to the gastric cancer were collected from the NCBI database: *S. anginosus* (VED97779.1), *S. constellatus* (WP_19845817.1), *S. gordonii* (VUX13697.1), *S. gwangjuense* (WP_001036137.1), *S. intermedius* (RSJ24290.1), *S. koreensis* (WP_270308737.1), *S. oralis* (WP_268710078.1), *S. parasanguinis* (WP_254729306.1), and *S. sanguinis* (WP_124764763.1).

### 2.2 Epitope Identification

#### 2.2.1 Forecasting Cytotoxic T Lymphocyte (CTL) Epitopes

The NetMHCpan4.1 server comprehensively evaluates the binding affinity of predicted epitopes to major histocompatibility complex (MHC) class I molecules (Reynisson et al. 2020). To maximize vaccine coverage across global populations, 12 widely prevalent HLA supertypes were selected (HLA‐A*01:01, HLA‐A*02:01, HLA‐A*03:01, HLA‐A*24:02, HLA‐A*26:01, HLA‐B*07:02, HLA‐B*08:01, HLA‐B*27:05, HLA‐B*39:01, HLA‐B*40:01, and HLA‐B*58:01). For each TMPC protein, all possible 9‐mer peptides were initially generated, yielding several hundred candidate cytotoxic T lymphocyte (CTL) epitopes. The CTL epitopes that strongly bind these MHC‐I alleles were retained, with percentile ranks below 0.5%. TepiTool was applied as a validation tool for NetMHCpan predictions and to predict epitope–allele interactions across multiple species, with epitopes selected at a percentile rank < 0.5% (Paul et al. 2016). Antigenicity was evaluated using VaxiJen v2.0, and epitopes with scores > 0.4 were selected (Doytchinova and Flower 2007). To ensure safety, allergenicity and toxicity were assessed using AllerTOP v2.0 and ToxinPred, respectively, and any epitopes predicted to be allergenic or toxic were excluded (Gupta et al. 2013; Sharma et al. 2021). Finally, the immune epitope database (IEDB) immunogenicity tool was used to evaluate each epitope's ability to stimulate immune responses, and only epitopes with positive immunogenicity scores (> 0) were retained for inclusion in the final multiepitope vaccine construct.

#### 2.2.2 Forecasting Helper T Lymphocyte (HTL) Epitopes

To identify HTL epitopes, a set of 27 high‐coverage Human Leukocyte Antigen‐DR (HLA‐DR) alleles was selected as the alleles using the MHC‐II binding prediction tool on the IEDB server platform (Jensen et al. 2018). All potential HTL peptide candidates were initially generated, and epitopes with strong binding affinity (percentile rank < 1%) to at least one HLA‐DR allele were retained as preliminary candidates. TepiTool was used to predict all corresponding allele interactions. These candidates were further screened for antigenicity (VaxiJen v2.0, score > 0.4), allergenicity (AllerTOP v2.0), and toxicity (ToxinPred), and epitopes predicted to be allergenic or toxic were excluded. Subsequently, the IFNepitope server was employed to evaluate the ability of HTL epitopes to induce Interferon‐γ (IFN‐γ) production by CD4⁺ T cells, and only IFN‐γ‐inducing epitopes were retained for vaccine construction (Dhanda et al. 2013).

#### 2.2.3 Forecasting Linear B‐Cell Epitopes

Linear B‐cell epitopes were anticipated employing the ABCpred service with a threshold of 0.51 (EL‐Manzalawy et al. 2008). Subsequently, the antigenicity, allergenicity, and toxicity of these epitopes were evaluated via the VaxiJen 2.0, AllerTOP v2.0, and ToxinPred servers. To enhance confidence in B‐cell epitope selection, only epitopes with high ABCpred prediction scores (> 0.8) and strong antigenicity (VaxiJen score > 1.0), while also being non‐allergenic and non‐toxic, were selected as final linear B‐lymphocyte (LBL) candidates for inclusion in the multi‐epitope vaccine construct.

#### 2.2.4 Conservative Analysis and Multiple Sequence Alignment

TMPC protein sequences were collected from 24 *Streptococcus* species that are potentially pathogenic to humans. Epitope conservancy was evaluated with the IEDB Epitope Conservancy Analysis tool. Multiple sequence alignment was conducted with the Molecular Evolutionary Genetics Analysis Version 11 (MEGA11) software, which was used to generate the phylogenetic tree based on the Neighbor‐Joining algorithm (Tamura et al. 2021). The optimal tree was determined by calculating the bootstrap value after 1000 iterations. Additionally, the ChiPlot server was employed to construct phylogenetic trees and illustrate the distribution of epitopes among various strains using a heatmap (Xie et al. 2023). To mitigate the danger of epitope‐induced autoimmunity and cross‐reactivity, we assessed the homology of the projected epitopes with human proteins, along with proteins from 79 prevalent intestinal probiotic species and the human oral microbiota. The human proteome (Taxon ID: 9606) was downloaded from the NCBI database and used to construct a local database. Homology between the epitopes and human proteins was analyzed using the BLASTp program via Diamond v2.1.12 software, with an *E* < 0.0001. Protein sequences of 79 common intestinal probiotic species collected from published articles were also downloaded from NCBI, and sequence alignment was performed using the BLASTp program via Diamond with the *E* < 0.0001. Subsequently, the proteomes of the human oral microbiota were obtained from the Human Oral Microbiome Database v4.1 and aligned with the epitopes using BLASTp under the same *E* value.

### 2.3 Docking of T‐Cell Epitopes and MHC Molecules

Understanding the intricate interactions between MHC molecules and T‐cell epitopes is fundamental for unraveling immune responses (Wieczorek et al. 2017). Initially, three‐dimensional (3D) structures of selected T‐cell epitopes were generated through structural predictions using the PEP‐FOLD3 service (Lamiable et al. 2016). The Research Collaboratory for Structural Bioinformatics (RCSB) Protein Data Bank (PDB) website served as a resource for obtaining 3D structures of MHC class I and II molecules (Burley et al. 2022); if unavailable, modeling was performed using the SWISS‐MODEL server (Guex and Peitsch 1997). Validation of model accuracy was conducted through assessments with ProSA‐web, ERRAT, and PROCHECK (Wiederstein and Sippl 2007). Subsequently, the Swiss‐PDB Viewer software was employed to optimize the energy of receptors and ligands (Kaplan 2001), while the ClusPro 2.0 server facilitated the molecular docking of T‐cell epitopes with MHC molecules (Kozakov et al. 2017). The results of the docking simulations were further scrutinized using LigPlot+ v.2.2 for comprehensive analysis (Laskowski and Swindells 2011). PyMOL 2.5 software can visualize docking structures, and the PROtein binDIng enerGY prediction (PRODIGY) tool is used to calculate the binding affinity between molecules (Seeliger and de Groot 2010; Xue et al. 2016).

### 2.4 Vaccine Development

TLR2 and TLR4, expressed in immune cells such as monocytes and macrophages, play key roles in recognizing pathogens and initiating immune responses (Mukherjee et al. 2016). Adjuvants targeting these receptors can enhance the immunogenicity of vaccines. Thymopentin (TP5), a TLR2 agonist, can activate TLR2‐mediated signaling to stimulate immune response (Wei et al. 2021). Human beta‐defensin 3 (hBD‐3), a small cationic antimicrobial peptide released by epithelial cells, exhibits both antibacterial and immunomodulatory activities, contributing to innate and adaptive immunity (Dhople et al. 2006). RS09 (APPHALS), a synthetic TLR4 agonist, promotes maturation of antigen‐presenting cells when incorporated into antigens (Ito et al. 2017).

To enhance immune activation, hBD‐3 or TP5, were incorporated at the N terminus, while a TLR4 agonist (RS09) was added at the C terminus. Rigid Glu‐Ala‐Ala‐Ala‐Lys (EAAAK) linkers joined adjuvants to the vaccine, ensuring structural stability and correct folding (X. Chen et al. 2013). Flexible linkers were used to join the epitopes: Ala‐Ala‐Tyr (AAY) for CTL to facilitate MHC‐I processing, Gly‐Pro‐Gly‐Pro‐Gly (GPGPG) for HTL to prevent masking and aid MHC‐II presentation, and KK for B‐cell epitopes to enhance exposure and antibody maturation (Ahmad et al. 2024; Ma et al. 2025). Additionally, a Pan HLA‐DR‐Associated Epitope (PADRE) pan‐DR HTL epitope was included to activate broad CD4⁺ T‐cell help and amplify CTL and B‐cell responses (Franke et al. 1999). A 6xHis tag was appended to the C terminus to facilitate purification via Ni‐NTA affinity chromatography (Carter and Outten 2021). The SOLpro server was utilized to assess the potential solubility of the vaccine upon overexpression in *Escherichia coli* (Magnan et al. 2009).

### 2.5 Prediction of Antigenicity, Allergenicity, Toxicity, and Physicochemical Properties of the Vaccine

The antigenicity of the vaccine design was assessed utilizing VaxiJen 2. AllerTOP 2.0 assessed allergenicity, whereas ToxinPred 2.0 predicted toxicity. The ProtParam server was used to predict the vaccination sequence, concentrating on its chemical and physical properties (Garg et al. 2016).

### 2.6 Secondary and 3D Structure Predictions, 3D Structure Refinement, and Validation

To analyze the secondary structure of the vaccine, the PSIPRED 4.0 server was utilized for its expertise in accurately determining the topological and helical configurations within transmembrane structures (Buchan and Jones 2019). The tertiary structure of the multi‐epitope vaccine was predicted using advanced deep‐learning methods by employing Chai 1 and AlphaFold (Chai Discovery Team et al. 2024; Varadi et al. 2022). Following the modeling process, MD simulations were performed. The model was placed in a simulation box and subjected to energy minimization. Equilibration was achieved over a period of 20 or 30 ns, resulting in an optimized model. The primary structures were then refined using the GalaxyRefine server, which improves structure quality by altering side chains and overall relaxation (Heo et al. 2013). The assessment was based on the MolProbity parameter, with a lower value indicating higher model quality (V. B. Chen et al. 2010). Validation of the modified models included the study of PROCHECK‐generated Ramachandran plots, ERRAT scores, and ProSA web‐generated *Z* scores (Pilarczyk‐Zurek et al. 2022; Wiederstein and Sippl 2007). Tertiary protein structures have the potential to create novel conformational B‐cell epitopes. The ElliPro server was employed to forecast discontinuous B‐cell epitopes within protein structures (Ponomarenko et al. 2008).

### 2.7 Docking of Vaccine‐Toll‐Like Receptors (TLRs)

The 3D structures of TLRs (TLR2 and TLR4) and HLA molecules (HLA‐A*02:01 and HLA‐DRB1*01:01) were obtained from the RCSB PDB. Protein–protein docking between the multi‐epitope vaccine and TLR2 (PDB ID: 2Z81), TLR4 (PDB ID: 2Z63), HLA‐A*02:01 (PDB ID: 4U6Y), or HLA‐DRB1*01:01 (PDB ID: 1AQD) was performed using the ClusPro 2.0 server, which generates and clusters multiple docking conformations using a fast Fourier transform algorithm. Top‐ranked models based on cluster size and energy were selected for refinement using HADDOCK 2.4, which applies restrained MD and energy minimization in explicit water to yield stable atomic‐level 3D complexes (Ambrosetti et al. 2023). Interaction residues were analyzed using PDBsum (Laskowski et al. 2018), and the vaccine–receptor complexes were visualized with ChimeraX v1.7 (Pettersen et al. 2021). Two experimentally validated TLR‐binding proteins (PepO—modeled using AlphaFold, Agarwal et al. 2013; Shu et al. 2020; Hemolysin—PDB ID:5AOD, Malley et al. 2003) were docked with the TLRs and analyzed using PDBsum to compare the interactions between the vaccine and TLR receptors.

### 2.8 MD Simulation

To evaluate structural stability and dynamic behavior of the vaccine–receptor complexes (HLA‐A*02:01, HLA‐DRB1*01:01, TLR2, and TLR4), MD simulations were conducted using GROMACS v2023.3 (Tan et al. 2022). Systems were prepared with the Amberff99sb‐aldn force field, solvated in SPC/E water, and neutralized with 0.15 M NaCl in dodecahedral boxes with a 1.0‐nm minimum distance from protein to box edge (Lindorff‐Larsen et al. 2010). First, a 400 ps canonical ensemble (constant number of particles, volume, and temperature) (canonical) ensemble was run to stabilize the system temperature at 310 K. This was followed by a 400 ps isothermal‐isobaric ensemble (constant number of particles, pressure, and temperature) (NPT) (isothermal‐isobaric) ensemble to equilibrate the pressure to 1 bar. During both phases, position restraints were applied to the heavy atoms of the protein. The stability and convergence of key thermodynamic properties were carefully monitored to confirm that the system reached equilibrium. Finally, production MD simulations were performed for 100 ns under the NPT ensemble (310 K and 1 bar) without any positional restraints, as this ensemble accurately reflects physiological conditions (constant temperature and pressure) (Domicevica et al. 2018). Structural stability and conformational dynamics were evaluated by calculating the root‐mean‐square deviation (RMSD) of Cα atoms, radius of gyration (*R*_g_), and the number of intermolecular hydrogen bonds.

### 2.9 Normal Mode Analysis (NMA)

NMA efficiently identifies vibrational modes and protein flexibility, aiding in the study of atom movements within molecules (Bahar et al. 2010). To validate the motions of atoms and molecules in vaccination complexes with TLR2 and TLR4, the iMODS server was utilized for kinetic simulation analyses (López‐Blanco et al. 2014).

### 2.10 MM‐PBSA Calculation

The gmx_MMPBSA v1.62 tool was used to determine the binding energy between the vaccine and receptors (Genheden and Ryde 2015). By employing the molecular mechanics/Poisson–Boltzmann surface area (MM‐PBSA) method, the binding energy was calculated based on the latest 10 ns trajectory.

$$△G_{0\mathrm{bind},\mathrm{solv}}^{0}=△G_{0\mathrm{bind},\mathrm{solv}}^{0}+{△G}_{\mathrm{solv},\mathrm{complex}}^{0}-({△G}_{\mathrm{solv},\mathrm{ligand}}^{0}+{△G}_{\mathrm{solv},\mathrm{receptor}}^{0}),$$

$${△G}_{\mathrm{solv}}^{0}={△G}_{\mathrm{electrostatic},c=80}^{0}-{△G}_{\mathrm{electrostatic},c=1}^{0}+{△G}_{\mathrm{hydrophobic}}^{0},$$

$${△G}_{\mathrm{vaccum}}^{0}={△E}_{\mathrm{molecular}\;\mathrm{mechanics}}^{0}-T\times {△S}_{\mathrm{nomal}\;\mathrm{mode}\;\mathrm{analysis}}^{0}.$$

### 2.11 Immune Simulation and Population Coverage

The C‐IMMSIM v1.0 server used default HLA parameters (HLA‐A*01:01, HLA‐B*07:02, and HLA‐DRB1*01:01) to simulate how the human immune system would respond to the vaccine (Rapin et al. 2010). The administration protocol involved administering 1000 vaccine units every 4 weeks for three injections as per prior studies, with 1, 84, and 168 time steps for each injection, totaling 1050 steps. The evaluation of immunogenicity centered on three principal metrics: (1) T‐cell immunity (peak CD8⁺ CTL count, CD4⁺ Th1/Th2 ratio, and IFN‐γ levels), (2) B‐cell/antibody responses (maximum immunoglobulin G [IgG] titer, affinity maturation, neutralizing antibody titers), and (3) the persistence of memory response.

To account for population coverage variability essential in vaccine design (Adhikari et al. 2018), we utilized the IEDB Population Coverage tool to assess the global population coverage of all HLA alleles corresponding to the selected MHC‐I and MHC‐II epitopes (Bui et al. 2006). The chimeric epitope vaccine ZnuA101 against *Staphylococcus aureus* was validated in animal models and clinical trial samples, confirming that it preserves native epitope conformations and induces specific protective antibodies (X. Zhang et al. 2025). Its sequence was used for immunogenicity simulations to benchmark against our vaccine.

### 2.12 mRNA Precursor Construction and Secondary Structure Prediction

To design vaccines with better results, we used the Jcat server to optimize the vaccines' DNA sequences, which can avoid specific restriction enzyme cleavage sites and transcription terminators, and then calculated the CAI values to discover the most suited sequences (Grote et al. 2005). The mRNA was enhanced with the Kozak region for stability and translation efficiency (Kim et al. 2022), tPA signal peptide for protein release and antigen presentation (Kou et al. 2017), MITD for MHC‐I targeting, and a TAA termination codon (Kreiter et al. 2008). mRNA stability was further supported by integrating CMV (Kowalski et al. 2018) and human growth hormone (Kauffman et al. 2016) sequences into the UTRs. The Transcription Tool was utilized to transcribe the DNA sequence into mRNA, with the RNAfold service predicting the secondary structure of the mRNA.

After codon optimization, BamHI and XhoI cleavage site sequences from plasmid Expression by T7 RNA polymerase (pET)‐28a (+) were added to the optimized codon sequences. The final sequences were then incorporated into the pET‐28a (+) vector through GenSmart Design to form the expression vector.

## 3 Results

### 3.1 Epitopes Identification

Following the obtaining of nine streptococci TMPC protein sequences from the NCBI database, we thoroughly studied the top‐ranked epitopes, looking for robust antigenicity, appropriate immunogenicity, non‐allergenicity, and non‐toxicity (Figure S1). The results of epitope predictions and their corresponding MHC molecular alleles using the MHC prediction tool and TepiTool are shown in Tables S1 and S2. BLAST analysis revealed that one LBL epitope (DTGGVDDKSFNOSAWE) matches (*E* ≤ 1e − 4) that would pose a risk for off‐target immune responses (Table S6). Therefore, we excluded this antigenic determinant. We identified 9 CTL epitopes, 15 HTL epitopes, and 12 B‐cell epitopes (Tables 1, 2, 3).

**Table 1:** The selected cytotoxic T lymphocyte epitopes for the vaccine construct.

Name	Peptide	Rank%	Antigen Score	Allergy	Toxicity	Immunogenicity score	Human homology
C1	YAAGADVVY	0.24	0.7557	(−)	(−)	0.16474	(−)
C2	NESEYATNL	0.113	0.4995	(−)	(−)	0.10926	(−)
C3	IESEVISRF	0.189	0.572	(−)	(−)	0.10315	(−)
C4	YAAGADIVY	0.248	0.714	(−)	(−)	0.24222	(−)
C5	YAAGADVIY	0.29	0.62	(−)	(−)	0.21838	(−)
C6	FSQAATNGY	0.088	0.4514	(−)	(−)	0.09075	(−)
C7	KLVYGVGYK	0.069	0.7908	(−)	(−)	0.10798	(−)
C8	AQDNTGINY	0.195	1.3338	(−)	(−)	0.17893	(−)
C9	KILDGSITV	0.011	0.6169	(−)	(−)	0.03099	(−)

**Table 2:** The selected helper T lymphocyte epitopes for the vaccine construct.

Name	Peptide	Score	Rank%	Antigen Score	Allergy	Toxicity	IFN‐γ	Human homology
H1	SNFVLASSLKKVGGT	0.8599	0.06	0.4811	(−)	(−)	(+)	(−)
H2	SEKVWVLGVDRDQNA	0.6378	0.39	0.4087	(−)	(−)	(+)	(−)
H3	VLGVDRDQNAEGKYK	0.8346	0.46	1.1936	(−)	(−)	(+)	(−)
H4	EKGRTIAATQYAAGA	0.2651	0.42	0.4708	(−)	(−)	(+)	(−)
H5	TTSNLSEDAKKAVED	0.8607	0.51	0.5335	(−)	(−)	(+)	(−)
H6	VIGVDRDQVDEGKYT	0.5488	0.56	0.4522	(−)	(−)	(+)	(−)
H7	NPEKGRTIAATQYAA	0.2035	0.85	0.4749	(−)	(−)	(+)	(−)
H8	GVGFALSDSVKKAAK	0.8939	0.03	0.5183	(−)	(−)	(+)	(−)
H9	GKKSNFVLASSLKKV	0.8635	0.06	0.4756	(−)	(−)	(+)	(−)
H10	LDEAVSNDYKLIFGV	0.9136	0.28	0.4659	(−)	(−)	(+)	(−)
H11	VLGVDRDQKAEGEYT	0.8695	0.47	1.0027	(−)	(−)	(+)	(−)
H12	YVIIDDRIEGQKNVA	0.8115	0.74	0.9403	(−)	(−)	(+)	(−)
H13	SQYATNLDEAVSNDY	0.4846	0.94	0.6601	(−)	(−)	(+)	(−)
H14	GVDLTTTNLSEDAKK	0.0294	0.47	1.0419	(−)	(−)	(+)	(−)
H15	NYVIIDDVIEGQKNV	0.8118	0.74	0.4422	(−)	(−)	(+)	(−)

**Table 3:** The selected linear B‐lymphocyte epitopes for the vaccine construct.

Name	Sequence	Start position	Score	Antigen Score	Allergy	Toxicity	Human homology
B1	YVIIDDEIKGQKNVAS	126	0.91	1.0576	(−)	(−)	(−)
B2	AAIGLAACGNRASKSD	15	0.85	1.1675	(−)	(−)	(−)
B3	AAFGLAACGNRASRSD	15	0.83	1.2566	(−)	(−)	(−)
B4	FGLAACGNRASKSDNK	17	0.86	1.6234	(−)	(−)	(−)
B5	AASSDYKLVFGIGFAL	95	0.84	1.4517	(−)	(−)	(−)
B6	RSSRNAASSSDVKTKA	25	0.87	1.784	(−)	(−)	(−)
B7	DVVYQAAGGTGAGVFA	223	0.82	1.1239	(−)	(−)	(−)
B8	AAIGLAACGNRASRKD	15	0.84	1.1913	(−)	(−)	(−)
B9	VDEGKYTSKDGKESNF	264	0.82	1.4523	(−)	(−)	(−)
B10	YVIIDDRIEGQKNVAS	128	0.94	1.0039	(−)	(−)	(−)
B11	AAIGLAACGNRASKKD	15	0.85	1.2206	(−)	(−)	(−)
B12	AGGTGAGVFSEAKDLN	232	0.84	1.195	(−)	(−)	(−)

### 3.2 Conservative Analysis and Multiple Sequence Alignment

**Figure 1:**
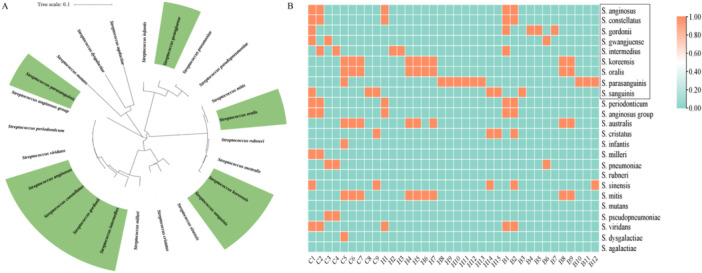
Phylogenetic analysis of TMPC‐positive *Streptococcus* species and epitope conservation. (A) Phylogenetic tree of TMPC proteins from 24 TMPC‐positive *Streptococcus* species with potential human pathogenicity. The tree was constructed based on multiple sequence alignment using the Neighbor‐Joining method with 1000 bootstrap replicates. Species highlighted in green indicate *Streptococcus* strains that are significantly enriched in gastric cancer patients. (B) Heatmap showing the conservation of predicted CTL, HTL, and B‐cell epitopes across the 24 TMPC‐positive *Streptococcus* species, illustrating both shared conserved epitopes and species‐preferential epitope patterns. C1–C9, CTL epitopes; H1–H15, HTL epitopes; B1–B12, LBL epitopes. CTL, cytotoxic T lymphocyte; HTL, helper T lymphocyte; LBL, linear B‐lymphocyte; TMPC, *Treponema pallidum* membrane protein C.

A MEGA11‐based phylogenetic tree and heatmap revealed that most selected CTL, HTL, and B‐cell epitopes were highly conserved across strains, with some sites showing strain‐specific loss or variation (Figure 1).

### 3.3 Docking of T‐Cell Epitopes and MHC Molecules

**Figure 2:**
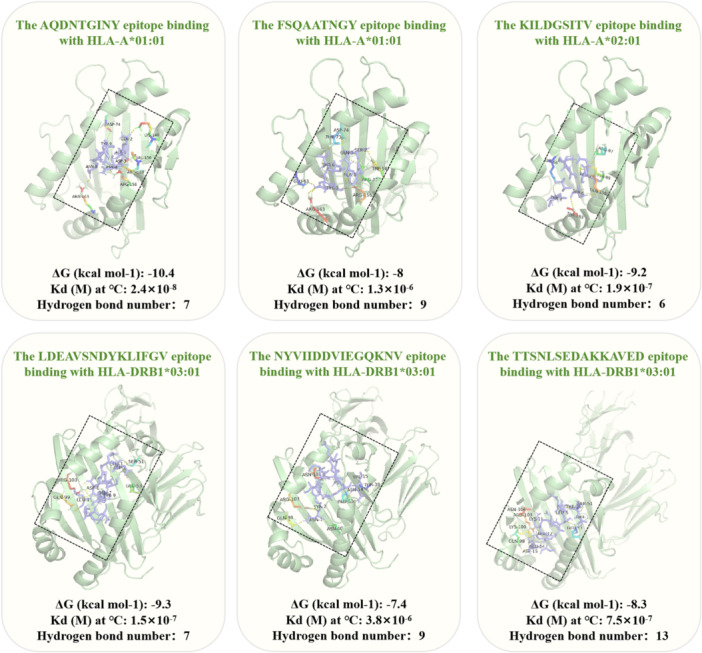
The I and II class T‐cell epitopes in the multi‐epitope are molecularly docked with their corresponding HLA alleles. This article mainly presents the main results of the molecular docking of T‐cell epitopes AQDNTGINY, FSQAATNGY, KILDGSITV, LDEAVSNDYKLIFGV, NYVIIDDVIEGQKNV, and TTSNLSEDAKKAVED with the HLA molecules. In each complex, the molecules involved in the binding (single‐letter codes and numbers) and the polar bonds (yellow dotted lines) between them are shown. Below each complex, the Δ*G*, *K*_d_, and the number of hydrogen bonds are displayed to evaluate the docking effect. HLA, Human Leukocyte Antigen.

The quality of the HLA molecule models generated with SWISS‐MODEL server was assessed using the ERRAT score and the Ramachandran Plot. The results indicated that the models had good quality (Table S5). The PRODIGY tool calculated CTL epitopes binding to MHC‐I alleles with energies from −10.4 to −7.7 kcal/mol (Table S3), while HTL epitopes bound to MHC‐II alleles with affinities ranging from −10 to −7.4 kcal/mol (Table S4). These results highlight the strong affinity of T‐cell epitopes for MHC molecules. PyMOL 2.5 software visualized the interactions between T‐cell epitopes and HLA molecules (Figure 2). Ligplot+ v2.2 illustrated significant connections between T‐cell epitopes and MHC molecules (Tables S3 and 4).

**Table 4:** Docking analysis of vaccine‐TLR/HLA combination.

Complex	Vaccine1‐HLA‐A*02:01	Vaccine1‐HLA‐DRB1*01:01	Vaccine1‐TLR2	Vaccine1‐TLR4	Vaccine2‐HLA‐A*02:01	Vaccine2‐HLA‐DRB1*01:01	Vaccine2‐TLR2	Vaccine2‐TLR4	PepO‐TLR2	PepO‐TLR4	Hemolysin‐TLR2	Hemolysin‐TLR4
ClusPro												
	−805.4	−836.6	−749.4	−776.4	−753.3	−1015.4	−722.7	−697	−743	−559.5	−825.6	−753.8
	−919.7	−995	−873	−999.7	−903.9	−1015.4	−789	−801.8	−842.4	−813.8	−990.4	−847.9
HADDOCK score	−409.2 ± 3.6	−623.8 ± 2.4	−160.6 ± 1.4	−181.9 ± 5.3	−398.3 ± 1.9	−646.6 ± 3.9	−177.2 ± 2.0	−167.1 ± 3.8	−151.5 ± 4.0	1382.1 ± 3.5	−127.9 ± 5.2	−142.7 ± 1.0
Van der Waals energy	−199.6 ± 5.2	−311.3 ± 3.2	−78.1 ± 0.6	−85.8 ± 8.2	−184.8 ± 7.2	−344.5 ± 1.9	−65.4 ± 2.2	−93.5 ± 6.9	−67.3 ± 1.4	1474.0 ± 4.9	−72.0 ± 4.0	−71.0 ± 6.3
Electrostatic energy	−613.3 ± 13.1	−1030.2 ± 12.4	−85.5 ± 6.8	−359.6 ± 20.9	−974.6 ± 38.6	−1029.7 ± 14.4	−465.2 ± 15.3	−474.0 ± 42.0	−293.9 ± 34.6	−316.9 ± 20.2	−60.9 ± 11.6	−133.6 ± 31.6
Desolvation energy	−87.0 ± 2.4	−106.5 ± 3.5	−65.4 ± 0.4	−24.2 ± 1.6	−18.6 ± 3.4	−96.1 ± 5.1	−18.7 ± 1.2	21.2 ± 2.5	−25.4 ± 4.4	−28.6 ± 3.2	−43.7 ± 2.5	−45.0 ± 1.7
Buried Surface Area	4912.1 ± 57.3	7779.4 ± 103.0	1885.2 ± 31.8	2955.3 ± 117.7	5257.3 ± 104.1	8968.7 ± 81.5	2151.5 ± 25.5	3406.4 ± 72.4	2163.2 ± 56.3	3658.0 ± 41.7	1550.0 ± 39.6	2010.3 ± 27.3

Abbreviations: HLA, Human Leukocyte Antigen; TLR, Toll‐Like Receptor.

### 3.4 Construction of a Multi‐Epitope Vaccine

**Figure 3:**
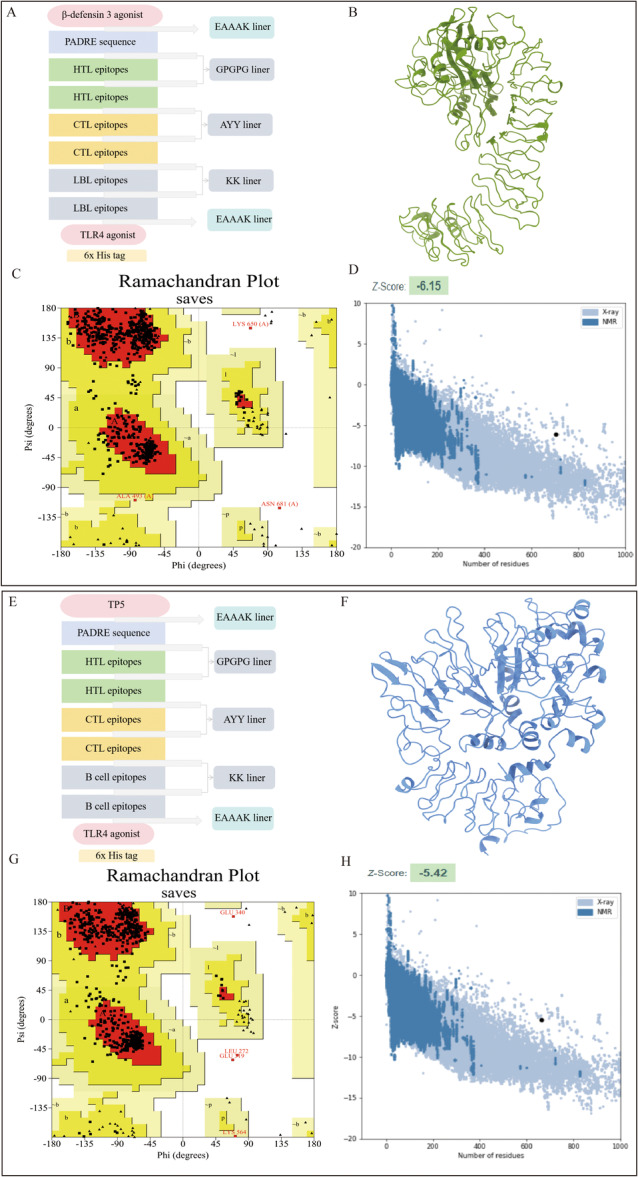
(A, E) Vaccine 1 and Vaccine 2 construction. (B, F) 3D structure of Vaccine 1 and Vaccine 2. (C, G) The Ramachandran plots of the refined 3D models of Vaccine 1 and Vaccine 2 generated by the PROCHECK server: The red regions are the most favored regions, the dark yellow and light yellow regions are the additionally allowed and generously allowed regions, and the white regions are the disallowed regions. (D, H) The *Z* score plot of the refined 3D model of Vaccine 1 generated by the ProSA‐web server. 3D, three‐dimensional; CTL, cytotoxic T lymphocyte; HTL, helper T lymphocyte; LBL, linear B‐lymphocyte; NMR, nuclear magnetic resonance; TLR, Toll‐Like Receptor; TP5, thymopentin.

The ideal vaccine should contain conserved epitopes, be multivalent, and induce both cellular and humoral immune responses in the host. The vaccine's immunogenicity can be increased by adding appropriate adjuvants. As a result, we formulated two vaccines with different adjuvants: (1) β‐Defensin‐3‐EAAK‐PADRE‐GPGPG‐HTL epitopes‐AAY‐CTL epitopes‐KK‐LBL epitopes‐EAAK‐RS09 (TLR4 agonist) (Figure 3A) and (2) TP5‐EAAK‐PADRE‐GPGPG‐HTL epitopes‐AAY‐CTL epitopes‐KK‐LBL epitopes‐EAAK‐RS09 (TLR4 agonist) (Figure 3E).

### 3.5 Prediction of Physicochemical Properties

Vaccine 1 had 705 amino acids, a molecular weight of 72.48 kDa, isoelectric point (pI) 9.41, an instability index of 21.04 (indicating stability), an aliphatic index of 68.26, and GRAVY −0.451. It estimated half‐life exceeds 30 h in mammalian cells, over 20 h in yeast, and more than 10 h in *E. coli*. Vaccine 2 comprised 665 amino acids, a molecular weight of 68.00 kDa, pI 9.330, an instability index of 18.54, an aliphatic index of 68.26, and GRAVY −0.444. The estimated half‐lives were 1 h in human cells, 2 min in yeast, and 2 min in *E. coli*. VaxiJen 2.0 scored the antigenicity of the developed vaccinations at 0.9267 and 0.9347, respectively. Furthermore, assessments using AllerTOP v2.0 and Toxin Pred v2.0 validated the vaccines' non‐allergenic and non‐toxic properties. As per Protein‐Sol projections, Vaccines 1 and 2 displayed solubility values of 0.672 and 0.626, respectively, surpassing the solubility threshold of 0.45. This suggests the vaccines exhibit higher solubility than the usual soluble *E. coli* protein.

### 3.6 Secondary and 3D Structure Predictions, 3D Structure Refinement, and Validation

PSIPRED 4.0 analysis revealed that Vaccine 1 consisted of 39.1% α‐helix, 13.9% β‐strand, and 47.0% random coil, while Vaccine 2 comprised 39.8% α‐helix, 11.1% β‐strand, and 49.1% random coil (Figure S2). The initial 3D structures were constructed using AlphaFold2 and Chai 1, followed by energy minimization and GalaxyRefine refinement to optimize structural stability (Figure S5). Structural validation via SAVE 6.1 (including Ramachandran plots, ERRAT scores, *Z* scores, and Verify3D; Table S7) showed that the Chai 1–predicted models exhibited superior overall quality compared with AlphaFold2 models; thus, they were selected for further analyses. The Ramachandran plots indicated that 89.6% and 88.6% of residues in the preferred regions and > 98% in allowed regions confirmed good stereochemical quality (Figure 3C,G). ERRAT scores (83.52 and 90.78) and ProSA *Z* scores (−6.15 and −5.42) (Figures 3D,H and S3A,C) further supported the reliability of the models. In the Verify3D profiles, over 80% of residues scored > 0.1 (Figure S3B,D), demonstrating consistent and stable tertiary structures of both vaccine constructs.

### 3.7 Discontinuous B‐Cell Epitopes Prediction

The ElliPro tool predicted five discontinuous B‐cell epitopes in Vaccine 1, with scores ranging from 0.518 to 0.788 (Table S8). The epitopes contained 3–195 residues (Figure S4A–F). Vaccine 2 harbored four discontinuous epitopes with scores between 0.500 and 0.700, comprising 3–228 residues (Table S9 and Figure S4G–J). Additionally, 22 and 24 linear B‐cell epitopes were identified in Vaccines 1 and 2, respectively (Tables S10–S11). These results suggest that both vaccine constructs possess multiple antigenic regions with strong potential to elicit humoral immune responses.

### 3.8 Molecular Docking of Vaccines

**Figure 4:**
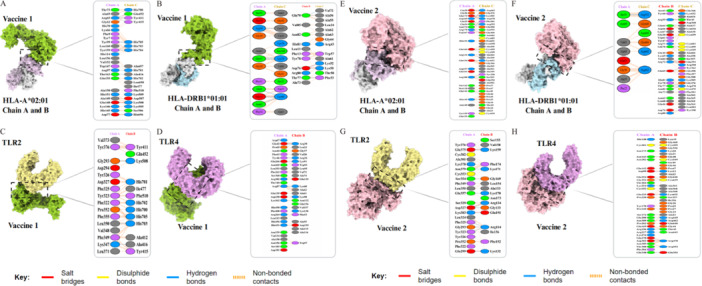
Visualization of docking and interacting residues of vaccines with HLA molecules, TLR complexes. (A) Vaccine 1‐HLA‐A*02:01 complex, (B) Vaccine 1‐HLA‐DRB1*01:01 complex, (C) Vaccine 1‐TLR 2 complex, (D) Vaccine 1‐TLR 4 complex, (E) Vaccine 2‐HLA‐A*02:01 complex, (F) Vaccine 2‐HLA‐DRB1*01:01 complex, (G) Vaccine 2‐TLR 2 complex, and (H) Vaccine 2‐TLR 4 complex. HLA, Human Leukocyte Antigen; TLR, Toll‐Like Receptor.

The top 30 docking poses of vaccine–HLA/TLR complexes were generated using the ClusPro server. On the basis of clustering scores and binding sites, the optimal models were refined with HADDOCK, and four representative conformations were obtained, from which the top‐ranked model was selected for further analysis (Tables S12–S19 and Figures S6, –S21). Docking results for vaccine–HLA/TLR interactions are summarized in Table 4. Vaccine 2 exhibited stronger binding affinity with HLA‐A*02:01 and HLA‐DRB1*01:01 compared with Vaccine 1, while both vaccines showed comparable binding energies with TLR2 and TLR4. These findings indicate that both vaccine constructs can stably interact with the selected HLA and TLR targets. The vaccine–receptor complexes were visualized using ChimeraX v1.7, and PDBsum analysis revealed numerous hydrogen bonds and salt bridges stabilizing the interactions (Figure 4A–H). To validate these results, the vaccine–TLR complexes were compared with known PepO and Hemolysin structures. The designed vaccines exhibited interaction strengths comparable to Hemolysin, supporting their binding feasibility and structural reliability (Table 4 and Figures 4 and S22).

### 3.9 MD Simulations

**Figure 5:**
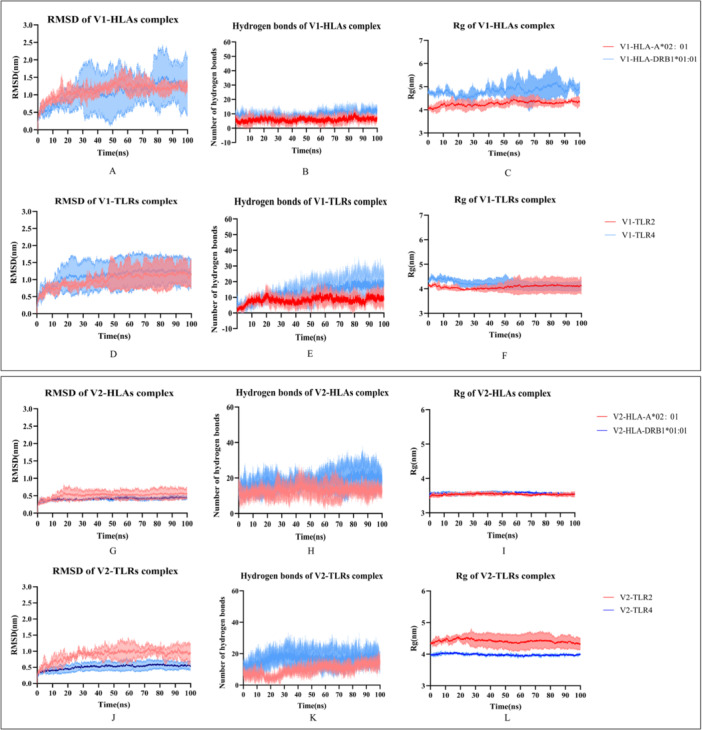
Molecular dynamics simulations of vaccine–receptor complexes. (A, B, C) The thrice RMSD, hydrogen bonds, and *R*_g_ results of Vaccine 1‐HLAs complex; (D, E, F) the thrice RMSD, hydrogen bonds, and *R*_g_ results of Vaccine 1‐TLRs complex; (G, H, I) the thrice RMSD, hydrogen bonds, and *R*_g_ results of Vaccine 2‐HLAs complex; (J, K, L) the thrice RMSD, hydrogen bonds, and *R*_g_ results of Vaccine 1‐HLAs complex. HLA, Human Leukocyte Antigen; RMSD, root‐mean‐square deviation; TLR, Toll‐Like Receptor.

A 100‐ns MD simulation was performed using GROMACS v2023.3, followed by analyses of RMSD, radius of gyration (*R*_g_), and hydrogen bonds to evaluate the structural stability and interaction dynamics of the vaccine–receptor complexes. Each simulation was replicated three times to ensure reproducibility. RMSD analysis revealed that the Vaccine 1–HLA/TLR complex exhibited pronounced fluctuations and only stabilized after 50 ns, whereas Vaccine 2 reached equilibrium by 20 ns, indicating greater conformational stability (Figure 5A,G,D,J). Vaccine 2 consistently exhibited significantly lower RMSD values than Vaccine 1 across all HLA and TLR complexes (*p* < 0.0001), indicating improved structural stability (Figure S23A,B). Hydrogen‐bond analysis showed that Vaccine 2 formed more hydrogen bonds with HLA than Vaccine 1 (Figure 5B,H), implying stronger interactions. In contrast, in TLR complexes, both vaccines exhibited comparable hydrogen‐bond numbers, with Vaccine 2 showing a minor advantage (Figure 5E,K). Vaccine 2 displayed lower *R*_g_ values than Vaccine 1 in HLA complexes (Figures 5C,J and S23C), suggesting a more compact and stable structure. In contrast, Vaccine 2 exhibited lower RMSF values, indicating improved rigidity and stability, particularly in HLA‐A*02:01 and TLR4 complexes. Vaccine 1 demonstrated enhanced structural flexibility, whereas Vaccine 2 exhibited superior overall stability, especially within HLA complexes. In TLR complexes, *R*_g_ values were similar, though Vaccine 2 remained slightly more compact (Figures 5D,L and S23D). RMSF analysis (Figure S24A–H) demonstrated higher residue flexibility in Vaccine 1, particularly when bound to HLA‐DRB1*01:01 and TLR2. In contrast, Vaccine 2 maintained lower RMSF values, reflecting enhanced rigidity and stability, especially in HLA‐A*02:01 and TLR4 complexes. Overall, Vaccine 1 exhibited greater structural flexibility, while Vaccine 2 showed superior overall stability, particularly within HLA complexes.

### 3.10 Normal Mode Analysis

The IMODS server was used to evaluate the variability, flexibility, and stability of vaccine–receptor complexes, confirming overall rigidity, stability, and low deformability. NMA revealed distinct dynamic profiles between Vaccine 1 and Vaccine 2. For Vaccine 1, low dominant‐mode eigenvalues were observed upon binding to HLA‐A*02:01 and HLA‐DRB1*01:01 (1.5 × 10⁻⁶ and 4.6 × 10⁻⁶, respectively), with fluctuations mainly restricted to the peptide‐binding groove, suggesting rigid complex formation (Figures S25 and S26). In contrast, TLR2 and TLR4 complexes showed higher eigenvalues (4.3 × 10⁻⁶ and 4.5 × 10⁻⁶) and pronounced extracellular‐domain flexibility, with the first three modes contributing 60%–70% of total variance (Figures S27 and S28). Vaccine 2 exhibited markedly higher eigenvalues across all receptors (HLA, 3.3 × 10⁻⁵ and 4.4 × 10⁻⁵; TLR, 8.0 × 10⁻⁶ and 1.5 × 10⁻⁵), indicating enhanced domain flexibility, particularly in TLR complexes (Figures S29–S32). These results suggest that peptide‐binding groove fluctuations reflect a stabilized MHC conformation conducive to antigen presentation. Moreover, Vaccine 2 interacted more strongly with TLR2 than with TLR4. For comparison, PepO and hemolysin exhibited higher rigidity and stability (Figures S33–S36), while our vaccines also maintained strong yet moderate flexibility, supporting effective receptor engagement.

### 3.11 MM‐PBSA Calculation

To ensure the robustness of our predictions, MM‐PBSA calculations were performed using three independent MD trajectories (Tables S20–S22). The MM‐PBSA results showed that Vaccine 2 consistently exhibited significantly more favorable total binding free energies than Vaccine 1 across all receptor complexes (*p* < 0.0001) (Table 5 and Figure S23E,F). Vaccine 1 displayed its strongest binding to HLA‐DRB101:01 and TLR4 (−141.75 and −150.90 kcal/mol), but much weaker affinities for HLA‐A02:01 and TLR2 (−69.77 and −94.79 kcal/mol). In contrast, Vaccine 2 maintained uniformly strong binding across all receptors, with comparable or improved affinities for HLA‐DRB1*01:01 and TLR4 (−188.45 and −150.51 kcal/mol). Energy decomposition further confirmed this advantage: Vaccine 2 demonstrated significantly stronger van der Waals contributions in all complexes except HLA‐DRB1*01:01 (Figure S23G,H).

**Table 5:** MM‐PBSA of vaccine‐TLR2/TLR4/HLA‐A*02:01/HLA‐DRB1*01:01 delta (complex–receptor–ligand).

	Average						
Complex	ΔVDWAALS	ΔEEL	ΔEPB	ΔENPOLAR	ΔGGAS	ΔGSOLV	ΔTotal
Vaccine1‐HLA‐A*02:01	−100.20	−952.53	993.93	−10.97	−1052.73	982.96	−69.77
Vaccine1‐HLA‐DRB1*01:01	−149.95	−3656.38	3680.47	−15.89	−3806.33	3664.58	−141.75
Vaccine1‐TLR2	−125.36	−1292.12	1337.90	−15.21	−1417.48	1322.68	−94.79
Vaccine1‐TLR4	−177.02	−3105.38	3153.43	−21.93	−3282.41	3131.50	−150.90
Vaccine2‐HLA‐A*02:01	−124.52	−1796.14	1823.75	−15.58	−1920.66	1808.17	−112.48
Vaccine2‐HLA‐DRB1*01:01	−207.03	−3840.02	3882.87	−24.26	−4047.05	3858.61	−188.45
Vaccine2‐TLR2	−112.47	−1160.11	1184.60	−12.92	−1272.58	1171.69	−100.89
Vaccine2‐TLR4	−170.49	−2758.21	2798.24	−20.05	−2928.70	2778.20	−150.51

Abbreviations: HLA, Human Leukocyte Antigen; MM‐PBSA, molecular mechanics/Poisson–Boltzmann surface area; TLR, Toll‐Like Receptor.

### 3.12 Immunization Simulation and Population Coverage

**Figure 6:**
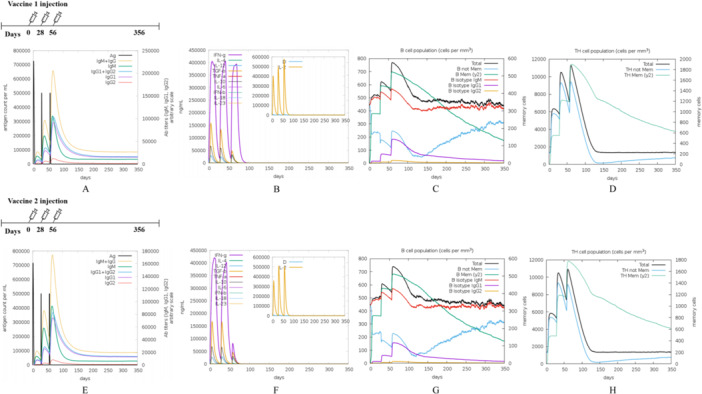
Immunization simulation results for two vaccines. (A, E) Antibody levels induced by three doses of Vaccine 1 and Vaccine 2 injections, (B, F) levels of cytokines induced by Vaccine 1 and Vaccine 2, (C, G) population of B cells induced by Vaccine 1 and Vaccine 2, and (D, H) Population of helper T(TH) cells with Vaccine 1 and Vaccine 2.

Elevated primary immunoglobulin M (IgM) antibody levels following each injection of the epitope‐based peptide vaccination demonstrated a strong immune response. Moreover, the rise in B‐cells correlated with increased levels of IgG1 + IgG2, IgM, and IgG + IgM antibodies (Figure 6A,E); additionally, memory B‐cell counts increased and remained stable for a year (Figure 6C,G). Furthermore, helper T cells increased during the second and third immunological responses (Figure 6D,H). The results show that immunization can activate many cytokines, such as IFN‐γ, interleukin‐4 (IL‐4), IL‐2, and others (Figure 6B,F). In contrast, Vaccine 2 resulted in a reduction in Interferon levels after the third stimulation. Both vaccines successfully stimulated the production of various immune cells and a range of cytokines, demonstrating the induction of effective humoral and cellular immunity in the organism. The vaccine's population coverage, based on MHC alleles matching the epitope sequences, varies among ethnic groups. The vaccine protects 95.67% globally and over 99% in most regions (Figure S37 and Table S23). In addition, we evaluated the immunogenicity of the *S. aureus* epitope vaccine ZnuA101 for comparison. Vaccine 2 consistently elicited the strongest and most durable responses, with higher antibody titers, more robust T‐cell activation, and greater memory induction than Vaccine 1 and ZnuA101. Its enhanced cytokine secretion and balanced Th1/Th2 profile further reflect more efficient innate–adaptive immune coordination. Vaccine 1 showed weaker cellular responses, whereas ZnuA101 induced moderate but less sustained immunity. Overall, Vaccine 2 demonstrates the greatest immunogenicity and long‐term protective potential (Figure S38).

### 3.13 Circular mRNA Precursor Construction and Secondary Structure Prediction

**Figure 7:**
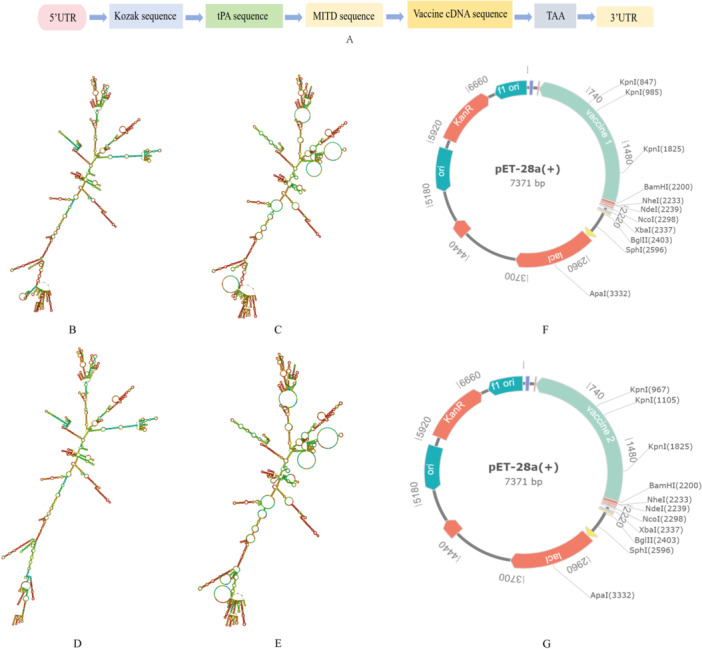
mRNA vaccine construction. (A) The design of mRNA vaccines, (B, D) MFE secondary structure of Vaccine 1 and Vaccine 2, (C, E) centroid secondary structure, and (F, G) vaccines into *Escherichia coli* expression vector pET28a (+). 5′UTR, 5′‐untranslated region; cDNA, complementary DNA; MFE, minimum free energy; MHC, major histocompatibility complex; MITD, MHC‐I targeting domain; mRNA, messenger RNA; pET, plasmid Expression by T7 RNA polymerase; TAA, tumor‐associated antigen; tPA, tissue plasminogen activator.

Both vaccines underwent reverse translation and optimization using the Jcat server, resulting in a CAI value of 1.0 and GC contents within the ideal range for good density and thermal stability (49.78 for Vaccine 1 and 49.73 for Vaccine 2). RNAfold analysis indicated the free energy of the Vaccine 1 and Vaccine 2 mRNA were −1018.10 and −979.60 kcal/mol, respectively. The centroid secondary structure revealed a minimum free energy of −855.49 and −804.80 kcal/mol, confirming the stability of the multiepitope mRNA vaccine. The mRNA vaccine sequence was then integrated into the pET‐28a (+) plasmid between the BamHI and XhoI sites to produce the expression vector (Figure 7).

## 4 Discussion

*S. anginosus*, *S. constellatus*, *S. gordonii*, *S. gwangjuense*, *S. intermedius*, *S. koreensis*, *S. oralis*, *S. parasanguinis*, and *S. sanguinis* are significant opportunistic pathogens in the human oral cavity, responsible for periodontal lesions and colonizing the stomach mucosa, ultimately leading to a chronic phase of persistent and aggravated gastritis (Fu et al. 2024). *S. anginosus* has become an important factor in the occurrence of gastric cancer. However, the current clinical methods are limited to species‐level detection and lack reliable noninvasive measurement methods (Pilarczyk‐Zurek et al. 2022; Qian et al. 2024). mRNA vaccine targeting a conserved virulence antigen facilitates noninvasive monitoring of anti‐TMPC antibodies and induces durable, pathogen‐specific immunity that neutralizes virulence without disrupting the microbiota, while avoiding the transient efficacy and resistance issues associated with antibiotics (Gote et al. 2023). TMPC was selected as the vaccine target due to its essential pore‐forming function, conserved and highly immunogenic epitopes, minimal homology to human proteins, and superior preclinical efficacy in reducing gastric colonization. Unlike ANXA2, a host protein that indirectly participates in bacterial adhesion, and has potential autoimmune risks.

The application of immunoinformatics in vaccine development now focuses on designing multi‐epitope structures to induce highly specific immune responses (G. Zhang et al. 2023). Peptide‐based mRNA vaccines show promise due to their stability, simplicity, and cost‐effectiveness (G. Zhang et al. 2023). TMPC, an ABC‐transporter lipoprotein essential for purine transport and virulence regulation, serves as a central factor in gastric colonization and carcinogenesis, making it a rational target for vaccine development (Deka et al. 2006; Fu et al. 2024). By analyzing TMPC from nine closely related gastric‐colonizing *Streptococcus* species, we identified a set of conserved epitopes that provides broad strain coverage while maintaining specificity. Rigorous screening confirmed their safety and immunogenic potential, and IFN‐γ‐inducing HTL epitopes were prioritized to enhance anti‐*S. anginosus* responses. Most CTL, HTL, and B‐cell epitopes showed high conservation across the SAG, with only minor strain‐specific variations, and the selected T‐cell epitopes achieved an estimated global HLA coverage of 95.67%.

Our vaccine constructs integrate multiple CTL, HTL, and B‐cell epitopes from the SAG TMPC protein, supported by a rational linker and adjuvant architecture designed to enhance immunogenicity. Flexible linkers (AAY, GPGPG, and KK) were incorporated to minimize steric hindrance and preserve the exposure of adjacent epitopes (Ahmad et al. 2024; Ma et al. 2025), while the rigid EAAAK linker improved structural stability by reducing conformational interference between the adjuvant and the vaccine core (X. Chen et al. 2013). PADRE further strengthens CD4⁺ T‐cell help, a key driver of robust CTL and B‐cell activation (Franke et al. 1999). Leveraging TLR2 and TLR4 signaling is particularly advantageous given their central roles in pathogen recognition and early immune activation (Mukherjee et al. 2016). Previous studies indicate that adjuvants acting through these pathways—such as the TLR2‐targeting TP5, the immunomodulatory peptide human β‐defensin‐3, and the TLR4 agonist RS09—can substantially enhance antigen presentation and downstream T‐cell responses (Dhople et al. 2006; Ito et al. 2017; Wei et al. 2021). Incorporating such adjuvants, therefore, represents a rational approach to strengthening both innate and adaptive immunity in multiepitope vaccine constructs. Our results further support that selecting various adjuvants can enhance the vaccine's ability to induce immune responses. However, the presence of multiple epitopes and adjuvants can lead to potential challenges, such as protein misfolding and low translational efficiency (Scheiblhofer et al. 2017). To overcome these issues, we have employed codon optimization tools to refine our design, thereby enhancing the translational efficiency of our mRNA vaccine (Pardi et al. 2015; C. Zhang et al. 2026). The expression and integrity of the protein will be confirmed in future studies through Western blot experiments. ZnuA101, an experimentally validated *S. aureus* epitope vaccine, served as an important benchmark. Compared with ZnuA101, our designed Vaccine 2 exceeded ZnuA101 in antibody generation, T‐cell activation, and memory formation, indicating a stronger and more durable protective profile. In the face of mRNA vaccine delivery challenges, lipid nanoparticles (LNPs) are efficient carriers of mRNA. They protect mRNA from degradation in harsh extracellular environments, enhance vaccine efficacy by promoting mRNA cellular uptake through cell membrane interactions (Hariri et al. 2025). However, challenges persist in optimizing cellular uptake, preventing premature mRNA release, and mitigating immune responses to the delivery system. Addressing LNP stability during storage and transport, along with reducing production costs, is essential for the broader application of mRNA vaccines (Li et al. 2025).

The findings of this study demonstrate that the engineered vaccines possess elevated predicted immunogenicity, robust antigenicity, advantageous safety profiles (non‐toxic and non‐allergenic), structural stability, and considerable binding affinity for TLRs, indicating the potential to provoke both innate and adaptive immune responses. Immunoinformatics‐based immune simulations also predicted the production of IgM antibodies, a range of cytokine responses, and long‐lasting memory B cells, which supports the idea that the vaccines can boost the immune system. It is important to note that in silico immunoinformatics and immune simulation methods are inherently predictive and use simplified computer models that cannot fully capture the complexity, diversity, and regulatory dynamics of the human immune system or the tumor microenvironment in vivo. The simulation results are not perfect, but they are consistent and stable, which gives us a good theoretical framework and useful information for future experiments. Future studies will focus on strict experimental validation to confirm the predicted immune responses and therapeutic potential of the proposed vaccines. This will include tests for peptide–MHC binding, ELISPOT analyses, and tests of immunogenicity and safety in living things.

## 5 Conclusion

Computer‐aided research methods for designing multiepitope vaccines have been successfully applied in the design of various vaccines. In this study, two multiepitope mRNA vaccines, Vaccine 1 and Vaccine 2, were designed using immunoinformatics methods. The predicted characteristics of these vaccines include antigenicity, non‐allergenicity, stability, and the ability to induce responses in T cells, helper T cells, B cells, and IFN‐γ. Each vaccine has its advantages and can serve as a candidate vaccine for preventing gastric cancer.

## Author Contributions

**Fei Zhu:** writing – review and editing, methodology, conceptualization. **Yuying Luo:** writing – original draft, data curation, visualization. **Ziyou Zhou:** resources, investigation. **Rongliu Qin:** formal analysis. **Shiyang Ma:** supervision. **Yizhong Xu:** validation. **Jie Chen:** project administration, funding acquisition. **Pinhua Pan:** project administration, funding acquisition.

## Ethics Statement

The authors have nothing to report.

## Conflicts of Interest

None declared.

## Supporting information

Supplementary_Material.

Table_S6.

## Data Availability

Data will be made available on request.
