# Supplementary material for: Computational Validation of Multi‐Epitope mRNA Vaccine Targeting Streptococcus anginosus Surface Protein (TMPC) as an Effective Alternative Treatment to Reduce Gastric Cancer
Source: Microbiologyopen. 2026 Feb 10;15(1):e70230. doi: 10.1002/mbo3.70230 (PMC12890581; doi:10.1002/mbo3.70230)
Supplement: Supplementary file 1 — Supplementary_Material. [file MBO3-15-e70230-s001.docx]

# 1. Supplementary Data

## 1.1 Test Tools Website

| Tools | Website |
| --- | --- |
| NetMHCpan4.1 server | https://services.healthtech.dtu.dk/services/NetMHCpan-4.1/ |
| MHC-II binding prediction tool | http://tools.iedb.org/mhcii/ |
| ABCpred | http://crdd.osdd.net/raghava/abcpred/ |
| TepiTool server | http://tools.iedb.org/tepitool/ |
| VaxiJen 2.0 server | https://www.ddg-pharmfac.net/vaxijen/VaxiJen/VaxiJen.html |
| AllerTOP v. 2.0 | https://www.ddg-pharmfac.net/AllerTOP/method.html |
| ToxinPred | https://webs.iiitd.edu.in/raghava/toxinpred/protein.php |
| MHC I immunogenicity | http://tools.iedb.org/immunogenicity/ |
| The IFN epitope website | http://crdd.osdd.net/raghava/ifnepitope/predict.php |
| The RCSB PDB website | https://www.rcsb.org/ |
| ProSA-web | https://prosa.services.came.sbg.ac.at/prosa.php |
| SAVES v6.1 | https://saves.mbi.ucla.edu/ |
| ClusPro 2.0 server | https://cluspro.org/help.php |
| SOLpro server | https://protein-sol.manchester.ac.uk/ |
| ToxinPred 2.0 | https://webs.iiitd.edu.in/raghava/toxinpred2/ |
| ProtParam | https://web.expasy.org/protparam/ |
| PSIPRED 4.0 server | http://bioinf.cs.ucl.ac.uk/psipred/ |
| Alphafold | https://alphafold.ebi.ac.uk/ |
| GalaxyRefine server | https://galaxy.seoklab.org/cgi-bin/submit.cgi?type=REFINE |
| ElliPro server | http://tools.iedb.org/ellipro/ |
| PDBsum | https://www.ebi.ac.uk/thornton-srv/databases/pdbsum/ |
| iMODS server | https://imods.iqf.csic.es/ |
| C-ImmSim server | https://kraken.iac.rm.cnr.it/C-IMMSIM/index.php?page=1 |
| Population Coverage tool | http://tools.iedb.org/population/ |
| Jcat server | http://www.prodoric.de/JCat |
| RNAfold | https://rna.tbi.univie.ac.at/cgi-bin/RNAWebSuite/RNAfold.cgi |

## 1.2 Vaccines construction

Multi-epitope vaccine 1 core sequence:

GIINTLQKYYCRVRGGRCAVLSCLPKEEQIGKCSTRGRKCCRRKKEAAAKAKFVAAWTLKAAAGPGPGSNFVLASSLKKVGGTGPGPGSEKVWVLGVDRDQNAGPGPGEKGRTIAATQYAAGAGPGPGTTSNLSEDAKKAVEDGPGPGVIGVDRDQVDEGKYTGPGPGNPEKGRTIAATQYAAGPGPGGVGFALSDSVKKAAKGPGPGGKKSNFVLASSLKKVGPGPGLDEAVSNDYKLIFGVGPGPGVLGVDRDQKAEGEYTGPGPGYVIIDDRIEGQKNVAGPGPGSQYATNLDEAVSNDYGPGPGGVDLTTTNLSEDAKKGPGPGNYVIIDDVIEGQKNVGPGPGVLGVDRDQNAEGKYKAAYYAAGADVVYAAYNESEYATNLAAYIESEVISRFAAYYAAGADIVYAAYYAAGADVIYAAYFSQAATNGYAAYKLVYGVGYKAAYAQDNTGINYAAYKILDGSITVKKYVIIDDEIKGQKNVASKKAAIGLAACGNRASKSDKKFGLAACGNRASKSDNKKKAASSDYKLVFGIGFALKKRSSRNAASSSDVKTKAKKDVVYQAAGGTGAGVFAKKAAIGLAACGNRASRKDKKVDEGKYTSKDGKESNFKKYVIIDDRIEGQKNVASKKAAIGLAACGNRASKKDKKAGGTGAGVFSEAKDLNKKAAFGLAACGNRASRSDEAAAKAPPHALSHHHHHH

Multi-epitope vaccine 2 core sequence:

RKDVYEAAAKAKFVAAWTLKAAAGPGPGSNFVLASSLKKVGGTGPGPGSEKVWVLGVDRDQNAGPGPGEKGRTIAATQYAAGAGPGPGTTSNLSEDAKKAVEDGPGPGVIGVDRDQVDEGKYTGPGPGNPEKGRTIAATQYAAGPGPGGVGFALSDSVKKAAKGPGPGGKKSNFVLASSLKKVGPGPGLDEAVSNDYKLIFGVGPGPGVLGVDRDQKAEGEYTGPGPGYVIIDDRIEGQKNVAGPGPGSQYATNLDEAVSNDYGPGPGGVDLTTTNLSEDAKKGPGPGNYVIIDDVIEGQKNVGPGPGVLGVDRDQNAEGKYKAAYYAAGADVVYAAYNESEYATNLAAYIESEVISRFAAYYAAGADIVYAAYYAAGADVIYAAYFSQAATNGYAAYKLVYGVGYKAAYAQDNTGINYAAYKILDGSITVKKYVIIDDEIKGQKNVASKKAAIGLAACGNRASKSDKKFGLAACGNRASKSDNKKKAASSDYKLVFGIGFALKKRSSRNAASSSDVKTKAKKDVVYQAAGGTGAGVFAKKAAIGLAACGNRASRKDKKVDEGKYTSKDGKESNFKKYVIIDDRIEGQKNVASKKAAIGLAACGNRASKKDKKAGGTGAGVFSEAKDLNKKAAFGLAACGNRASRSDEAAAKAPPHALSHHHHHH

## 1.3 mRNA vaccines construction

tPA sequence (UniProt ID: P00750)

MDAMKRGLCCVLLLCGAVFVSPS

MITD sequence (UniProt ID: Q8WV92）

MAKSGLRQDPQSTAAATVLKRAVELDSESRYPQALVCYQEGIDLLLQVLKGTKDNTKRCNLREKISKYMDRAENIKKYLDQEKEDGKYHKQIKIEENATGFSYESLFREYLNETVTEVWIEDPYIRHTHQLYNFLRFCEMLIKRPCKVKTIHLLTSLDEGIEQVQQSRGLQEIEESLRSHGVLLEVQYSSSIHDREIRFNNGWMIKIGRGLDYFKKPQSRFSLGYCDFDLRPCHETTVDIFHKKHTKNI

Kozak sequence

GCCATGATGG

5’UTR

GGACAGATCGCCTGGAGACGCCATCCACGCTGTTTTGACCTCCATAGAAGACACCGGGACCGATCCAGCCTCCGCGGCCGGGAACGGTGCATTGGAACGCGGATTCCCCGTGCCAAGAGTGACTCACCGTCCTTGACACG

3’UTR

TGACGGGTGGCATCCCTGTGACCCCTCCCCAGTGCCTCTCCTGGCCCTGGAAGTTGCCACTCCAGTGCCCACCAGCCTTGTCCTAATAAAATTAAGTTGCATCAAGCT

The cDNA sequence of the designed vaccine 1:

GGACAGATCGCCTGGAGACGCCATCCACGCTGTTTTGACCTCCATAGAAGACACCGGGACCGATCCAGCCTCCGCGGCCGGGAACGGTGCATTGGAACGCGGATTCCCCGTGCCAAGAGTGACTCACCGTCCTTGACACGGCCATGATGGATGGACGCTATGAAACGTGGTCTGTGCTGCGTTCTGCTGCTGTGCGGTGCTGTTTTCGTTTCTCCGTCTATGGCTAAATCTGGTCTGCGTCAGGACCCGCAGTCTACCGCTGCTGCTACCGTTCTGAAACGTGCTGTTGAACTGGACTCTGAATCTCGTTACCCGCAGGCTCTGGTTTGCTACCAGGAAGGTATCGACCTGCTGCTGCAGGTTCTGAAAGGTACCAAAGACAACACCAAACGTTGCAACCTGCGTGAAAAAATCTCTAAATACATGGACCGTGCTGAAAACATCAAAAAATACCTGGACCAGGAAAAAGAAGACGGTAAATACCACAAACAGATCAAAATCGAAGAAAACGCTACCGGTTTCTCTTACGAATCTCTGTTCCGTGAATACCTGAACGAAACCGTTACCGAAGTTTGGATCGAAGACCCGTACATCCGTCACACCCACCAGCTGTACAACTTCCTGCGTTTCTGCGAAATGCTGATCAAACGTCCGTGCAAAGTTAAAACCATCCACCTGCTGACCTCTCTGGACGAAGGTATCGAACAGGTTCAGCAGTCTCGTGGTCTGCAGGAAATCGAAGAATCTCTGCGTTCTCACGGTGTTCTGCTGGAAGTTCAGTACTCTTCTTCTATCCACGACCGTGAAATCCGTTTCAACAACGGTTGGATGATCAAAATCGGTCGTGGTCTGGACTACTTCAAAAAACCGCAGTCTCGTTTCTCTCTGGGTTACTGCGACTTCGACCTGCGTCCGTGCCACGAAACCACCGTTGACATCTTCCACAAAAAACACACCAAAAACATCGGTATCATCAACACCCTGCAGAAATACTACTGCCGTGTTCGTGGTGGTCGTTGCGCTGTTCTGTCTTGCCTGCCGAAAGAAGAACAGATCGGTAAATGCTCTACCCGTGGTCGTAAATGCTGCCGTCGTAAAAAAGAAGCTGCTGCTAAAGCTAAATTCGTTGCTGCTTGGACCCTGAAAGCTGCTGCTGGTCCGGGTCCGGGTTCTAACTTCGTTCTGGCTTCTTCTCTGAAAAAAGTTGGTGGTACCGGTCCGGGTCCGGGTTCTGAAAAAGTTTGGGTTCTGGGTGTTGACCGTGACCAGAACGCTGGTCCGGGTCCGGGTGAAAAAGGTCGTACCATCGCTGCTACCCAGTACGCTGCTGGTGCTGGTCCGGGTCCGGGTACCACCTCTAACCTGTCTGAAGACGCTAAAAAAGCTGTTGAAGACGGTCCGGGTCCGGGTGTTATCGGTGTTGACCGTGACCAGGTTGACGAAGGTAAATACACCGGTCCGGGTCCGGGTAACCCGGAAAAAGGTCGTACCATCGCTGCTACCCAGTACGCTGCTGGTCCGGGTCCGGGTGGTGTTGGTTTCGCTCTGTCTGACTCTGTTAAAAAAGCTGCTAAAGGTCCGGGTCCGGGTGGTAAAAAATCTAACTTCGTTCTGGCTTCTTCTCTGAAAAAAGTTGGTCCGGGTCCGGGTCTGGACGAAGCTGTTTCTAACGACTACAAACTGATCTTCGGTGTTGGTCCGGGTCCGGGTGTTCTGGGTGTTGACCGTGACCAGAAAGCTGAAGGTGAATACACCGGTCCGGGTCCGGGTTACGTTATCATCGACGACCGTATCGAAGGTCAGAAAAACGTTGCTGGTCCGGGTCCGGGTTCTCAGTACGCTACCAACCTGGACGAAGCTGTTTCTAACGACTACGGTCCGGGTCCGGGTGGTGTTGACCTGACCACCACCAACCTGTCTGAAGACGCTAAAAAAGGTCCGGGTCCGGGTAACTACGTTATCATCGACGACGTTATCGAAGGTCAGAAAAACGTTGGTCCGGGTCCGGGTGTTCTGGGTGTTGACCGTGACCAGAACGCTGAAGGTAAATACAAAGCTGCTTACTACGCTGCTGGTGCTGACGTTGTTTACGCTGCTTACAACGAATCTGAATACGCTACCAACCTGGCTGCTTACATCGAATCTGAAGTTATCTCTCGTTTCGCTGCTTACTACGCTGCTGGTGCTGACATCGTTTACGCTGCTTACTACGCTGCTGGTGCTGACGTTATCTACGCTGCTTACTTCTCTCAGGCTGCTACCAACGGTTACGCTGCTTACAAACTGGTTTACGGTGTTGGTTACAAAGCTGCTTACGCTCAGGACAACACCGGTATCAACTACGCTGCTTACAAAATCCTGGACGGTTCTATCACCGTTAAAAAATACGTTATCATCGACGACGAAATCAAAGGTCAGAAAAACGTTGCTTCTAAAAAAGCTGCTATCGGTCTGGCTGCTTGCGGTAACCGTGCTTCTAAATCTGACAAAAAATTCGGTCTGGCTGCTTGCGGTAACCGTGCTTCTAAATCTGACAACAAAAAAAAAGCTGCTTCTTCTGACTACAAACTGGTTTTCGGTATCGGTTTCGCTCTGAAAAAACGTTCTTCTCGTAACGCTGCTTCTTCTTCTGACGTTAAAACCAAAGCTAAAAAAGACGTTGTTTACCAGGCTGCTGGTGGTACCGGTGCTGGTGTTTTCGCTAAAAAAGCTGCTATCGGTCTGGCTGCTTGCGGTAACCGTGCTTCTCGTAAAGACAAAAAAGTTGACGAAGGTAAATACACCTCTAAAGACGGTAAAGAATCTAACTTCAAAAAATACGTTATCATCGACGACCGTATCGAAGGTCAGAAAAACGTTGCTTCTAAAAAAGCTGCTATCGGTCTGGCTGCTTGCGGTAACCGTGCTTCTAAAAAAGACAAAAAAGCTGGTGGTACCGGTGCTGGTGTTTTCTCTGAAGCTAAAGACCTGAACAAAAAAGCTGCTTTCGGTCTGGCTGCTTGCGGTAACCGTGCTTCTCGTTCTGACGAAGCTGCTGCTAAAGCTCCGCCGCACGCTCTGTCTCACCACCACCACCACCACTAATGACGGGTGGCATCCCTGTGACCCCTCCCCAGTGCCTCTCCTGGCCCTGGAAGTTGCCACTCCAGTGCCCACCAGCCTTGTCCTAATAAAATTAAGTTGCATCAAGCT

The cDNA sequence of the designed vaccine 2:

GGACAGATCGCCTGGAGACGCCATCCACGCTGTTTTGACCTCCATAGAAGACACCGGGACCGATCCAGCCTCCGCGGCCGGGAACGGTGCATTGGAACGCGGATTCCCCGTGCCAAGAGTGACTCACCGTCCTTGACACGGCCATGATGGATGGACGCTATGAAACGTGGTCTGTGCTGCGTTCTGCTGCTGTGCGGTGCTGTTTTCGTTTCTCCGTCTATGGCTAAATCTGGTCTGCGTCAGGACCCGCAGTCTACCGCTGCTGCTACCGTTCTGAAACGTGCTGTTGAACTGGACTCTGAATCTCGTTACCCGCAGGCTCTGGTTTGCTACCAGGAAGGTATCGACCTGCTGCTGCAGGTTCTGAAAGGTACCAAAGACAACACCAAACGTTGCAACCTGCGTGAAAAAATCTCTAAATACATGGACCGTGCTGAAAACATCAAAAAATACCTGGACCAGGAAAAAGAAGACGGTAAATACCACAAACAGATCAAAATCGAAGAAAACGCTACCGGTTTCTCTTACGAATCTCTGTTCCGTGAATACCTGAACGAAACCGTTACCGAAGTTTGGATCGAAGACCCGTACATCCGTCACACCCACCAGCTGTACAACTTCCTGCGTTTCTGCGAAATGCTGATCAAACGTCCGTGCAAAGTTAAAACCATCCACCTGCTGACCTCTCTGGACGAAGGTATCGAACAGGTTCAGCAGTCTCGTGGTCTGCAGGAAATCGAAGAATCTCTGCGTTCTCACGGTGTTCTGCTGGAAGTTCAGTACTCTTCTTCTATCCACGACCGTGAAATCCGTTTCAACAACGGTTGGATGATCAAAATCGGTCGTGGTCTGGACTACTTCAAAAAACCGCAGTCTCGTTTCTCTCTGGGTTACTGCGACTTCGACCTGCGTCCGTGCCACGAAACCACCGTTGACATCTTCCACAAAAAACACACCAAAAACATCCGTAAAGACGTTTACGAAGCTGCTGCTAAAGCTAAATTCGTTGCTGCTTGGACCCTGAAAGCTGCTGCTGGTCCGGGTCCGGGTTCTAACTTCGTTCTGGCTTCTTCTCTGAAAAAAGTTGGTGGTACCGGTCCGGGTCCGGGTTCTGAAAAAGTTTGGGTTCTGGGTGTTGACCGTGACCAGAACGCTGGTCCGGGTCCGGGTGAAAAAGGTCGTACCATCGCTGCTACCCAGTACGCTGCTGGTGCTGGTCCGGGTCCGGGTACCACCTCTAACCTGTCTGAAGACGCTAAAAAAGCTGTTGAAGACGGTCCGGGTCCGGGTGTTATCGGTGTTGACCGTGACCAGGTTGACGAAGGTAAATACACCGGTCCGGGTCCGGGTAACCCGGAAAAAGGTCGTACCATCGCTGCTACCCAGTACGCTGCTGGTCCGGGTCCGGGTGGTGTTGGTTTCGCTCTGTCTGACTCTGTTAAAAAAGCTGCTAAAGGTCCGGGTCCGGGTGGTAAAAAATCTAACTTCGTTCTGGCTTCTTCTCTGAAAAAAGTTGGTCCGGGTCCGGGTCTGGACGAAGCTGTTTCTAACGACTACAAACTGATCTTCGGTGTTGGTCCGGGTCCGGGTGTTCTGGGTGTTGACCGTGACCAGAAAGCTGAAGGTGAATACACCGGTCCGGGTCCGGGTTACGTTATCATCGACGACCGTATCGAAGGTCAGAAAAACGTTGCTGGTCCGGGTCCGGGTTCTCAGTACGCTACCAACCTGGACGAAGCTGTTTCTAACGACTACGGTCCGGGTCCGGGTGGTGTTGACCTGACCACCACCAACCTGTCTGAAGACGCTAAAAAAGGTCCGGGTCCGGGTAACTACGTTATCATCGACGACGTTATCGAAGGTCAGAAAAACGTTGGTCCGGGTCCGGGTGTTCTGGGTGTTGACCGTGACCAGAACGCTGAAGGTAAATACAAAGCTGCTTACTACGCTGCTGGTGCTGACGTTGTTTACGCTGCTTACAACGAATCTGAATACGCTACCAACCTGGCTGCTTACATCGAATCTGAAGTTATCTCTCGTTTCGCTGCTTACTACGCTGCTGGTGCTGACATCGTTTACGCTGCTTACTACGCTGCTGGTGCTGACGTTATCTACGCTGCTTACTTCTCTCAGGCTGCTACCAACGGTTACGCTGCTTACAAACTGGTTTACGGTGTTGGTTACAAAGCTGCTTACGCTCAGGACAACACCGGTATCAACTACGCTGCTTACAAAATCCTGGACGGTTCTATCACCGTTAAAAAATACGTTATCATCGACGACGAAATCAAAGGTCAGAAAAACGTTGCTTCTAAAAAAGCTGCTATCGGTCTGGCTGCTTGCGGTAACCGTGCTTCTAAATCTGACAAAAAATTCGGTCTGGCTGCTTGCGGTAACCGTGCTTCTAAATCTGACAACAAAAAAAAAGCTGCTTCTTCTGACTACAAACTGGTTTTCGGTATCGGTTTCGCTCTGAAAAAACGTTCTTCTCGTAACGCTGCTTCTTCTTCTGACGTTAAAACCAAAGCTAAAAAAGACGTTGTTTACCAGGCTGCTGGTGGTACCGGTGCTGGTGTTTTCGCTAAAAAAGCTGCTATCGGTCTGGCTGCTTGCGGTAACCGTGCTTCTCGTAAAGACAAAAAAGTTGACGAAGGTAAATACACCTCTAAAGACGGTAAAGAATCTAACTTCAAAAAATACGTTATCATCGACGACCGTATCGAAGGTCAGAAAAACGTTGCTTCTAAAAAAGCTGCTATCGGTCTGGCTGCTTGCGGTAACCGTGCTTCTAAAAAAGACAAAAAAGCTGGTGGTACCGGTGCTGGTGTTTTCTCTGAAGCTAAAGACCTGAACAAAAAAGCTGCTTTCGGTCTGGCTGCTTGCGGTAACCGTGCTTCTCGTTCTGACGAAGCTGCTGCTAAAGCTCCGCCGCACGCTCTGTCTCACCACCACCACCACCACTAATGACGGGTGGCATCCCTGTGACCCCTCCCCAGTGCCTCTCCTGGCCCTGGAAGTTGCCACTCCAGTGCCCACCAGCCTTGTCCTAATAAAATTAAGTTGCATCAAGCT

# 2. Supplementary Tables and Figures

## 2.1 Supplementary Tables

### 2.1.1 Supplement Table 1

Table S1: Epitopes and their corresponding MHC I molecular alleles predicted by MHCpan4.1 and TepiTool

| Peptide | MHCpan 4.0 MHC I alleles | Tepitool MHC I alleles |
| --- | --- | --- |
| YAAGADVVY | HLA-A*01:01  HLA-A*26:01  HLA-B*15:01 | HLA-C*12:02, HLA-B*46:01, HLA-C*03:02  HLA-B*35:01, HLA-C*02:02, HLA-C*02:09  HLA-C*12:03, HLA-C*16:01, HLA-B*15:02  HLA-B*15:25, HLA-A*25:01, HLA-A*26:01  HLA-B*53:01, HLA-A*01:01, HLA-A*29:02  HLA-B*15:01, HLA-C*03:03, HLA-C*03:04  HLA-C*08:01, HLA-B*35:03, HLA-C*07:01  HLA-B*58:02, HLA-A*30:02 |
| NESEYATNL | HLA-B*39:01  HLA-B*40:01 | HLA-B*18:01, HLA-B*37:01, HLA-B*40:01  HLA-B*40:02, HLA-B*49:01, HLA-B*44:03  HLA-B*44:02, HLA-B*39:01, HLA-B*38:01  HLA-B*14:02 |
| IESEVISRF | HLA-B*40:01 | HLA-B*18:01, HLA-B*44:03, HLA-B*44:02  HLA-B*37:01, HLA-B*13:01, HLA-B*49:01  HLA-B*40:02, HLA-B*40:01, HLA-B*52:01  HLA-B*50:01, HLA-B*38:01 |
| YAAGADIVY | HLA-A*01:01  HLA-A*26:01  HLA-B*15:01 | HLA-C*12:02, HLA-B*46:01, HLA-C*03:02  HLA-B*35:01, HLA-C*02:02, HLA-C*02:09  HLA-C*16:01, HLA-B*15:02, HLA-C*12:03  HLA-B*15:25, HLA-A*01:01, HLA-A*29:02  HLA-A*25:01, HLA-A*26:01, HLA-B*53:01  HLA-B*15:01, HLA-C*03:03, HLA-C*03:04  HLA-C*08:01, HLA-B*35:03, HLA-C*07:01  HLA-B*58:02 |
| YAAGADVIY | HLA-A*01:01  HLA-A*26:01 | HLA-B*35:01, HLA-B*46:01, HLA-C*03:02  HLA-C*12:02, HLA-C*02:02, HLA-C*02:09  HLA-B*15:02, HLA-C*16:01, HLA-C*12:03  HLA-A*01:01, HLA-B*15:25, HLA-A*26:01  HLA-A*29:02, HLA-A*25:01, HLA-B*53:01  HLA-B*15:01, HLA-C*03:03, HLA-C*03:04 |
| FSQAATNGY | HLA-A*01:01  HLA-A*26:01 | HLA-A*01:01, HLA-C*03:02, HLA-C*12:02  HLA-C*02:02, HLA-C*02:09, HLA-A*30:02  HLA-B*15:02, HLA-C*16:01, HLA-B*46:01  HLA-A*29:02, HLA-B*35:01, HLA-A*26:01  HLA-C*12:03, HLA-B*15:25, HLA-B*15:01 |
| KLVYGVGYK | HLA-A*03:01 | HLA-A*03:01 |
| AQDNTGINY | HLA-A*01:01  HLA-B*15:01 | HLA-A*30:02, HLA-B*15:25, HLA-A*01:01  HLA-B*15:01, HLA-A*29:02, HLA-B*15:02  HLA-B*13:01, HLA-C*05:01, HLA-B*44:02 |
| KILDGSITV | HLA-A*02:01 | HLA-A*02:06, HLA-A*02:01, HLA-B*13:02  HLA-C*15:02, HLA-A*32:01, HLA-C*17:01  HLA-B*13:01, HLA-C*07:04, HLA-C*08:01 |

### 2.1.2 Supplement Table 2

Table S2: Epitopes and their corresponding MHC II molecular alleles predicted by MHC-II binding prediction tool and TepiTool

| Peptide | MHCpan 4.0 MHC II alleles | Tepitool MHC II alleles |
| --- | --- | --- |
| SNFVLASSLKKVGGT | HLA-DRB5*01:01 | HLA-DRB1*09:02, HLA-DRB5*01:01, HLA-DRB5*01:02, HLA-DRB5*01:03  HLA-DRB5*01:04, HLA-DRB5*01:05, HLA-DRB5*01:08N, HLA-DRB5*01:11  HLA-DRB5*01:12, HLA-DRB5*01:13, HLA-DRB5*01:14, HLA-DRB5*02:03 |
| SEKVWVLGVDRDQNA | HLA-DRB4*01:01 | HLA-DRB1*04:10, HLA-DRB1*04:67, HLA-DRB4*01:01, HLA-DRB4*01:03  HLA-DRB4*01:04, HLA-DRB4*01:06, HLA-DRB4*01:07, HLA-DRB4*01:08 |
| VLGVDRDQNAEGKYK | HLA-DQA1*03:01/DQB1*03:02  HLA-DRB1*04:01 | HLA-DRB1*04:01, HLA-DRB1*04:13, HLA-DRB1*04:16, HLA-DRB1*04:21  HLA-DRB1*04:22, HLA-DRB1*04:26, HLA-DRB1*04:33, HLA-DRB1*04:38  HLA-DRB1*04:44, HLA-DRB1*04:63, HLA-DRB1*04:64, HLA-DRB1*04:72  HLA-DRB1*04:76 |
| EKGRTIAATQYAAGA | HLA-DPA1*02:01/DPB1*14:01 | HLA-DPA1*02:01, HLA-DPB1*14:01 |
| TTSNLSEDAKKAVED | HLA-DRB1*03:01 | HLA-DRB1*03:01, HLA-DRB1*03:04, HLA-DRB1*03:05, HLA-DRB1*03:06  HLA-DRB1*03:07, HLA-DRB1*03:08, HLA-DRB1*03:10, HLA-DRB1*03:11  HLA-DRB1*03:13, HLA-DRB1*03:14, HLA-DRB1*03:15, HLA-DRB1*03:18  HLA-DRB1*03:19, HLA-DRB1*03:20, HLA-DRB1*03:21, HLA-DRB1*03:22  HLA-DRB1*03:23, HLA-DRB1*03:24, HLA-DRB1*03:25, HLA-DRB1*03:26  HLA-DRB1*03:28, HLA-DRB1*03:30, HLA-DRB1*03:31, HLA-DRB1*03:32  HLA-DRB1*03:33, HLA-DRB1*03:34, HLA-DRB1*03:36, HLA-DRB1*03:37  HLA-DRB1*03:39, HLA-DRB1*03:40, HLA-DRB1*03:43, HLA-DRB1*03:44  HLA-DRB1*03:45, HLA-DRB1*03:46, HLA-DRB1*03:47, HLA-DRB1*03:48  HLA-DRB1*03:49, HLA-DRB1*03:50, HLA-DRB1*03:51, HLA-DRB1*03:52  HLA-DRB1*03:54, HLA-DRB1*03:55, HLA-DRB1*11:07, HLA-DRB1*14:76  HLA-DRB1*14:79 |
| VIGVDRDQVDEGKYT | HLA-DRB4*01:01 | HLA-DRB4*01:01, HLA-DRB4*01:03, HLA-DRB4*01:04, HLA-DRB4*01:06  HLA-DRB4*01:07, HLA-DRB4*01:08, HLA-DRB1*03:01, HLA-DRB1*03:03  HLA-DRB1*03:04, HLA-DRB1*03:06, HLA-DRB1*03:07, HLA-DRB1*03:08  HLA-DRB1*03:11, HLA-DRB1*03:13, HLA-DRB1*03:14, HLA-DRB1*03:15  HLA-DRB1*03:18, HLA-DRB1*03:19, HLA-DRB1*03:20, HLA-DRB1*03:21  HLA-DRB1*03:22, HLA-DRB1*03:23, HLA-DRB1*03:24, HLA-DRB1*03:25  HLA-DRB1*03:26, HLA-DRB1*03:28, HLA-DRB1*03:30, HLA-DRB1*03:31  HLA-DRB1*03:32, HLA-DRB1*03:33, HLA-DRB1*03:34, HLA-DRB1*03:36  HLA-DRB1*03:37, HLA-DRB1*03:39, HLA-DRB1*03:43, HLA-DRB1*03:44  HLA-DRB1*03:45, HLA-DRB1*03:46, HLA-DRB1*03:47, HLA-DRB1*03:48  HLA-DRB1*03:49, HLA-DRB1*03:50, HLA-DRB1*03:51, HLA-DRB1*03:52  HLA-DRB1*03:54, HLA-DRB1*03:55, HLA-DRB1*11:07 |
| NPEKGRTIAATQYAA | HLA-DPA1*02:01/DPB1*14:01 | HLA-DPA1*02:01, HLA-DPB1*14:01 |
| GVGFALSDSVKKAAK | HLA-DRB5*01:01 | HLA-DRB1*03:02, HLA-DRB1*03:53, HLA-DRB1*09:02, HLA-DRB1*09:08  HLA-DRB1*14:19, HLA-DRB1*14:46, HLA-DRB1*14:51, HLA-DRB5*01:01  HLA-DRB5*01:02, HLA-DRB5*01:03, HLA-DRB5*01:04, HLA-DRB5*01:05  HLA-DRB5*01:08N, HLA-DRB5*01:11, HLA-DRB5*01:12, HLA-DRB5*01:13  HLA-DRB5*01:14, HLA-DRB5*02:03 |
| GKKSNFVLASSLKKV | HLA-DRB5*01:01 | HLA-DRB1*09:02, HLA-DRB1*14:46, HLA-DRB5*01:01, HLA-DRB5*01:02  HLA-DRB5*01:03, HLA-DRB5*01:04, HLA-DRB5*01:05, HLA-DRB5*01:08N  HLA-DRB5*01:11, HLA-DRB5*01:12, HLA-DRB5*01:13, HLA-DRB5*01:14  HLA-DRB5*02:03 |
| LDEAVSNDYKLIFGV | HLA-DRB1*03:01 | HLA-DRB1*03:01, HLA-DRB1*03:04, HLA-DRB1*03:05, HLA-DRB1*03:06  HLA-DRB1*03:07, HLA-DRB1*03:08, HLA-DRB1*03:10, HLA-DRB1*03:11  HLA-DRB1*03:13, HLA-DRB1*03:14, HLA-DRB1*03:15, HLA-DRB1*03:18  HLA-DRB1*03:19, HLA-DRB1*03:20, HLA-DRB1*03:21, HLA-DRB1*03:22  HLA-DRB1*03:23, HLA-DRB1*03:24, HLA-DRB1*03:25, HLA-DRB1*03:26  HLA-DRB1*03:28, HLA-DRB1*03:30, HLA-DRB1*03:31, HLA-DRB1*03:32  HLA-DRB1*03:33, HLA-DRB1*03:34, HLA-DRB1*03:36, HLA-DRB1*03:37  HLA-DRB1*03:39, HLA-DRB1*03:43, HLA-DRB1*03:44, HLA-DRB1*03:45  HLA-DRB1*03:46, HLA-DRB1*03:47, HLA-DRB1*03:48, HLA-DRB1*03:49  HLA-DRB1*03:50, HLA-DRB1*03:51, HLA-DRB1*03:52, HLA-DRB1*03:54  HLA-DRB1*03:55, HLA-DRB1*11:07, HLA-DRB1*14:76, HLA-DRB1*14:79 |
| VLGVDRDQKAEGEYT | HLA-DRB1*03:01 | HLA-DRB1*03:01 |
| YVIIDDRIEGQKNVA | HLA-DRB1*03:01 | HLA-DRB1*03:01, HLA-DRB1*03:04, HLA-DRB1*03:06, HLA-DRB1*03:07  HLA-DRB1*03:08, HLA-DRB1*03:11, HLA-DRB1*03:13, HLA-DRB1*03:15  HLA-DRB1*03:18, HLA-DRB1*03:19, HLA-DRB1*03:20, HLA-DRB1*03:21  HLA-DRB1*03:22, HLA-DRB1*03:23, HLA-DRB1*03:25, HLA-DRB1*03:26  HLA-DRB1*03:28, HLA-DRB1*03:30, HLA-DRB1*03:31, HLA-DRB1*03:32  HLA-DRB1*03:33, HLA-DRB1*03:34, HLA-DRB1*03:36, HLA-DRB1*03:37  HLA-DRB1*03:39, HLA-DRB1*03:43, HLA-DRB1*03:44, HLA-DRB1*03:45  HLA-DRB1*03:46, HLA-DRB1*03:47, HLA-DRB1*03:48, HLA-DRB1*03:49  HLA-DRB1*03:50, HLA-DRB1*03:51, HLA-DRB1*03:52, HLA-DRB1*03:54  HLA-DRB1*03:55, HLA-DRB1*11:07 |
| SQYATNLDEAVSNDY | HLA-DRB3*02:02 | HLA-DRB3*02:02, HLA-DRB3*02:09, HLA-DRB3*02:10, HLA-DRB3*02:12  HLA-DRB3*02:13, HLA-DRB3*02:16, HLA-DRB3*02:18, HLA-DRB3*02:19  HLA-DRB3*02:20, HLA-DRB3*02:21, HLA-DRB3*02:22, HLA-DRB3*02:23  HLA-DRB3*02:25 |
| GVDLTTTNLSEDAKK | HLA-DQA1*03:01/DQB1*03:02 | HLA-DQA1*03:01, HLA-DQB1*03:02 |
| NYVIIDDVIEGQKNV | HLA-DRB1*03:01  HLA-DRB1*04:05 | HLA-DRB1*03:01, HLA-DRB1*03:04, HLA-DRB1*03:06, HLA-DRB1*03:08  HLA-DRB1*03:11, HLA-DRB1*03:13, HLA-DRB1*03:15, HLA-DRB1*03:18  HLA-DRB1*03:19, HLA-DRB1*03:20, HLA-DRB1*03:22, HLA-DRB1*03:23  HLA-DRB1*03:25, HLA-DRB1*03:26, HLA-DRB1*03:28, HLA-DRB1*03:30  HLA-DRB1*03:31, HLA-DRB1*03:32, HLA-DRB1*03:33, HLA-DRB1*03:34  HLA-DRB1*03:36, HLA-DRB1*03:37, HLA-DRB1*03:39, HLA-DRB1*03:42  HLA-DRB1*03:43, HLA-DRB1*03:44, HLA-DRB1*03:45, HLA-DRB1*03:46  HLA-DRB1*03:47, HLA-DRB1*03:48, HLA-DRB1*03:50, HLA-DRB1*03:51  HLA-DRB1*03:52, HLA-DRB1*03:54, HLA-DRB1*03:55, HLA-DRB1*04:05  HLA-DRB1*04:13, HLA-DRB1*04:22, HLA-DRB1*04:29, HLA-DRB1*04:30  HLA-DRB1*04:45, HLA-DRB1*04:48, HLA-DRB1*04:57, HLA-DRB1*04:72  HLA-DRB1*04:77, HLA-DRB1*04:83, HLA-DRB1*04:84, HLA-DRB1*04:87  HLA-DRB1*04:89, HLA-DRB4*01:04 |

### 2.1.3 Supplement Table 3

Table S3: Docking studies of T-cell epitopes with MHC I allele structures.

|  | Peptide | MHC alleles | PDB id | Chain | ΔG (kcal mol-1) | Kd (M) at ℃ | Hydrogen bond | Salt bridges | Representative | Weighted Score |
| --- | --- | --- | --- | --- | --- | --- | --- | --- | --- | --- |
| CTL epitopes | AQDNTGINY | HLA-A*01:01 | 4nqx | A | -10.4 | 2.40E-08 | Arg 156-Asp 3、Arg 156-Gln 2、Val 150-Asp 3、Arg 114-Tyr 9、Asp 74-Tyr 9、Lys 146-Gln 2、Arg 163-Asn 8 | NA | Center | -535.7 |
|  |  |  |  |  |  |  |  |  | Lowest Energy | -668.7 |
|  | FSQAATNGY | HLA-A*01:01 | 4nqx | A | -8 | 1.30E-06 | Trp 147-Ser 2、Arg 114-Gln 3、Arg 156-Ala 4（2）、Arg 156-Tyr 9、Thr 73-Thr 6、Arg 163-Tyr 9（2）、Glu 63-Tyr 9 | NA | Center | -533.9 |
|  |  |  |  |  |  |  |  |  | Lowest Energy | -624 |
|  | IESEVISRF | HLA-B*40:01 | NA | A | -8.7 | 4.40E-07 | Lys 170-Glu 2、Ser 101-Val 5、Tyr 140-Arg 8、Tyr 140-Phe 9、Arg 121-Ser 7、Arg 121-Phe 9 | NA | Center | -592.3 |
|  |  |  |  |  |  |  |  |  | Lowest Energy | -678.1 |
|  | KILDGSITV | HLA-A*02:01 | 4u6y | A | -9.2 | 1.90E-07 | Arg 97-Lys 1、Tyr 116-Lys 1、Thr 73-Lys 1、Thr 73-Asp 4、Tyr 99-Val 9、Lys 66-Thr 8 | NA | Center | -630 |
|  |  |  |  |  |  |  |  |  | Lowest Energy | -668.4 |
|  | KLVYGVGYK | HLA-A*03:01 | 7l1c | A | -10.4 | 2.30E-08 | Glu 63-Val 3、Tyr 99-Leu 2、Tyr 99-Lys 1、Gln 70-Lys 1、Gln 70-Leu 2、Glu 152-Lys 1、Trp 167-Val 6、Glu 166-Tyr 8、Gln 62-Val 6、Gln 62-Gly 7、Asn 66-Lys 9 | NA | Center | -599.7 |
|  |  |  |  |  |  |  |  |  | Lowest Energy | -671.3 |
|  | NESEYATNL | HLA-B*39:01 | NA | A | -7.7 | 2.10E-06 | Trp 191-Asn 1、Arg 86-Glu 2（2)、Thr 187-Glu 4、Thr 187-Asn 8、Asn 94-Leu 9、Tyr 31-Tyr 5、Tyr 83-Tyr 5、Tyr 195-Tyr 5 | NA | Center | -568.5 |
|  |  |  |  |  |  |  |  |  | Lowest Energy | -615.3 |
|  | YAAGADIVY | HLA-A*01:01 | 4nqx | A | -9.1 | 2.10E-07 | Asn 66-Tyr 1、Arg 163-Val 8、Trp 147-Tyr 9、Arg 156-Gly 4、Arg 156-Ala 5、Arg 156-Asp 6、Arg 156-Ile 7、Gln 155-Ala 5 | NA | Center | -646.9 |
|  |  |  |  |  |  |  |  |  | Lowest Energy | -663.8 |
|  | YAAGADVIY | HLA-A*01:01 | 4nqx | A | -9.8 | 6.30E-08 | Tyr 84-Tyr 1、Thr 143-Tyr 1、Lys 146-Tyr 1、Thr 73-Ala 2、Asn 77-Tyr 1、Asn 77-Tyr 9、Arg 156-Asp 6（2） | NA | Center | -625.1 |
|  |  |  |  |  |  |  |  |  | Lowest Energy | -761.4 |
|  | YAAGADVVY | HLA-A*01:01 | 4nqx | A | -10.1 | 3.90E-08 | His 70-Gly 4、Asp 74-His 70、Tyr 99-Ala 2、Arg 163-Tyr 1（2）、Arg 156-Ala 3、Arg 156-Gly 4、Arg 156-Val 8 | NA | Center | -593.3 |
|  |  |  |  |  |  |  |  |  | Lowest Energy | -707.9 |

### 2.1.4 Supplement Table 4

Table 4: Docking studies of T-cell epitopes with MHC II allele structures.

|  | Peptide | MHC alleles | PDB id | Chain | ΔG (kcal mol-1) | Kd (M) at ℃ | Hydrogen bond | Salt bridges | Representative | Weighted Score |
| --- | --- | --- | --- | --- | --- | --- | --- | --- | --- | --- |
| HTL epitopes | EKGRTIAATQYAAGA | HLA-DPA1*02:01/DPB1*14:01 | NA | A | -9 | 2.40E-07 | Asn93-Ala15、Tyr40-Tyr11、Ala82-Arg4（2）、Ser84-Thr9 | NA | Center | -535.7 |
|  |  |  |  | B |  |  | Glu111-Lys2、Arg104-Gly14 | Glu114-Lys2 | Lowest Energy | -668.7 |
|  | GKKSNFVLASSLKKV | HLA-DRB5*01:01 | NA | A | -9.3 | 1.40E-07 | Glu53-Ser11、Asn67-Lys2 | Glu53-Lys14 | Center | -618.6 |
|  |  |  |  | B |  |  | Asp99-Asn5(3)、Arg100-Asn5、Tyr42-Gly1 | Asp95-Lys3 | Lowest Energy | -728 |
|  | GVDLTTTNLSEDAKK | HLA-DQA1*03:01/DQB1*03:02 | NA | A | -8.1 | 1.10E-06 | Arg79-Val2、Asp81-Leu4 | Arg79-Asp3 | Center | -514.6 |
|  |  |  |  | B |  |  | Thr109-Ser10 | NA | Lowest Energy | -630.6 |
|  | GVGFALSDSVKKAAK | HLA-DRB5*01:01 | NA | A | -9 | 2.40E-07 | Ser51-Ala13(2) | NA | Center | -634.7 |
|  |  |  |  | B |  |  | Thr106-Leu6、Asn111-Val2 | NA | Lowest Energy | -735.9 |
|  | LDEAVSNDYKLIFGV | HLA-DRB1*03:01 | NA | A | -9.3 | 1.50E-07 | Gln7-Tyr9、Glu53-Asp2、Ser51-Leu1 | NA | Center | -597.6 |
|  |  |  |  | B |  |  | Gln99-Leu11、Arg103-Asp8（3） | Arg103-Asp8 | Lowest Energy | -712.3 |
|  | NPEKGRTIAATQYAA | HLA-DPA1*02:01/DPB1*14:01 | NA | A | -10 | 4.50E-08 | Ser38-Tyr13、Glu86-Thr11 | NA | Center | -580.6 |
|  |  |  |  | B |  |  | His108-Lys4、Asn109-Arg6、Arg104-Gln12、Gln42-Ala14、Lys98-Ala15 | Glu111-Lys4 | Lowest Energy | -748.3 |
|  | NYVIIDDVIEGQKNV | HLA-DRB1*03:01 | NA | A | -7.4 | 3.80E-06 | Glu53-Asn14、Thr39-Val15、Asn60-Asn1 | NA | Center | -585.8 |
|  |  |  |  | B |  |  | Gln99-Asn1、Arg103-Asn1（3）、Asn111-Tyr2、His110-Ile5 | His110-Asp6 | Lowest Energy | -720 |
|  | SEKVWVLGVDRDQNA | HLA-DRB4*01:01 | NA | A | -8.5 | 5.50E-07 | Gln55-Ser1、Gln7-Val9 | NA | Center | -601 |
|  |  |  |  | B |  |  | Asn111-Leu7、Arg99-Glu2（2）、Arg99-Arg11、Arg99-Asn14、Thr106-Asn14 | Arg99-Glu2 | Lowest Energy | -722.3 |
|  | SNFVLASSLKKVGGT | HLA-DRB5*01:01 | NA | A | -9 | 2.40E-07 | Glu9-Lys10、Gly56-Ser7、Asn67-Thr15、Asn67-Gly14 | Glu9-Lys10、Asp64-Lys10 | Center | -559.5 |
|  |  |  |  | B |  |  | Trp90-Thr15、Trp90-Gly13、Tyr89-Gly13、Asn111-Asn2 | Asp40-Lys10 | Lowest Energy | -676.5 |
|  | SQYATNLDEAVSNDY | HLA-DRB3*02:02 | NA | A | -8.3 | 8.30E-07 | Ser51-Asn13、Asn60-Tyr3 | NA | Center | -491.8 |
|  |  |  |  | B |  |  | Asn111-Tyr15、His110-Ser12、Gln99-Asp8、Gln99-Thr5 | NA | Lowest Energy | -589 |
|  | TTSNLSEDAKKAVED | HLA-DRB1*03:01 | NA | A | -8.3 | 7.50E-07 | Ser51-Thr2（2）、Ser51-Leu5、Glu53-Thr1、 | NA | Center | -410.7 |
|  |  |  |  | B |  |  | Gln99-Glu14、Lys100-Asp15、Arg103-Asp15（2）、Arg103-Ala12、Arg103-Lys11（2）、Asn106-Lys11、His110-Asn4 | Lys100-Asp15、Arg103-Asp15、His110-Asp8 | Lowest Energy | -471.5 |
|  | VIGVDRDQVDEGKYT | HLA-DRB4*01:01 | NA | A | -10 | 4.70E-08 | Ser51-Gly3、Gln55-Asp5、Asn60-Arg6、Glu9-Arg6（2） | Glu9-Arg6 | Center | -636.1 |
|  |  |  |  | B |  |  | Arg100-Asp10（2）、Asn111-Val1、Asn111-Thr15、Tyr110-Tyr14、Thr106-Thr15、Thr106-Gly12、Arg99-Glu11 | Arg100-Asp10、Arg99-Glu11 | Lowest Energy | -698 |
|  | VLGVDRDQKAEGEYT | HLA-DRB1*04:01 | NA | A | -9.4 | 1.20E-07 | Arg48-Thr15、Glu53-Gln8、Ser51-Glu11 | NA | Center | -637.4 |
|  |  |  |  | B |  |  | Asn111-Leu2 | NA | Lowest Energy | -694.4 |
|  | VLGVDRDQNAEGKYK | HLA-DRB1*04:01 | NA | A | -8.6 | 4.70E-07 | Asn62-Tyr14、Glu55-Tyr14、Glu55-Asn9、Ser53-Asn9 | NA | Center | -613.2 |
|  |  |  |  | B |  |  | Asn82-Asp5、Asn82-Gly3、Asn82-Val4、His81-Arg6（2） | NA | Lowest Energy | -690.2 |
|  | YVIIDDRIEGQKNVA- | HLA-DRB1*03:01 | NA | A | -7.7 | 2.20E-06 | Ile5-Tyr1、Glu53-Ile3、Glu53-Lys12 | Glu53-Lys12 | Center | -568.3 |
|  |  |  |  | B |  |  | Ser42-Tyr1、Asn111-Val2、Asn111-Ala15、His110-Val14 | NA | Lowest Energy | -646.2 |

### 2.1.5 Supplement Table 5

Table S5: The quality validation of the MHC allele molecules

| Swiss model of HLA molecules | ERRAT Score | Ramachandran Plot | | | | |
| --- | --- | --- | --- | --- | --- | --- |
|  |  | Residues in most favoured regions | Residues in additional allowed regions | Residues in generously allowed regions | Residues in disallowed regions | Number of non-glycine and non-proline residues |
| HLA-B*39:01 | 97.0037 | 93.30% | 5.80% | 0.80% | 0.00% | 100.00% |
| HLA-B*40:01 | 97.0037 | 93.80% | 5.40% | 0.80% | 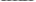0.00% | 100.00% |
| HLA-DPA1*02:01/DPB1*14:01DPA10201ADPB11401 | 95.2978 | 93.80% | 5.90% | 0.00% | 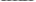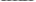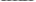0.30% | 100.00% |
| HLA-DQA1*03:01/DQB1*03:02 | 89.3064 | 89.40% | 9.40% | 0.90% | 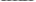0.30% | 100.00% |
| HLA-DRB1*03:01 | 90.2077 | 94.40% | 5.30% | 0.00% | 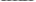0.30% | 100.00% |
| HLA-DRB3*02:02 | 89.1566 | 94.40% | 5.30% | 0.00% | 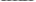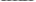0.30% | 100.00% |
| HLA-DRB4*01:01 | 89.6142 | 93.80% | 5.80% | 0.00% | 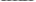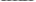0.30% | 100.00% |
| HLA-DRB5*01:01 | 90.3614 | 93.50% | 6.20% | 0.00% | 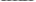0.30% | 100.00% |

### 2.1.7 Supplement Table 7

Table S7: The comparison of two vaccines in Alphafold and Chai-1 construction

|  | Alphafold | | Chai-1 | |
| --- | --- | --- | --- | --- |
|  | V1 | V2 | V1 | V2 |
| ERRAT Score | 81.818 | 75.798 | 83.522 | 90.783 |
| Z-Score | -4.48 | -4.95 | -6.15 | -5.42 |
| Ramachandran Plot |  |  |  |  |
| Residues in most favoured regions | 87.2% | 90.6% | 89.6% | 88.6% |
| Residues in additional allowed regions | 10.5% | 8.8% | 9.8% | 10.70% |
| Residues in generously allowed regions | 0.9% | 0.00% | 0.20% | 0.20% |
| Residues in disallowed regions | 1.4% | 0.06% | 0.40% | 0.60% |
| Number of non-glycine and non-proline residues | 100.00% | 100.00% | 100.00% | 100.00% |
| Verified 3D score | 82.84% | 80.75% | 89.65% | 89.62% |

### 2.1.8 Supplement Table 8

Table S8: Predicted discontinuous B-cell epitope residues of the designed vaccine 1 construct

| Number | Residues | Number of residues | Score |
| --- | --- | --- | --- |
| 1 | A:G1, A:I2, A:I3, A:N4, A:T5, A:L6, A:Q7, A:K8, A:Y9, A:Y10, A:C11, A:R12, A:V13, A:R14, A:G15, A:G16, A:R17, A:C18, A:A19, A:V20, A:L21, A:S22, A:C23, A:L24, A:P25, A:K26, A:E27, A:E28, A:Q29, A:I30, A:G31, A:K32, A:C33, A:S34, A:T35, A:R36, A:G37, A:R38, A:K39, A:C40, A:C41, A:R42, A:R43, A:K44, A:K45, A:E46, A:A47, A:A48, A:A49, A:K50, A:A51, A:K52, A:F53, A:V54, A:A55, A:A56, A:W57, A:T58, A:L59, A:K60, A:A61, A:A62, A:A63, A:G64, A:P65, A:G66, A:P67, A:G68, A:S69, A:N70, A:F71, A:V72, A:L73, A:A74, A:S75, A:S76, A:L77, A:K78, A:K79, A:V80, A:G81, A:G82, A:T83, A:G84, A:P85, A:G86, A:P87, A:G88, A:S89, A:K91, A:V92, A:W93, A:V94, A:L95, A:G96, A:V97, A:D98, A:R99, A:D100, A:Q101, A:N102, A:A103, A:G104, A:P105, A:G106, A:P107, A:K110, A:G111, A:R112, A:T113, A:I114, A:A115, A:A116, A:T117, A:Q118, A:Y119, A:A120, A:A121, A:G122, A:A123, A:G124, A:P125, A:S131, A:N132, A:L133, A:S134, A:E135, A:D136, A:A137, A:K138, A:K139, A:A140, A:V141, A:E142, A:D143, A:G144 | 136 | 0.788 |
| 2 | A:A519, A:S520, A:K521, A:S522, A:D523, A:N524, A:K525, A:K526, A:N591, A:R592, A:A593, A:S594, A:R595, A:K596, A:D597, A:R646, A:A647, A:S648, A:K649, A:K650, A:D651, A:K652, A:K653, A:A654, A:G655, A:G656, A:T657, A:G658 | 28 | 0.781 |
| 3 | A:R154, A:D155, A:Q156, A:V157, A:D158, A:E159, A:G160, A:K161, A:Y162, A:T163, A:I176, A:A177, A:A178, A:T179, A:Q180, A:Y181, A:A182, A:A183, A:G184, A:A193, A:L194, A:S195, A:D196, A:S197, A:V198, A:K199, A:K200, A:A201, A:A202, A:K203, A:G204, A:S219, A:L220, A:K221, A:K222, A:G242, A:V243, A:G244, A:Y262, A:T263, A:G264, A:N281, A:V282, A:A283, A:G284, A:P285, A:V299, A:S300, A:N301, A:D302, A:Y303, A:G304, A:P305, A:T315, A:N316, A:L317, A:S318, A:E319, A:D320, A:A321, A:K322, A:K323, A:G324, A:P325, A:V336, A:I337, A:E338, A:G339, A:Q340, A:K341, A:N342, A:V343, A:G344, A:P345, A:G346, A:P347, A:G348, A:V349, A:G351, A:D353, A:D355, A:Q356, A:N357, A:A358, A:E359, A:G360, A:K361, A:Y362, A:Y366, A:E382, A:Y383, A:A384, A:A389, A:A400, A:A401, A:Y403, A:A404, A:A405, A:G406, A:A407, A:D408, A:I409, A:V410, A:Y411, A:A412, A:A413, A:Y414, A:Y415, A:A416, A:A417, A:G418, A:A419, A:D420, A:V421, A:K447, A:A448, A:A449, A:Y450, A:A451, A:Q452, A:D453, A:N454, A:T455, A:G456, A:I457, A:N458, A:Y459, A:A460, A:A461, A:Y462, A:K463, A:I464, A:L465, A:D466, A:G467, A:S468, A:T470, A:V471, A:K472, A:K473, A:Y474, A:R502, A:A503, A:S504, A:K505, A:S506, A:D507, A:R546, A:S547, A:S548, A:R549, A:N550, A:A551, A:A552, A:S553, A:S554, A:S555, A:D556, A:V557, A:K558, A:T559, A:K560, A:A561, A:K562, A:K563, A:D564, A:V565, A:T606, A:S607, A:K608, A:D609, A:G610, A:K611, A:A683, A:S684, A:S686, A:D687, A:E688, A:A689, A:A690, A:A691, A:K692, A:A693, A:P694, A:P695, A:H696, A:A697, A:L698, A:S699, A:H700, A:H701, A:H702, A:H703, A:H704, A:H705 | 195 | 0.649 |
| 4 | A:A431, A:T432, A:N433, A:G434, A:Y435, A:K527, A:A528, A:A529, A:K599, A:V600, A:D601, A:E602, A:G603, A:K604, A:E612 | 15 | 0.643 |
| 5 | A:A258, A:E259, A:G260, A:E261, A:I276, A:E277, A:G278, A:K280 | 8 | 0.606 |
| 6 | A:G151, A:V152, A:D153 | 3 | 0.518 |

### 2.1.9 Supplement Table 9

Table S9: Predicted discontinuous B-cell epitope residues of the designed vaccine 2 construct

| Number | Residues | Number of residues | Score |
| --- | --- | --- | --- |
| 1 | A:R1, A:D3, A:V4, A:Y5, A:E6, A:A7, A:A8, A:A9, A:K10, A:A11, A:K12, A:F13, A:V14, A:A15, A:A16, A:W17, A:T18, A:L19, A:K20, A:A21, A:A22, A:A23, A:G24, A:P25, A:G26, A:P27, A:G28, A:S29, A:N30, A:F31, A:V32, A:L33, A:A34, A:S35, A:S36, A:L37, A:K38, A:K39, A:V40, A:G41, A:G42, A:T43, A:G44, A:P45, A:G46, A:P47, A:G48, A:S49, A:E50, A:K51, A:V52, A:W53, A:V54, A:L55, A:G56, A:V57, A:D58, A:D60, A:Q61, A:N62, A:A63, A:G64, A:E69, A:K70, A:G71, A:R72, A:T73, A:I74, A:A75, A:A76, A:T77, A:Q78, A:Y79, A:A80, A:A81, A:G82, A:A83, A:G84, A:G88, A:T89, A:T90, A:S91, A:N92, A:L93, A:S94, A:E95, A:D96, A:A97, A:K98, A:K99, A:A100, A:V101, A:E102, A:I110, A:G111, A:V112, A:D113, A:R114, A:D115, A:Q116, A:V117, A:D118, A:E119, A:G120, A:K121, A:Y122, A:T123, A:G133, A:R134, A:T135, A:I136, A:A137, A:A138, A:T139, A:Q140, A:Y141, A:A142, A:A143, A:G148, A:G149, A:V150, A:G151, A:F152, A:A153, A:L154, A:S155, A:D156, A:S157, A:V158, A:K159, A:K160, A:A161, A:A162, A:K163, A:G166, A:S172, A:N173, A:F174, A:V175, A:L176, A:A177, A:S178, A:S179, A:L180, A:K181, A:K182, A:V183, A:G184, A:A192, A:V193, A:S194, A:N195, A:D196, A:Y197, A:K198, A:L199, A:I200, A:F201, A:G202, A:V203, A:G204, A:P205, A:V212, A:D213, A:R214, A:D215, A:Q216, A:K217, A:A218, A:E219, A:G220, A:E221, A:Y222, A:T223, A:G224, A:P225, A:P227, A:D233, A:D234, A:R235, A:I236, A:E237, A:G238, A:Q239, A:K240, A:N241, A:V242, A:A243, A:G244, A:P245, A:T253, A:N254, A:L255, A:D256, A:E257, A:A258, A:V259, A:S260, A:N261, A:D262, A:Y263, A:G264, A:T274, A:T275, A:N276, A:L277, A:S278, A:E279, A:D280, A:A281, A:K282, A:K283, A:G284, A:D294, A:D295, A:V296, A:I297, A:E298, A:G299, A:Q300, A:N302, A:V303, A:G304, A:A391, A:T392, A:N393, A:G394, A:Y395 | 228 | 0.7 |
| 2 | A:R606, A:A607, A:S608, A:K609, A:K610, A:D611, A:A614, A:G615, A:R642, A:A643, A:S644, A:R645, A:S646, A:D647 | 14 | 0.68 |
| 3 | A:N317, A:A318, A:E319, A:G320, A:K321, A:Y322, A:K323, A:A324, A:S341, A:E342, A:Y343, A:A344, A:T345, A:N346, A:E440, A:I441, A:K442, A:G443, A:Q444, A:K445, A:N446, A:V447, A:A448, A:S449, A:K450, A:K451, A:A458, A:C459, A:G460, A:N461, A:R462, A:A463, A:S464, A:K465, A:S466, A:D467, A:K468, A:K469, A:F470, A:G471, A:L472, A:A473, A:A474, A:C475, A:G476, A:N477, A:R478, A:A479, A:S480, A:K481, A:S482, A:D483, A:N484, A:K485, A:K486, A:K487, A:A488, A:A489, A:S490, A:S491, A:L503, A:K504, A:K505, A:R506, A:S507, A:S508, A:R509, A:N510, A:A511, A:A512, A:S513, A:S514, A:S515, A:D516, A:V517, A:K518, A:T519, A:K520, A:A521, A:K522, A:K523, A:V526, A:A529, A:A530, A:G531, A:G532, A:T533, A:G534, A:A535, A:G536, A:V537, A:F538, A:A539, A:K540, A:K541, A:A542, A:A547, A:A548, A:C549, A:G550, A:N551, A:R552, A:A553, A:S554, A:R555, A:K556, A:D557, A:K558, A:K559, A:V560, A:E562, A:G563, A:K564, A:Y565, A:T566, A:S567, A:K568, A:D569, A:G570, A:K571, A:E572, A:S573, A:N574, A:R584, A:I585, A:E586, A:G587, A:Q588, A:N590, A:V591, A:A592, A:S593, A:K594, A:K595, A:A602, A:C603, A:G604, A:N605, A:E624, A:D627, A:L628, A:N629, A:K630, A:K631, A:A632, A:C639, A:G640, A:N641 | 148 | 0.654 |
| 4 | A:G168, A:G169, A:K171 | 3 | 0.5 |

### 2.1.10 Supplement Table 10

Table S10: Predicted Linear Epitope of Vaccine 1

| Number | Chain | Start | End | Peptide | Number of residues | Score |
| --- | --- | --- | --- | --- | --- | --- |
| 1 | A | 1 | 88 | GIINTLQKYYCRVRGGRCAVLSCLPKEEQIGKCSTRGRKCCRRKKEAAAKAKFVAAWTLKAAAGPGPGSNFVLASSLKKVGGTGPGPG | 88 | 0.866 |
| 2 | A | 518 | 529 | RASKSDNKKKAA | 12 | 0.744 |
| 3 | A | 682 | 705 | RASRSDEAAAKAPPHALSHHHHHH | 24 | 0.74 |
| 4 | A | 447 | 472 | KAAYAQDNTGINYAAYKILDGSITVK | 26 | 0.717 |
| 5 | A | 645 | 657 | NRASKKDKKAGGT | 13 | 0.715 |
| 6 | A | 151 | 163 | GVDRDQVDEGKYT | 13 | 0.709 |
| 7 | A | 400 | 421 | AAYYAAGADIVYAAYYAAGADV | 22 | 0.707 |
| 8 | A | 131 | 144 | SNLSEDAKKAVEDG | 14 | 0.689 |
| 9 | A | 591 | 612 | NRASRKDKKVDEGKYTSKDGKE | 22 | 0.688 |
| 10 | A | 193 | 204 | ALSDSVKKAAKG | 12 | 0.685 |
| 11 | A | 431 | 435 | ATNGY | 5 | 0.678 |
| 12 | A | 90 | 106 | EKVWVLGVDRDQNAGPG | 17 | 0.672 |
| 13 | A | 315 | 328 | TNLSEDAKKGPGPG | 14 | 0.648 |
| 14 | A | 299 | 305 | VSNDYGP | 7 | 0.634 |
| 15 | A | 354 | 364 | RDQNAEGKYKA | 11 | 0.628 |
| 16 | A | 336 | 348 | VIEGQKNVGPGPG | 13 | 0.604 |
| 17 | A | 110 | 124 | KGRTIAATQYAAGAG | 15 | 0.595 |
| 18 | A | 544 | 566 | KKRSSRNAASSSDVKTKAKKDVV | 23 | 0.58 |
| 19 | A | 176 | 184 | IAATQYAAG | 9 | 0.575 |
| 20 | A | 258 | 264 | AEGEYTG | 7 | 0.575 |
| 21 | A | 276 | 284 | IEGQKNVAG | 9 | 0.565 |
| 22 | A | 220 | 223 | LKKV | 4 | 0.537 |

### 2.1.11 Supplement Table 11

Table S11: Predicted Linear Epitope of Vaccine 2

| Number | Chain | Start | End | Peptide | Number of residues | Score |
| --- | --- | --- | --- | --- | --- | --- |
| 1 | A | 1 | 41 | RKDVYEAAAKAKFVAAWTLKAAAGPGPGSNFVLASSLKKVG | 41 | 0.862 |
| 2 | A | 212 | 225 | VDRDQKAEGEYTGP | 14 | 0.853 |
| 3 | A | 233 | 245 | DDRIEGQKNVAGP | 13 | 0.816 |
| 4 | A | 503 | 523 | LKKRSSRNAASSSDVKTKAKK | 21 | 0.792 |
| 5 | A | 473 | 491 | AACGNRASKSDNKKKAASS | 19 | 0.776 |
| 6 | A | 548 | 574 | ACGNRASRKDKKVDEGKYTSKDGKESN | 27 | 0.708 |
| 7 | A | 111 | 123 | GVDRDQVDEGKYT | 13 | 0.706 |
| 8 | A | 45 | 62 | PGPGSEKVWVLGVDRDQN | 18 | 0.704 |
| 9 | A | 149 | 163 | GVGFALSDSVKKAAK | 15 | 0.705 |
| 10 | A | 253 | 264 | TNLDEAVSNDYG | 12 | 0.687 |
| 11 | A | 294 | 304 | DDVIEGQKNVG | 11 | 0.684 |
| 12 | A | 192 | 204 | AVSNDYKLIFGVG | 13 | 0.675 |
| 13 | A | 585 | 596 | IEGQKNVASKKA | 12 | 0.671 |
| 14 | A | 69 | 82 | EKGRTIAATQYAAG | 14 | 0.669 |
| 15 | A | 277 | 284 | LSEDAKKG | 8 | 0.664 |
| 16 | A | 458 | 471 | ACGNRASKSDKKFG | 14 | 0.664 |
| 17 | A | 639 | 647 | CGNRASRSD | 9 | 0.648 |
| 18 | A | 169 | 184 | GKKSNFVLASSLKKVG | 16 | 0.637 |
| 19 | A | 602 | 615 | ACGNRASKKDKKAG | 14 | 0.631 |
| 20 | A | 89 | 103 | TTSNLSEDAKKAVED | 15 | 0.611 |
| 21 | A | 441 | 450 | IKGQKNVASK | 10 | 0.599 |
| 22 | A | 533 | 542 | TGAGVFAKKA | 10 | 0.595 |
| 23 | A | 130 | 143 | PEKGRTIAATQYAA | 14 | 0.578 |
| 24 | A | 341 | 346 | SEYATN | 6 | 0.502 |

### 2.1.12 Supplement Table 12

Table S12: Clustering Scores from ClusPro Docking of Vaccine 1 and HLA-DRB1*01:01

| **Cluster** | **Members** | **Representative** | **Weighted Score** |
| --- | --- | --- | --- |
| 0 | 162 | Center | -835.5 |
|  |  | Lowest Energy | -960.5 |
| 1 | 118 | Center | -836.6 |
|  |  | Lowest Energy | -995 |
| 2 | 57 | Center | -885.7 |
|  |  | Lowest Energy | -885.7 |
| 3 | 42 | Center | -919.6 |
|  |  | Lowest Energy | -919.6 |
| 4 | 37 | Center | -845.7 |
|  |  | Lowest Energy | -914.7 |
| 5 |  | Center | -776.8 |
|  |  | Lowest Energy | -1009 |
| 6 | 34 | Center | -820.2 |
|  |  | Lowest Energy | -947.1 |
| 7 | 27 | Center | -843.2 |
|  |  | Lowest Energy | -902.5 |
| 8 | 25 | Center | -849.5 |
|  |  | Lowest Energy | -849.5 |
| 9 | 24 | Center | -791.7 |
|  |  | Lowest Energy | -850.9 |
| 10 | 19 | Center | -867.8 |
|  |  | Lowest Energy | -896.7 |
| 11 |  | Center | -833 |
|  |  | Lowest Energy | -857.7 |
| 12 |  | Center | -899.1 |
|  |  | Lowest Energy | -899.1 |
| 13 | 14 | Center | -779.3 |
|  |  | Lowest Energy | -780.4 |
| 14 | 13 | Center | -807.7 |
|  |  | Lowest Energy | -807.7 |
| 15 | 12 | Center | -839.4 |
|  |  | Lowest Energy | -853.4 |
| 16 |  | Center | -799.8 |
|  |  | Lowest Energy | -862.6 |
| 17 | 11 | Center | -821.6 |
|  |  | Lowest Energy | -821.6 |
| 18 | 10 | Center | -864.3 |
|  |  | Lowest Energy | -864.3 |
| 19 |  | Center | -840.2 |
|  |  | Lowest Energy | -840.2 |
| 20 |  | Center | -785.1 |
|  |  | Lowest Energy | -828.2 |
| 21 |  | Center | -782.1 |
|  |  | Lowest Energy | -814.8 |
| 22 |  | Center | -760.5 |
|  |  | Lowest Energy | -782.7 |
| 23 | 6 | Center | -752.6 |
|  |  | Lowest Energy | -795.4 |
| 24 |  | Center | -749.1 |
|  |  | Lowest Energy | -825.2 |

### 2.1.13 Supplement Table 13

Table S13: Clustering Scores from ClusPro Docking of Vaccine 1 and HLA-A*02:01

| **Cluster** | **Members** | **Representative** | **Weighted Score** |
| --- | --- | --- | --- |
| 0 | 72 | Center | -981.9 |
|  |  | Lowest Energy | -1041.9 |
| 1 | 69 | Center | -805.4 |
|  |  | Lowest Energy | -919.7 |
| 2 | 60 | Center | -792.5 |
|  |  | Lowest Energy | -858.2 |
| 3 | 38 | Center | -878.8 |
|  |  | Lowest Energy | -1129 |
| 4 | 35 | Center | -847.7 |
|  |  | Lowest Energy | -882.9 |
| 5 |  | Center | -836.1 |
|  |  | Lowest Energy | -888 |
| 6 | 29 | Center | -849.3 |
|  |  | Lowest Energy | -849.3 |
| 7 | 28 | Center | -856.5 |
|  |  | Lowest Energy | -957.8 |
| 8 |  | Center | -796.6 |
|  |  | Lowest Energy | -929.3 |
| 9 | 27 | Center | -817.3 |
|  |  | Lowest Energy | -951.7 |
| 10 |  | Center | -861.4 |
|  |  | Lowest Energy | -861.4 |
| 11 | 23 | Center | -885.1 |
|  |  | Lowest Energy | -1087.2 |
| 12 |  | Center | -882.5 |
|  |  | Lowest Energy | -915.6 |
| 13 | 20 | Center | -985.6 |
|  |  | Lowest Energy | -985.6 |
| 14 | 19 | Center | -891.1 |
|  |  | Lowest Energy | -891.1 |
| 15 | 17 | Center | -889 |
|  |  | Lowest Energy | -1012.6 |
| 16 |  | Center | -856.9 |
|  |  | Lowest Energy | -856.9 |
| 17 | 16 | Center | -810 |
|  |  | Lowest Energy | -947.5 |
| 18 |  | Center | -785.8 |
|  |  | Lowest Energy | -853.1 |
| 19 |  | Center | -829 |
|  |  | Lowest Energy | -861 |
| 20 |  | Center | -795.6 |
|  |  | Lowest Energy | -853.5 |
| 21 | 15 | Center | -850.4 |
|  |  | Lowest Energy | -853.2 |
| 22 | 14 | Center | -846.8 |
|  |  | Lowest Energy | -846.8 |
| 23 | 13 | Center | -848.1 |
|  |  | Lowest Energy | -848.1 |
| 24 | 12 | Center | -813.9 |
|  |  | Lowest Energy | -881.6 |
| 25 | 11 | Center | -803.8 |
|  |  | Lowest Energy | -1000.3 |
| 26 |  | Center | -829.8 |
|  |  | Lowest Energy | -829.8 |
| 27 |  | Center | -833.1 |
|  |  | Lowest Energy | -833.1 |
| 28 | 10 | Center | -844.7 |
|  |  | Lowest Energy | -876.6 |
| 29 |  | Center | -789.8 |
|  |  | Lowest Energy | -832.4 |

### 2.1.14 Supplement Table 14

Table S14: Clustering Scores from ClusPro Docking of Vaccine 1 and TLR2

| **Cluster** | **Members** | **Representative** | **Weighted Score** |
| --- | --- | --- | --- |
| 0 | 140 | Center | -749.4 |
|  |  | Lowest Energy | -873 |
| 1 | 80 | Center | -765.6 |
|  |  | Lowest Energy | -909.5 |
| 2 | 59 | Center | -766.5 |
|  |  | Lowest Energy | -859.8 |
| 3 |  | Center | -945.8 |
|  |  | Lowest Energy | -945.8 |
| 4 | 51 | Center | -745.7 |
|  |  | Lowest Energy | -828.6 |
| 5 | 45 | Center | -717.8 |
|  |  | Lowest Energy | -875.2 |
| 6 | 43 | Center | -783.2 |
|  |  | Lowest Energy | -847.5 |
| 7 | 40 | Center | -744 |
|  |  | Lowest Energy | -896.7 |
| 8 | 35 | Center | -862.4 |
|  |  | Lowest Energy | -862.4 |
| 9 | 30 | Center | -723.6 |
|  |  | Lowest Energy | -814.3 |
| 10 | 28 | Center | -789 |
|  |  | Lowest Energy | -851.9 |
| 11 | 26 | Center | -730.8 |
|  |  | Lowest Energy | -794.8 |
| 12 | 21 | Center | -783.9 |
|  |  | Lowest Energy | -783.9 |
| 13 | 18 | Center | -731.5 |
|  |  | Lowest Energy | -814.1 |
| 14 |  | Center | -808.5 |
|  |  | Lowest Energy | -826.4 |
| 15 | 17 | Center | -836.9 |
|  |  | Lowest Energy | -836.9 |
| 16 |  | Center | -835.8 |
|  |  | Lowest Energy | -835.8 |
| 17 | 16 | Center | -829.5 |
|  |  | Lowest Energy | -837.5 |
| 18 | 15 | Center | -778.3 |
|  |  | Lowest Energy | -778.3 |
| 19 | 14 | Center | -738.7 |
|  |  | Lowest Energy | -791.1 |
| 20 |  | Center | -721.9 |
|  |  | Lowest Energy | -746.9 |
| 21 | 13 | Center | -736.1 |
|  |  | Lowest Energy | -860 |
| 22 | 12 | Center | -810.5 |
|  |  | Lowest Energy | -810.5 |
| 23 | 11 | Center | -798.3 |
|  |  | Lowest Energy | -798.3 |
| 24 | 10 | Center | -729.5 |
|  |  | Lowest Energy | -757.9 |
| 25 | 8 | Center | -745.1 |
|  |  | Lowest Energy | -745.1 |
| 26 | 7 | Center | -724.9 |
|  |  | Lowest Energy | -807.1 |
| 27 | 3 | Center | -730.6 |
|  |  | Lowest Energy | -740.2 |

### 2.1.15 Supplement Table 15

Table S15: Clustering Scores from Cluspro Docking of Vaccine 1 and TLR4

| **Cluster** | **Members** | **Representative** | **Weighted Score** |
| --- | --- | --- | --- |
| 0 | 61 | Center | -776.4 |
|  |  | Lowest Energy | -999.7 |
| 1 | 56 | Center | -782.7 |
|  |  | Lowest Energy | -930.8 |
| 2 | 51 | Center | -833.7 |
|  |  | Lowest Energy | -927.8 |
| 3 | 39 | Center | -709.2 |
|  |  | Lowest Energy | -957 |
| 4 | 34 | Center | -701.7 |
|  |  | Lowest Energy | -805.2 |
| 5 | 32 | Center | -753.1 |
|  |  | Lowest Energy | -840.3 |
| 6 | 29 | Center | -741.8 |
|  |  | Lowest Energy | -861.2 |
| 7 |  | Center | -900.6 |
|  |  | Lowest Energy | -1012.8 |
| 8 |  | Center | -751.7 |
|  |  | Lowest Energy | -867.3 |
| 9 | 28 | Center | -836.5 |
|  |  | Lowest Energy | -934.5 |
| 10 |  | Center | -881.7 |
|  |  | Lowest Energy | -881.7 |
| 11 | 24 | Center | -810.8 |
|  |  | Lowest Energy | -1046.5 |
| 12 |  | Center | -716.4 |
|  |  | Lowest Energy | -852.9 |
| 13 | 23 | Center | -846.2 |
|  |  | Lowest Energy | -893.3 |
| 14 |  | Center | -696.6 |
|  |  | Lowest Energy | -852.8 |
| 15 | 22 | Center | -699.6 |
|  |  | Lowest Energy | -847.8 |
| 16 | 20 | Center | -824.3 |
|  |  | Lowest Energy | -824.3 |
| 17 | 19 | Center | -755.7 |
|  |  | Lowest Energy | -782.6 |
| 18 | 17 | Center | -705.9 |
|  |  | Lowest Energy | -804.2 |
| 19 | 16 | Center | -703.3 |
|  |  | Lowest Energy | -854.3 |
| 20 |  | Center | -761.9 |
|  |  | Lowest Energy | -906 |
| 21 |  | Center | -760.9 |
|  |  | Lowest Energy | -760.9 |
| 22 | 15 | Center | -715.2 |
|  |  | Lowest Energy | -751.8 |
| 23 | 14 | Center | -710 |
|  |  | Lowest Energy | -762.3 |
| 24 | 13 | Center | -691.3 |
|  |  | Lowest Energy | -746.7 |
| 25 | 12 | Center | -745.9 |
|  |  | Lowest Energy | -781.2 |
| 26 | 11 | Center | -709.8 |
|  |  | Lowest Energy | -760.1 |
| 27 |  | Center | -729.1 |
|  |  | Lowest Energy | -770.3 |
| 28 | 10 | Center | -735.8 |
|  |  | Lowest Energy | -846.6 |
| 29 |  | Center | -739.3 |
|  |  | Lowest Energy | -739.3 |

### 2.1.16 Supplement Table 16

Table S16: Clustering Scores from Cluspro Docking of Vaccine 2 and HLA-DRB1*01:01

| **Cluster** | **Members** | **Representative** | **Weighted Score** |
| --- | --- | --- | --- |
| 0 | 58 | Center | -1015.4 |
|  |  | Lowest Energy | -1015.4 |
| 1 | 53 | Center | -721.3 |
|  |  | Lowest Energy | -802.5 |
| 2 |  | Center | -803.5 |
|  |  | Lowest Energy | -815.4 |
| 3 | 32 | Center | -722 |
|  |  | Lowest Energy | -825.6 |
| 4 |  | Center | -706.4 |
|  |  | Lowest Energy | -907.7 |
| 5 | 31 | Center | -760.8 |
|  |  | Lowest Energy | -787.2 |
| 6 | 29 | Center | -877.7 |
|  |  | Lowest Energy | -877.7 |
| 7 | 26 | Center | -723 |
|  |  | Lowest Energy | -905.1 |
| 8 |  | Center | -758.8 |
|  |  | Lowest Energy | -901.4 |
| 9 | 21 | Center | -783 |
|  |  | Lowest Energy | -783 |
| 10 | 20 | Center | -823.9 |
|  |  | Lowest Energy | -823.9 |
| 11 |  | Center | -712.3 |
|  |  | Lowest Energy | -770.5 |
| 12 | 19 | Center | -776.3 |
|  |  | Lowest Energy | -782.3 |
| 13 |  | Center | -754.9 |
|  |  | Lowest Energy | -897.6 |
| 14 |  | Center | -802.1 |
|  |  | Lowest Energy | -802.4 |
| 15 |  | Center | -775.4 |
|  |  | Lowest Energy | -775.4 |
| 16 | 18 | Center | -711.5 |
|  |  | Lowest Energy | -851.6 |
| 17 |  | Center | -822.7 |
|  |  | Lowest Energy | -866.1 |
| 18 |  | Center | -803.2 |
|  |  | Lowest Energy | -805.9 |
| 19 | 17 | Center | -739.9 |
|  |  | Lowest Energy | -809.6 |
| 20 | 16 | Center | -741.2 |
|  |  | Lowest Energy | -830.3 |
| 21 | 15 | Center | -734.5 |
|  |  | Lowest Energy | -805.1 |
| 22 | 14 | Center | -739.4 |
|  |  | Lowest Energy | -796.3 |
| 23 | 13 | Center | -831.9 |
|  |  | Lowest Energy | -831.9 |
| 24 |  | Center | -814.1 |
|  |  | Lowest Energy | -814.1 |
| 25 |  | Center | -774.8 |
|  |  | Lowest Energy | -774.8 |
| 26 | 12 | Center | -753.1 |
|  |  | Lowest Energy | -874.4 |
| 27 |  | Center | -756.8 |
|  |  | Lowest Energy | -799.9 |
| 28 |  | Center | -726.6 |
|  |  | Lowest Energy | -839 |
| 29 |  | Center | -756.4 |
|  |  | Lowest Energy | -776.3 |

### 2.1.17 Supplement Table 17

Table S17: Clustering Scores from Cluspro Docking of Vaccine 2 and HLA-A*02:01

| **Cluster** | **Members** | **Representative** | **Weighted Score** |
| --- | --- | --- | --- |
| 0 | 93 | Center | -987.8 |
|  |  | Lowest Energy | -987.8 |
| 1 | 68 | Center | -753.3 |
|  |  | Lowest Energy | -903.9 |
| 2 | 58 | Center | -771.6 |
|  |  | Lowest Energy | -949.3 |
| 3 | 46 | Center | -827.5 |
|  |  | Lowest Energy | -827.5 |
| 4 | 42 | Center | -650.1 |
|  |  | Lowest Energy | -734.5 |
| 5 | 37 | Center | -702.4 |
|  |  | Lowest Energy | -979.6 |
| 6 |  | Center | -656.6 |
|  |  | Lowest Energy | -751.1 |
| 7 | 36 | Center | -803.7 |
|  |  | Lowest Energy | -803.7 |
| 8 | 30 | Center | -667.9 |
|  |  | Lowest Energy | -784 |
| 9 | 29 | Center | -684.8 |
|  |  | Lowest Energy | -866.7 |
| 10 | 26 | Center | -649.7 |
|  |  | Lowest Energy | -788.4 |
| 11 | 22 | Center | -655.3 |
|  |  | Lowest Energy | -821.8 |
| 12 | 20 | Center | -666.2 |
|  |  | Lowest Energy | -740.9 |
| 13 | 18 | Center | -754 |
|  |  | Lowest Energy | -754 |
| 14 | 17 | Center | -780.5 |
|  |  | Lowest Energy | -780.5 |
| 15 |  | Center | -722.6 |
|  |  | Lowest Energy | -733.2 |
| 16 |  | Center | -687.3 |
|  |  | Lowest Energy | -687.3 |
| 17 | 16 | Center | -673.3 |
|  |  | Lowest Energy | -732.1 |
| 18 |  | Center | -695.7 |
|  |  | Lowest Energy | -746.1 |
| 19 |  | Center | -650.8 |
|  |  | Lowest Energy | -735 |
| 20 | 15 | Center | -669.2 |
|  |  | Lowest Energy | -798.6 |
| 21 |  | Center | -698.9 |
|  |  | Lowest Energy | -722 |
| 22 | 13 | Center | -783.7 |
|  |  | Lowest Energy | -783.7 |
| 23 |  | Center | -715.9 |
|  |  | Lowest Energy | -715.9 |
| 24 | 12 | Center | -691.5 |
|  |  | Lowest Energy | -718.2 |
| 25 |  | Center | -738.6 |
|  |  | Lowest Energy | -738.6 |
| 26 | 11 | Center | -671.3 |
|  |  | Lowest Energy | -718.7 |
| 27 |  | Center | -729.1 |
|  |  | Lowest Energy | -729.1 |
| 28 |  | Center | -675.4 |
|  |  | Lowest Energy | -770.5 |
| 29 | 10 | Center | -676 |
|  |  | Lowest Energy | -703.3 |

### 2.1.18 Supplement Table 18

Table S18: Clustering Scores from Cluspro Docking of Vaccine 2 and TLR2

| **Cluster** | **Members** | **Representative** | **Weighted Score** |
| --- | --- | --- | --- |
| 0 | 67 | Center | -722.7 |
|  |  | Lowest Energy | -789 |
| 1 | 58 | Center | -701.6 |
|  |  | Lowest Energy | -963.5 |
| 2 | 52 | Center | -673.6 |
|  |  | Lowest Energy | -776.7 |
| 3 | 40 | Center | -846.2 |
|  |  | Lowest Energy | -846.2 |
| 4 | 39 | Center | -696.5 |
|  |  | Lowest Energy | -783.5 |
| 5 | 34 | Center | -840.3 |
|  |  | Lowest Energy | -971 |
| 6 | 30 | Center | -850 |
|  |  | Lowest Energy | -876.2 |
| 7 | 29 | Center | -782.5 |
|  |  | Lowest Energy | -782.5 |
| 8 | 28 | Center | -766.7 |
|  |  | Lowest Energy | -827.3 |
| 9 | 27 | Center | -813.1 |
|  |  | Lowest Energy | -813.1 |
| 10 | 23 | Center | -748.6 |
|  |  | Lowest Energy | -765.5 |
| 11 |  | Center | -675.8 |
|  |  | Lowest Energy | -782.4 |
| 12 | 22 | Center | -677.2 |
|  |  | Lowest Energy | -767.1 |
| 13 |  | Center | -719.5 |
|  |  | Lowest Energy | -719.5 |
| 14 | 21 | Center | -734.8 |
|  |  | Lowest Energy | -846.7 |
| 15 |  | Center | -711.5 |
|  |  | Lowest Energy | -791.1 |
| 16 |  | Center | -782.4 |
|  |  | Lowest Energy | -782.4 |
| 17 |  | Center | -761.8 |
|  |  | Lowest Energy | -761.8 |
| 18 | 19 | Center | -912.9 |
|  |  | Lowest Energy | -912.9 |
| 19 | 18 | Center | -750.6 |
|  |  | Lowest Energy | -779.4 |
| 20 |  | Center | -792.6 |
|  |  | Lowest Energy | -792.6 |
| 21 | 14 | Center | -833 |
|  |  | Lowest Energy | -833 |
| 22 |  | Center | -810.4 |
|  |  | Lowest Energy | -810.4 |
| 23 |  | Center | -791.1 |
|  |  | Lowest Energy | -791.1 |
| 24 |  | Center | -771.2 |
|  |  | Lowest Energy | -771.2 |
| 25 |  | Center | -770.2 |
|  |  | Lowest Energy | -770.2 |
| 26 |  | Center | -700.6 |
|  |  | Lowest Energy | -709.7 |
| 27 | 13 | Center | -676 |
|  |  | Lowest Energy | -737.4 |
| 28 | 12 | Center | -739.5 |
|  |  | Lowest Energy | -739.5 |
| 29 |  | Center | -724.8 |
|  |  | Lowest Energy | -724.8 |

### 2.1.19 Supplement Table 19

Table S19: Clustering Scores from Cluspro Docking of Vaccine 2 and TLR4

| **Cluster** | **Members** | **Representative** | **Weighted Score** |
| --- | --- | --- | --- |
| 0 | 41 | Center | -697 |
|  |  | Lowest Energy | -801.8 |
| 1 | 37 | Center | -670.3 |
|  |  | Lowest Energy | -789 |
| 2 | 36 | Center | -705.2 |
|  |  | Lowest Energy | -887.8 |
| 3 | 32 | Center | -706.4 |
|  |  | Lowest Energy | -825.7 |
| 4 |  | Center | -765.1 |
|  |  | Lowest Energy | -765.1 |
| 5 | 30 | Center | -682.8 |
|  |  | Lowest Energy | -840.4 |
| 6 | 26 | Center | -858.6 |
|  |  | Lowest Energy | -858.6 |
| 7 | 23 | Center | -661.1 |
|  |  | Lowest Energy | -747.5 |
| 8 |  | Center | -742.3 |
|  |  | Lowest Energy | -774.9 |
| 9 | 20 | Center | -744.4 |
|  |  | Lowest Energy | -758.8 |
| 10 | 19 | Center | -663.2 |
|  |  | Lowest Energy | -745 |
| 11 |  | Center | -690.2 |
|  |  | Lowest Energy | -691 |
| 12 | 18 | Center | -697.2 |
|  |  | Lowest Energy | -719.1 |
| 13 |  | Center | -776.5 |
|  |  | Lowest Energy | -827.4 |
| 14 |  | Center | -749.8 |
|  |  | Lowest Energy | -749.8 |
| 15 | 17 | Center | -762.9 |
|  |  | Lowest Energy | -818.4 |
| 16 |  | Center | -707.6 |
|  |  | Lowest Energy | -758 |
| 17 |  | Center | -659.6 |
|  |  | Lowest Energy | -725.8 |
| 18 | 16 | Center | -720.3 |
|  |  | Lowest Energy | -852.3 |
| 19 | 15 | Center | -664.6 |
|  |  | Lowest Energy | -749.5 |
| 20 |  | Center | -709.1 |
|  |  | Lowest Energy | -763 |
| 21 |  | Center | -741.6 |
|  |  | Lowest Energy | -784 |
| 22 | 14 | Center | -702.9 |
|  |  | Lowest Energy | -733.7 |
| 23 | 13 | Center | -674.6 |
|  |  | Lowest Energy | -830.1 |
| 24 |  | Center | -656.9 |
|  |  | Lowest Energy | -703 |
| 25 |  | Center | -650 |
|  |  | Lowest Energy | -739.9 |
| 26 |  | Center | -692.4 |
|  |  | Lowest Energy | -692.4 |
| 27 |  | Center | -691.7 |
|  |  | Lowest Energy | -741.1 |
| 28 | 12 | Center | -730.5 |
|  |  | Lowest Energy | -730.5 |
| 29 |  | Center | -701.4 |
|  |  | Lowest Energy | -701.4 |

### 2.1.20 Supplement Table 20

Table S20: The first MMPBSA results of vaccine-TLR2/TLR4/HLA-A*02:01/HLA-DRB1*01:01 delta (complex - receptor - ligand).

| Complex | ΔVDWAALS | ΔEEL | ΔEPB | ΔENPOLAR | ΔGGAS | ΔGSOLV | ΔTOTAL |
| --- | --- | --- | --- | --- | --- | --- | --- |
|  | Average（SD） | | | | | | |
| Vaccine1-HLA-A*02:01 | -97.84(7.54) | -1046.06(76.47) | 1076.14(77.14) | -10.32(0.96) | -1143.90(78.64) | 1065.82(76.46) | -78.08(8.91) |
| Vaccine1-HLA-DRB1*01:01 | -149.27(6.02) | -3931.30(123.83) | 3940.75(122.49) | -15.90(0.34) | -4080.57(124.45) | 3924.84(122.35) | -155.72(9.04) |
| Vaccine1-TLR2 | -126.91(10.36) | -1597.00(72.14) | 1640.58(68.12) | -16.25(0.88) | -1723.91(74.08) | 1624.32(67.72) | -99.58(12.69) |
| Vaccine1-TLR4 | -215.60(16.77) | -3366.65(143.65) | 3439.85(141.57) | -26.89(1.89) | -3582.25(145.89) | 3412.96(140.44) | -169.29(17.25) |
| Vaccine2-HLA-A*02:01 | -109.48(8.32) | -1866.30(69.79) | 1877.05(68.40) | -13.41(0.94) | -1975.78(71.86) | 1863.64(68.04) | -112.14(10.56) |
| Vaccine2-HLA-DRB1*01:01 | -202.55(11.84) | -3957.25(79.38) | 3990.76(76.16) | -25.65(0.98) | -4159.80(83.87) | 3965.11(75.74) | -194.70(16.43) |
| Vaccine2-TLR2 | -104.26(8.16) | -1167.72(59.79) | 1192.97(59.02) | -12.85(0.46) | -1271.98(61.73) | 1180.12(58.88) | -91.86(9.28) |
| Vaccine2-TLR4 | -184.05(9.06) | -2954.96(82.08) | 2979.01(77.90) | -22.00(0.65) | -3139.01(83.43) | 2957.02(77.79) | -181.99(14.23) |

### 2.1.21 Supplement Table 21

Table S21: The second MMPBSA results of vaccine-TLR2/TLR4/HLA-A*02:01/HLA-DRB1*01:01 delta (complex - receptor - ligand).

| Complex | ΔVDWAALS | ΔEEL | ΔEPB | ΔENPOLAR | ΔGGAS | ΔGSOLV | ΔTOTAL |
| --- | --- | --- | --- | --- | --- | --- | --- |
|  | Average（SD） | | | | | | |
| Vaccine1-HLA-A*02:01 | -88.16(6.23) | -925.12(52.47) | 957.62(49.74) | -9.84(0.63) | -1013.27(53.69) | 947.78(49.74) | -65.49(7.06) |
| Vaccine1-HLA-DRB1*01:01 | -154.21(7.22) | -3477.07(84.71) | 3508.75(83.38) | -16.32(0.49) | -3631.28(84.07) | 3492.43(83.41) | -138.85(8.77) |
| Vaccine1-TLR2 | -126.93(11.52) | -1337.14(61.88) | 1370.99(60.20) | -16.29(1.11) | -1464.07(64.33) | 1354.70(59.79) | -109.37(9.92) |
| Vaccine1-TLR4 | -176.89(10.94) | -3157.28(115.52) | 3205.87(116.31) | -23.01(0.99) | -3334.17(117.06) | 3182.86(115.94) | -151.31(15.92) |
| Vaccine2-HLA-A*02:01 | -143.92(8.35) | -1707.63(91.23) | 1756.93(88.94) | -17.99(0.75) | -1851.55(91.92) | 1738.94(88.78) | -112.61(10.66) |
| Vaccine2-HLA-DRB1*01:01 | -205.15(10.43) | -3703.38(91.51) | 3778.67(93.16) | -22.67(0.71) | -3908.53(96.77) | 3756.00(92.81) | -152.53(14.73) |
| Vaccine2-TLR2 | -96.64(6.39) | -1307.69(73.00) | 1316.47(70.21) | -11.97(0.43) | -1404.33(72.72) | 1304.50(70.08) | -99.83(11.13) |
| Vaccine2-TLR4 | -156.96(7.41) | -2608.20(79.76) | 2660.50(76.34) | -18.01(0.78) | -2765.16(81.38) | 2642.49(76.24) | -122.68(13.49) |

### 2.1.22 Supplement Table 22

Table S22: The third MMPBSA results of vaccine-TLR2/TLR4/HLA-A*02:01/HLA-DRB1*01:01 delta (complex - receptor - ligand).

| Complex | ΔVDWAALS | ΔEEL | ΔEPB | ΔENPOLAR | ΔGGAS | ΔGSOLV | ΔTOTAL |
| --- | --- | --- | --- | --- | --- | --- | --- |
|  | Average（SD） | | | | | | |
| Vaccine1-HLA-A*02:01 | -114.60(7.01) | -886.42(43.72) | 948.03(41.37) | -12.75(0.63) | -1001.02(44.68) | 935.28(41.27) | -65.74(8.22) |
| Vaccine1-HLA-DRB1*01:01 | -146.36(9.20) | -3560.78(109.26) | 3591.91(107.75) | -15.45(0.72) | -3707.14(111.63) | 3576.46(107.49) | -130.69(11.82) |
| Vaccine1-TLR2 | -122.25(6.66) | -942.21(73.99) | 1002.12(71.11) | -13.09(0.48) | -1064.46(75.67) | 989.03(70.79) | -75.43(13.03) |
| Vaccine1-TLR4 | -138.58(11.42) | -2792.22(82.18) | 2814.57(81.74) | -15.88(1.05) | -2930.81(85.59) | 2798.69(81.38) | -132.11(10.40) |
| Vaccine2-HLA-A*02:01 | -120.16(9.18) | -1814.48(78.71) | 1837.28(71.00) | -15.34(0.85) | -1934.64(77.37) | 1821.93(71.23) | -112.70(11.33) |
| Vaccine2-HLA-DRB1*01:01 | -213.39(9.36) | -3859.44(90.31) | 3879.18(83.17) | -24.46(0.88) | -4072.83(90.11) | 3854.72(83.01) | -218.12(16.43) |
| Vaccine2-TLR2 | -136.51(7.04) | -1004.91(57.21) | 1044.37(54.94) | -13.93(0.44) | -1141.42(57.99) | 1030.45(54.79) | -110.98(12.22) |
| Vaccine2-TLR4 | -170.46(7.52) | -2711.48(108.56) | 2755.21(102.50) | -20.13(0.75) | -2881.94(106.63) | 2735.08(102.47) | -146.86(10.21) |

### 2.1.23 Supplement Table 23

Table S23: Population coverage

| **Area** | **Class combined** | | |
| --- | --- | --- | --- |
|  | **coverage** | **AverageAhitb** | **pc90c** |
| Australia | 98.74% | 6.88 | 2.99 |
| Brazil | 99.57% | 8.5 | 4.03 |
| Canada | 65.41% | 1.07 | 0.29 |
| Chile | 98.10% | 6.72 | 2.36 |
| China | 98.04% | 7.74 | 3.12 |
| England | 99.84% | 10.2 | 4.81 |
| France | 99.89% | 11.02 | 5.53 |
| Germany | 99.84% | 10.1 | 4.87 |
| India | 96.77% | 7.12 | 2.32 |
| Indonesia | 80.54% | 3.49 | 0.51 |
| Italy | 99.92% | 12.56 | 6.77 |
| Japan | 99.68% | 8.63 | 4.42 |
| Korea; South | 98.55% | 7.69 | 3.34 |
| Mexico | 99.55% | 7.24 | 3.22 |
| Russia | 99.23% | 7.95 | 3.58 |
| Spain | 99.55% | 8.26 | 4.01 |
| Sudan | 96.94% | 7.08 | 2.22 |
| Thailand | 98.69% | 7.93 | 3.49 |
| Turkey | 85.31% | 3.53 | 0.68 |
| United States | 99.32% | 9.02 | 3.93 |
| **Average** | 95.67% | 7.64 | 3.32 |
| **Standard deviation** | 8.52 | 2.57 | 1.6 |

## 2.2 Supplement Figures

### 2.2.1 Supplement Figure 1


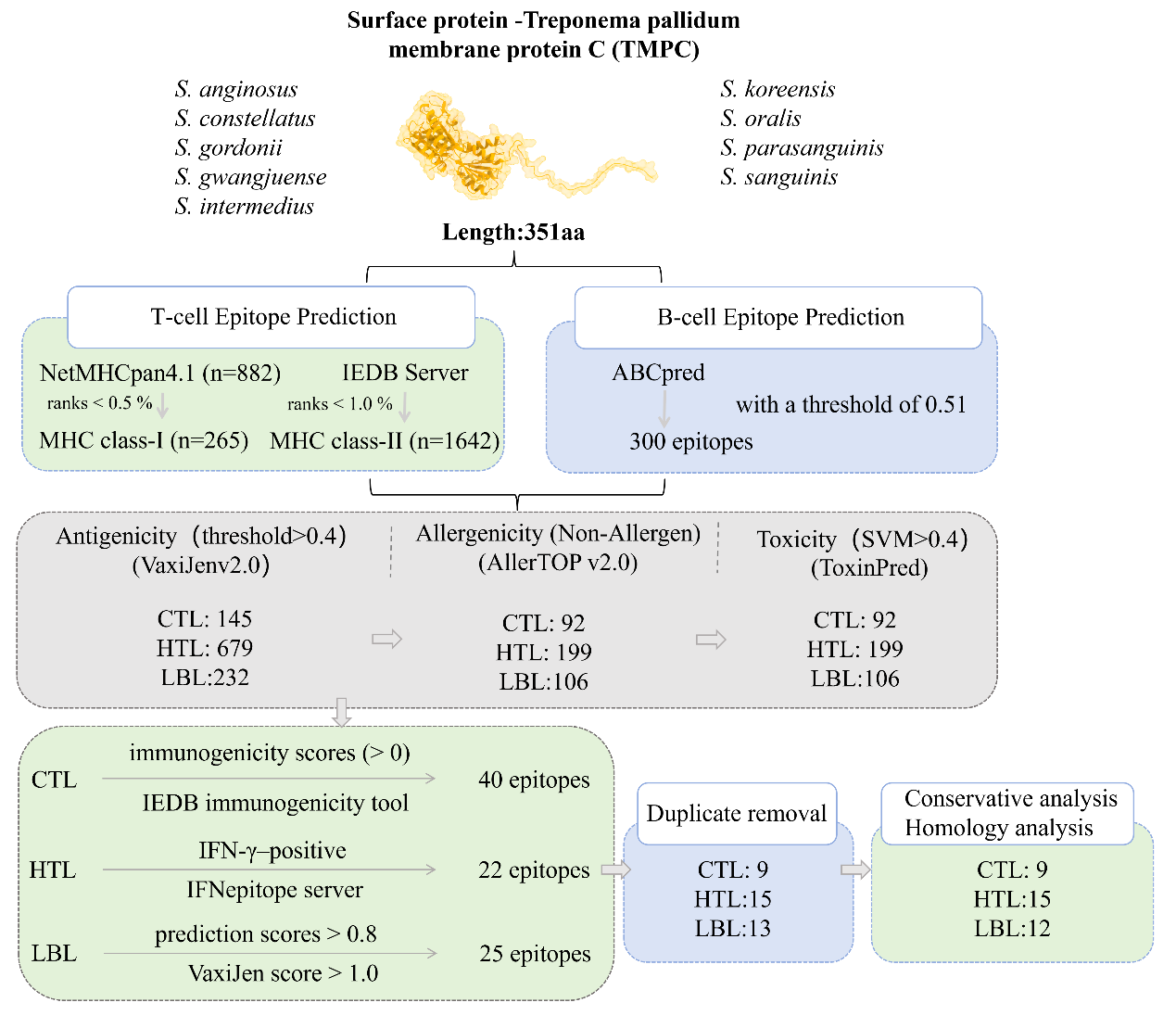


Supplement Figure 1

Workflow of epitope screening and selection

### 2.2.2 Supplement Figure 2


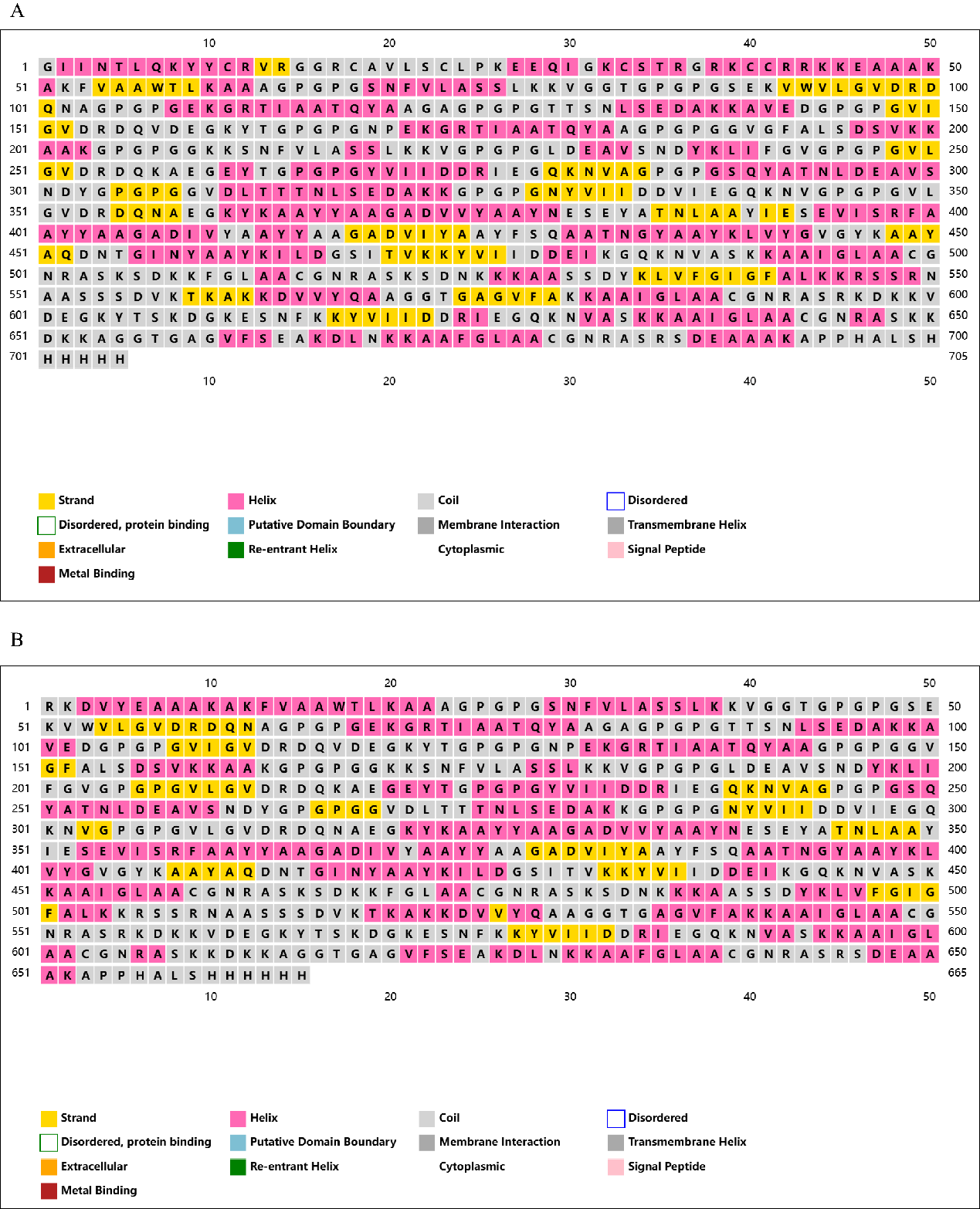


Supplement Figure 2

(A) The secondary structure of the vaccine 1 construct. (B) The secondary structure of the vaccine 2 construct.

### 2.2.3 Supplement Figure 3


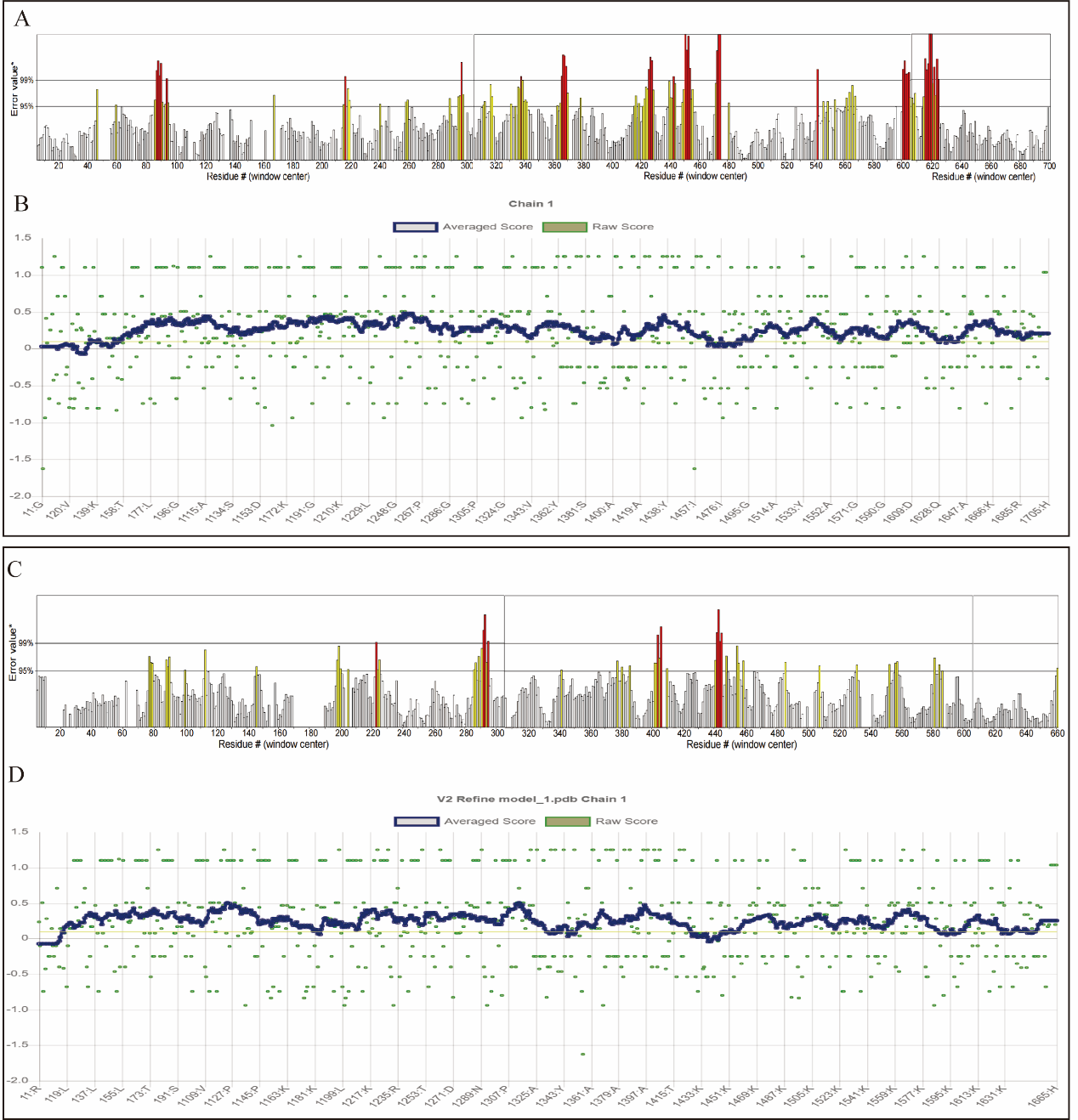


Supplement Figure 3

 (A, C) The ERRAT score of the refined 3D model of vaccine 1 and vaccine 2 generated by the ERRAT server. (B, D) The Vaccine 1 and Vaccine 2 ’s verified 3D score of residues: At least 80% of the amino acids have scored >= 0.1 in the 3D/1D profile.

### 2.2.4 Supplement Figure 4


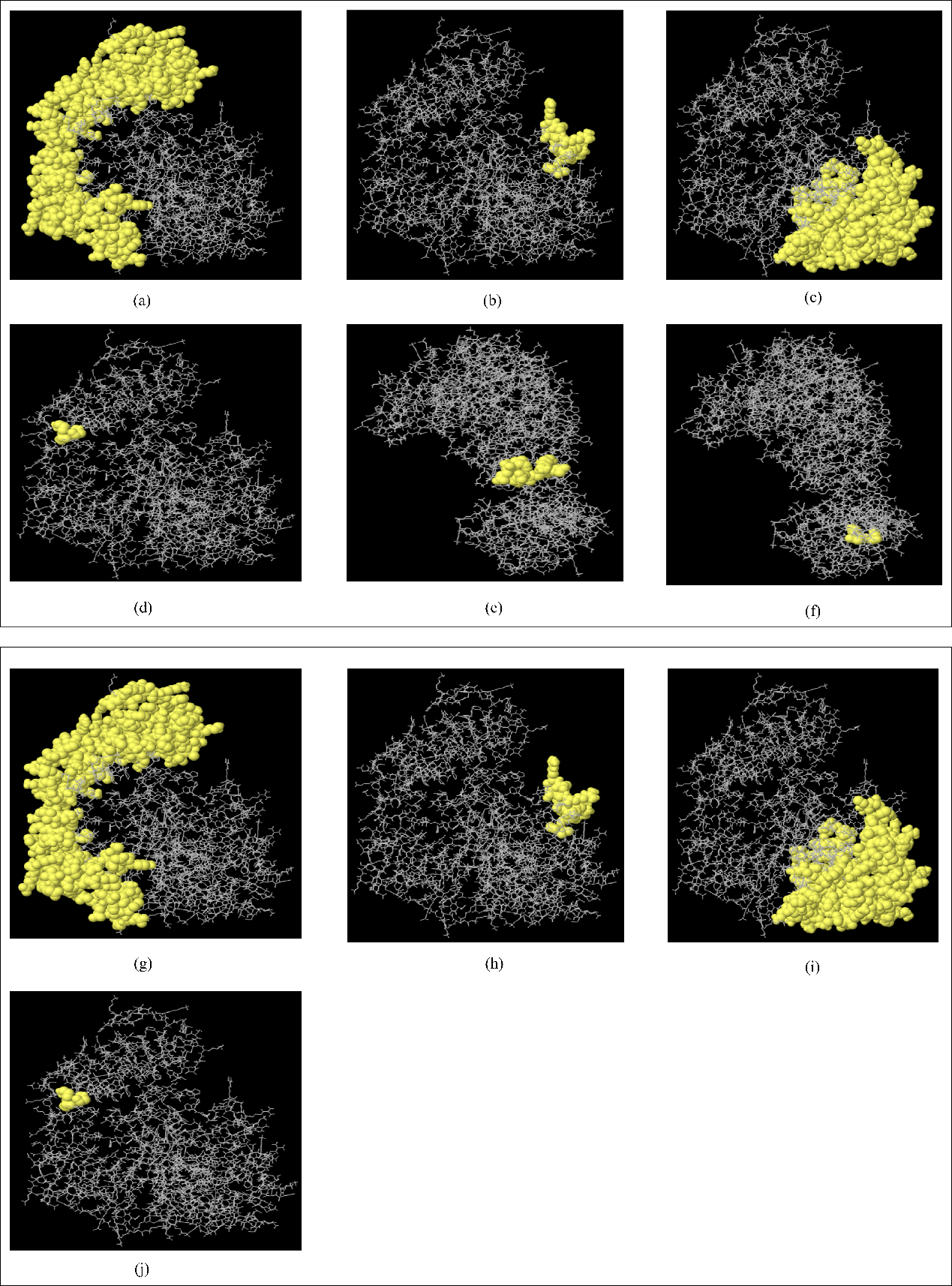


Supplement Figure 4

(a-f):3D structure of conformational B cells in vaccine 1. (g-j): 3D structure of conformational B cells in vaccine 2.

### 2.2.5 Supplement Figure 5


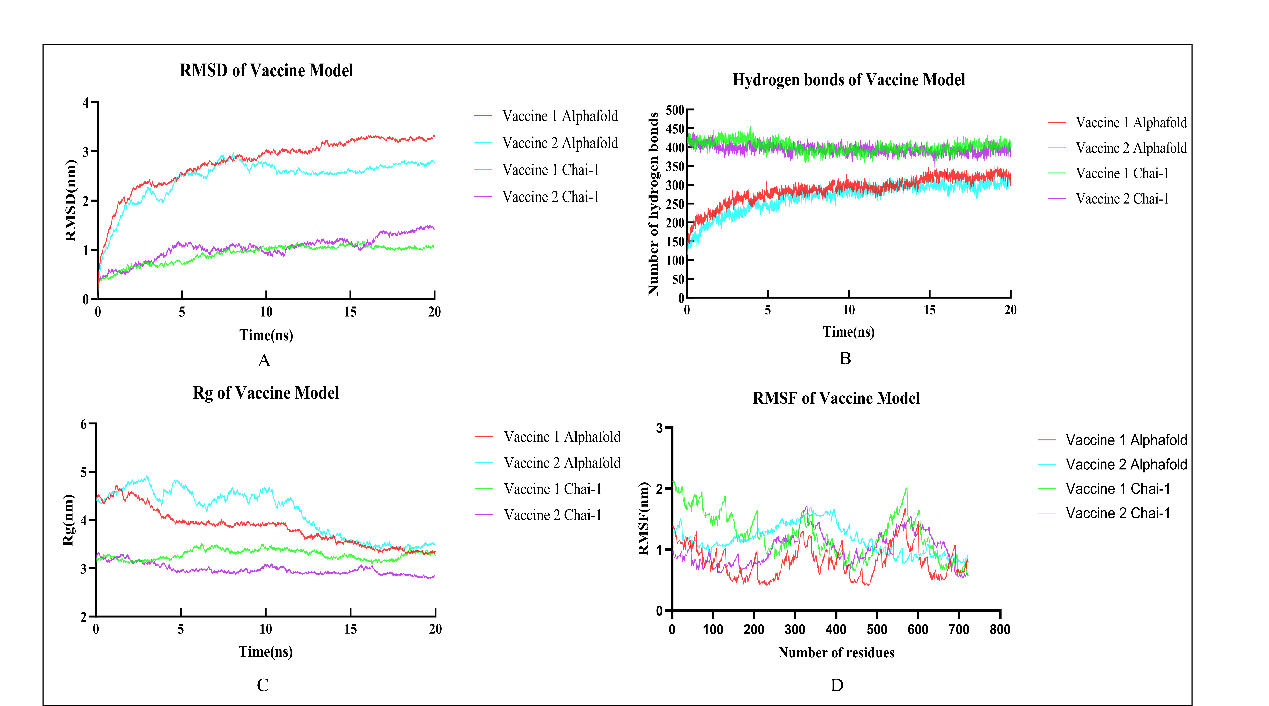


Supplement Figure 5

(A)The RMSD results of the stable vaccine structures obtained after modeling with Alphafold or Chai 1 and subjected to molecular dynamics simulation; (B) The Hydrogen bonds results of the stable vaccine structures obtained after modeling with Alphafold or Chai 1 and subjected to molecular dynamics simulation; (C) The Rg results of the stable vaccine structures obtained after modeling with Alphafold or Chai 1 and subjected to molecular dynamics simulation; (D) The RMSF results of the stable vaccine structures obtained after modeling with Alphafold or Chai 1 and subjected to molecular dynamics simulation.

### 2.2.6 Supplement Figure 6


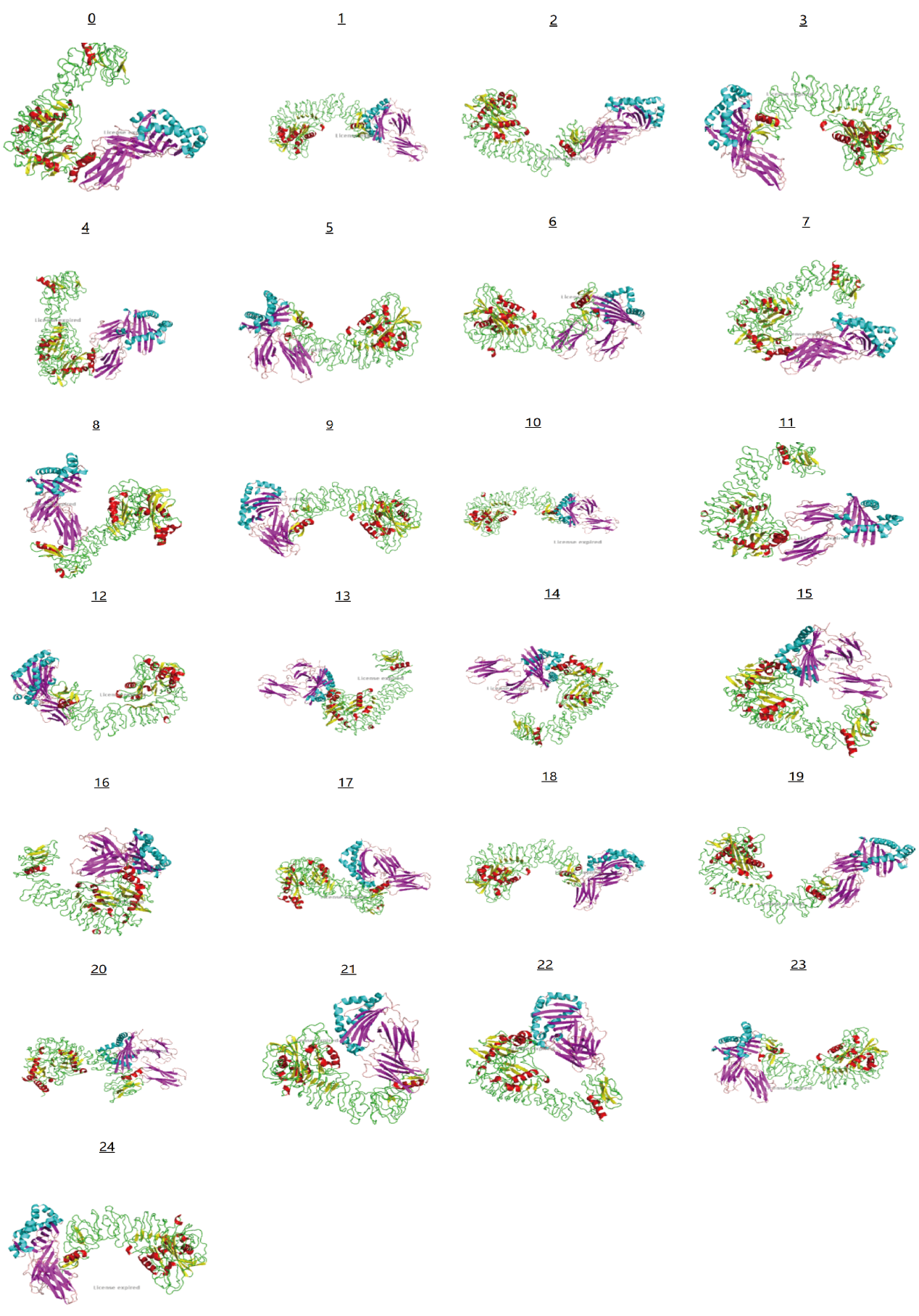


Supplement Figure 6

0-24: The ClusPro server displays 25 optimal docking results between Vaccine 1 and HLA-DRB1*01:01, based on cluster size ranking.

### 2.2.7 Supplement Figure 7


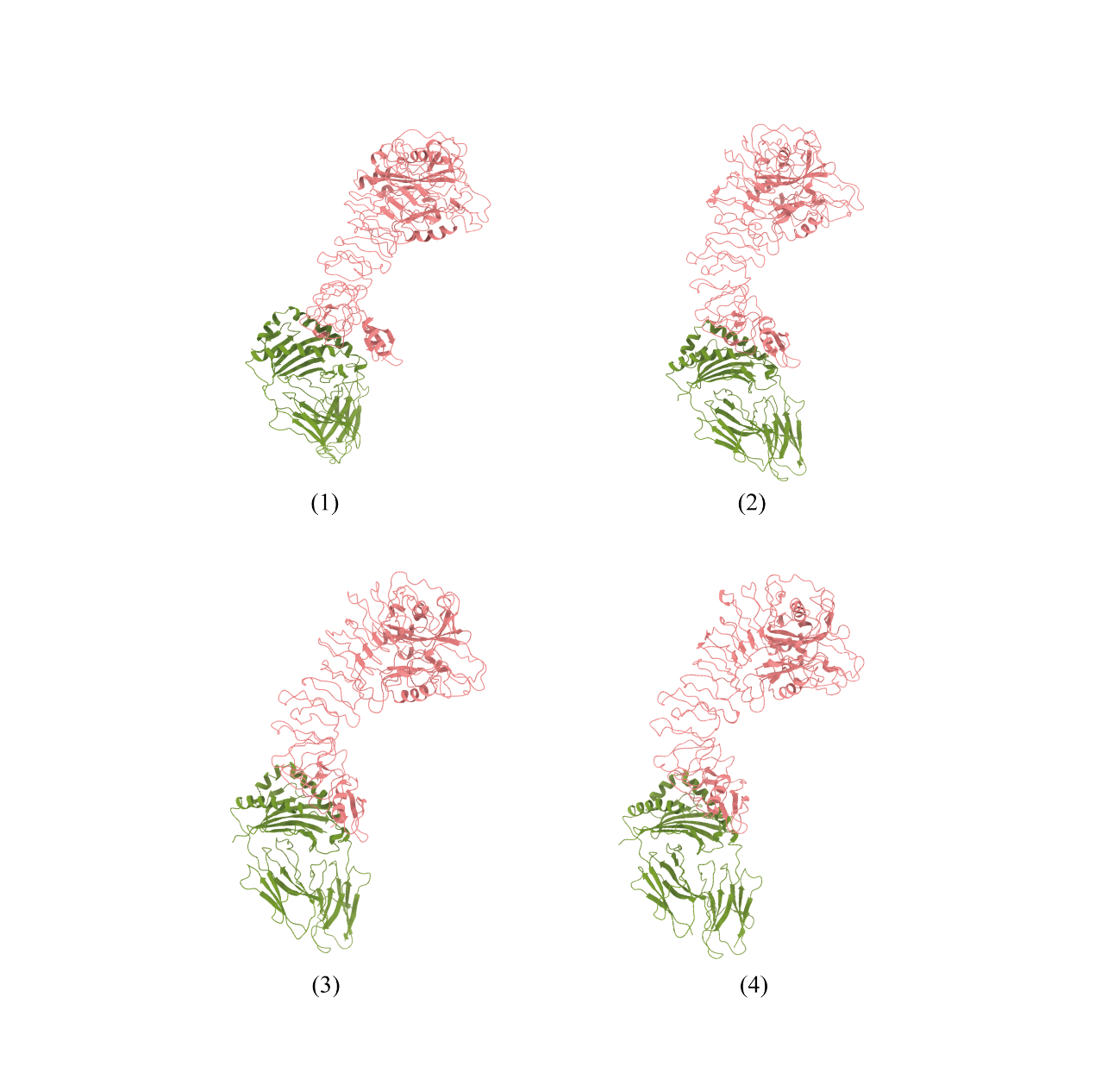


Supplement Figure 7

(1-4): Energy Scoring-Based Docking Results between Vaccine 1 and HLA-DRB1*01:01 refined by HADDOCK

### 2.2.8 Supplement Figure 8


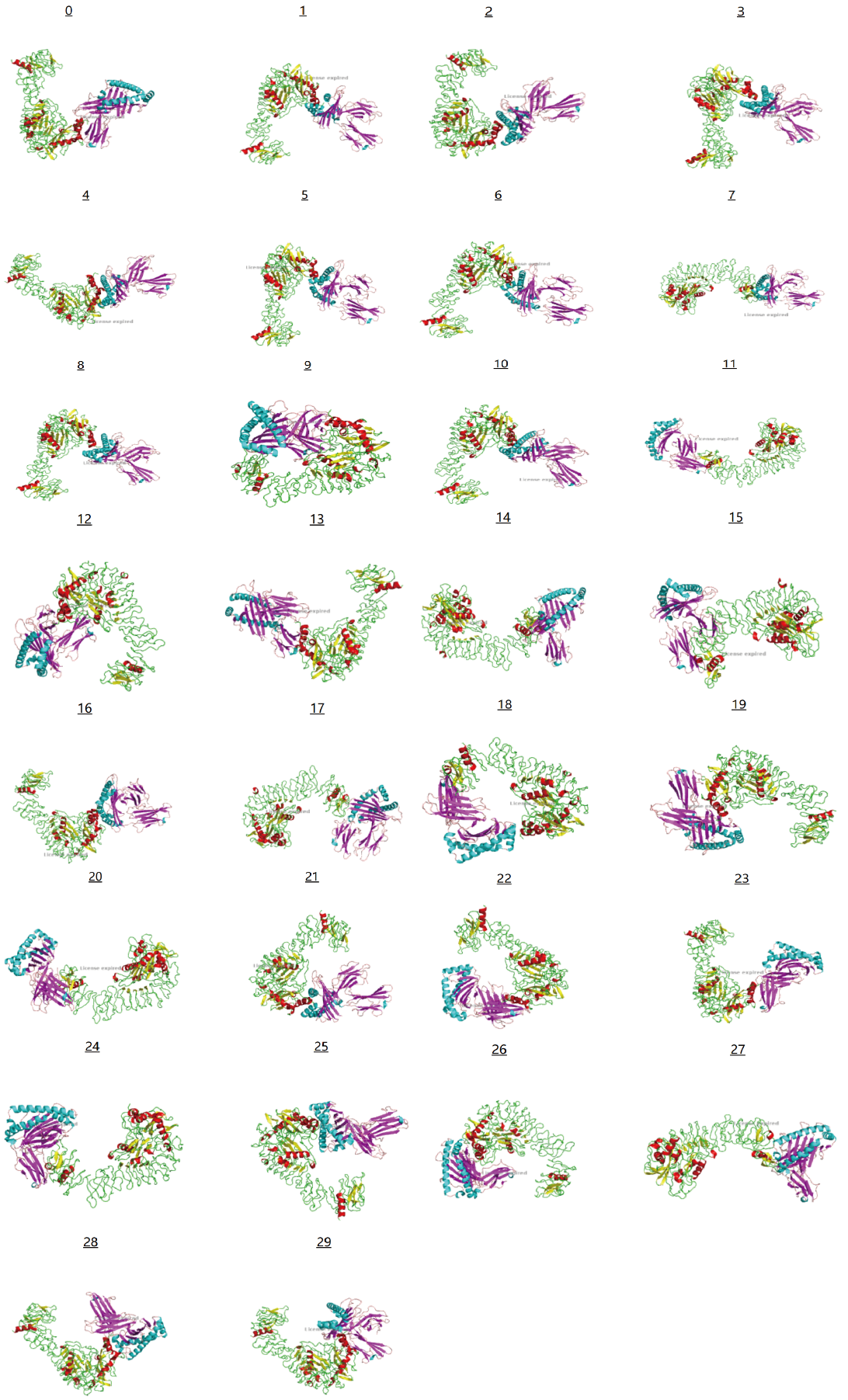


Supplement Figure 8

0-29: The ClusPro server displays 30 optimal docking results between Vaccine 1 and HLA-A*02:01, based on cluster size ranking.

### 2.2.9 Supplement Figure 9


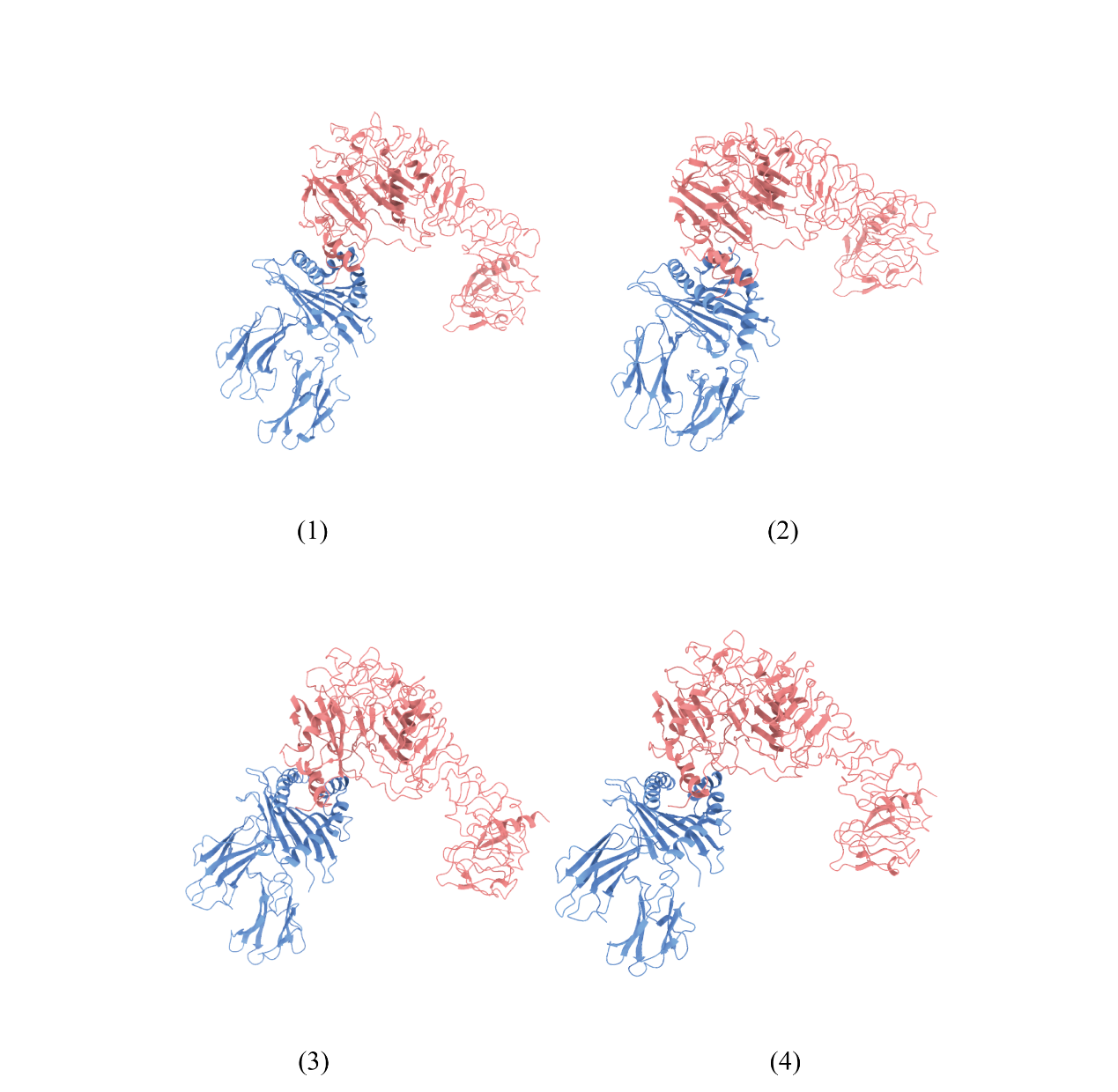


Supplement Figure 9

(1-4): Energy Scoring-Based Docking Results between Vaccine 1 and HLA-A*02:01 refined by HADDOCK

### 2.2.10 Supplement Figure 10


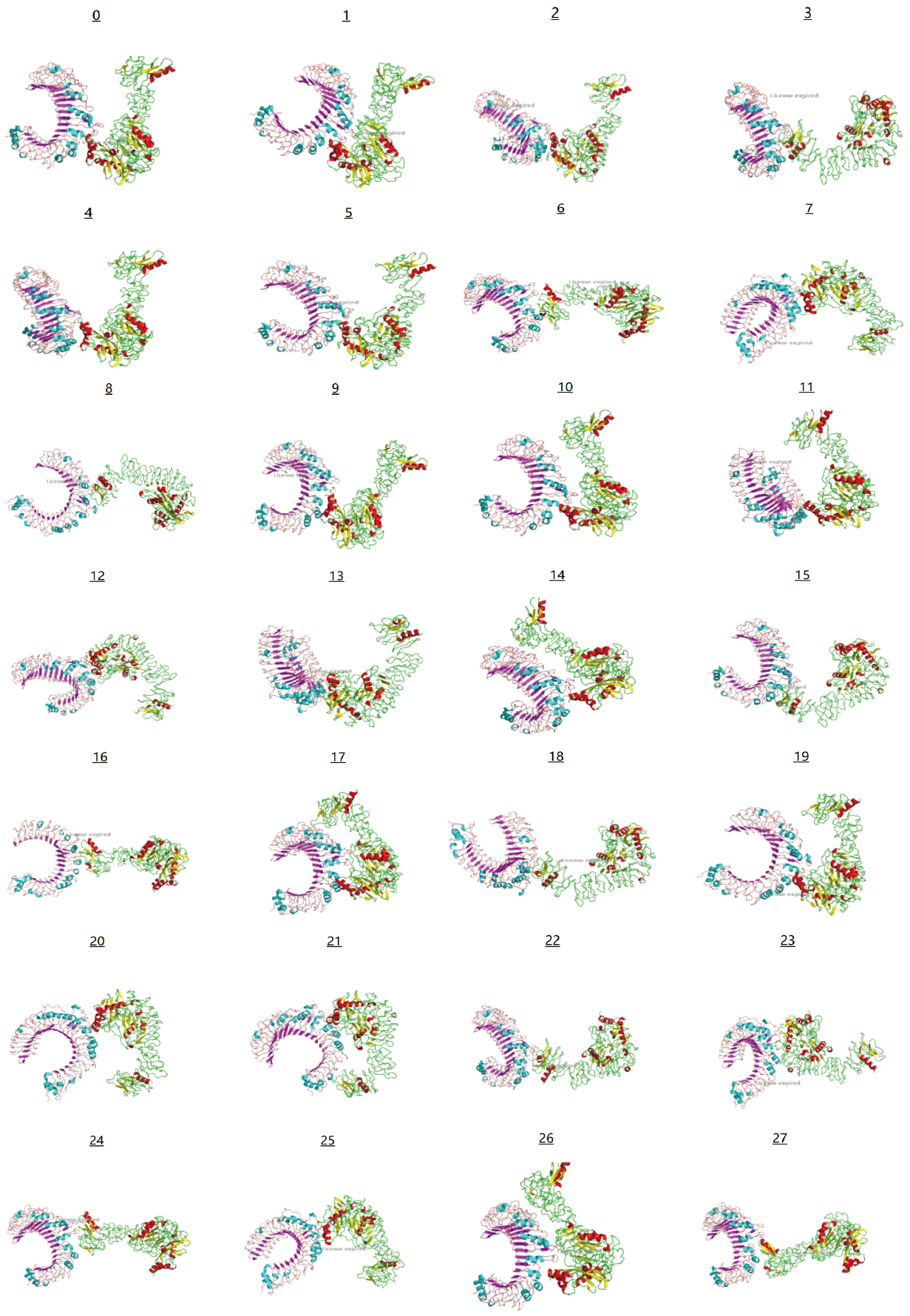


Supplement Figure 10

0-27: The ClusPro server displays 28 optimal docking results between Vaccine 1 and TLR2, based on cluster size ranking.

### 2.2.11 Supplement Figure 11


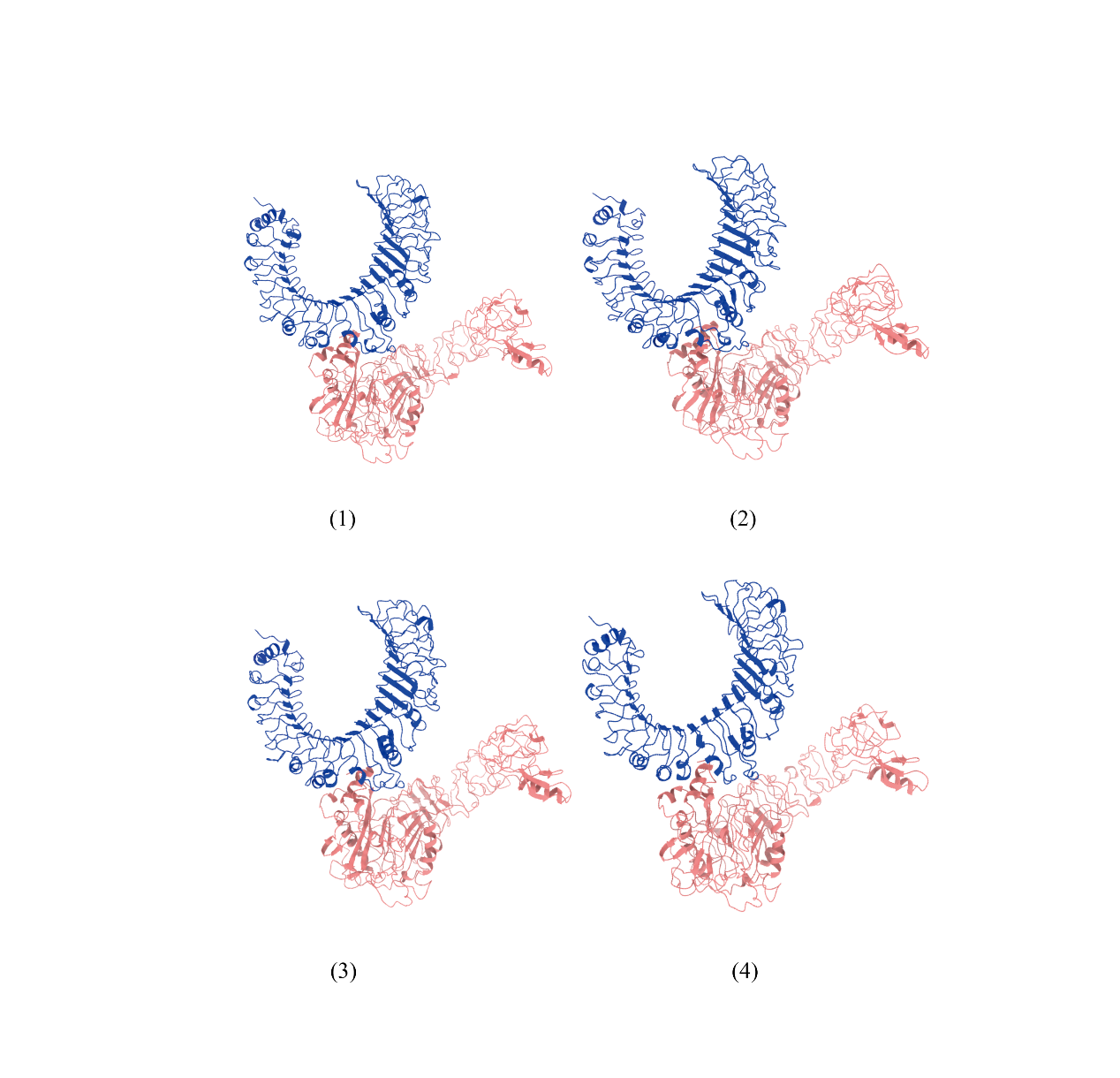


Supplement Figure 11

(1-4): Energy Scoring-Based Docking Results between Vaccine 1 and TLR2 refined by HADDOCK

### 2.2.12 Supplement Figure 12


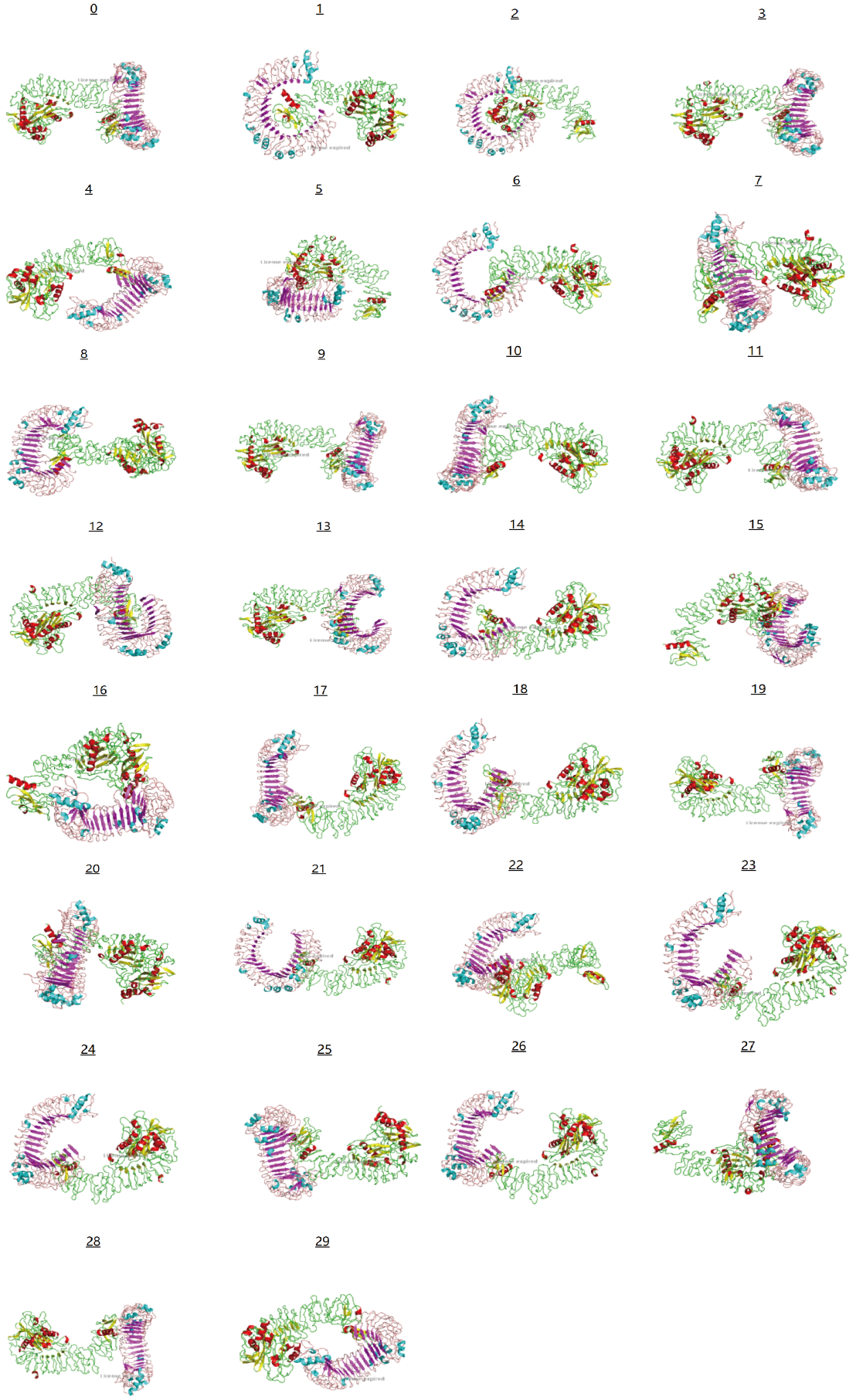


Supplement Figure 12

0-29: The ClusPro server displays 30 optimal docking results between Vaccine 1 and TLR4, based on cluster size ranking.

### 2.2.13 Supplement Figure 13


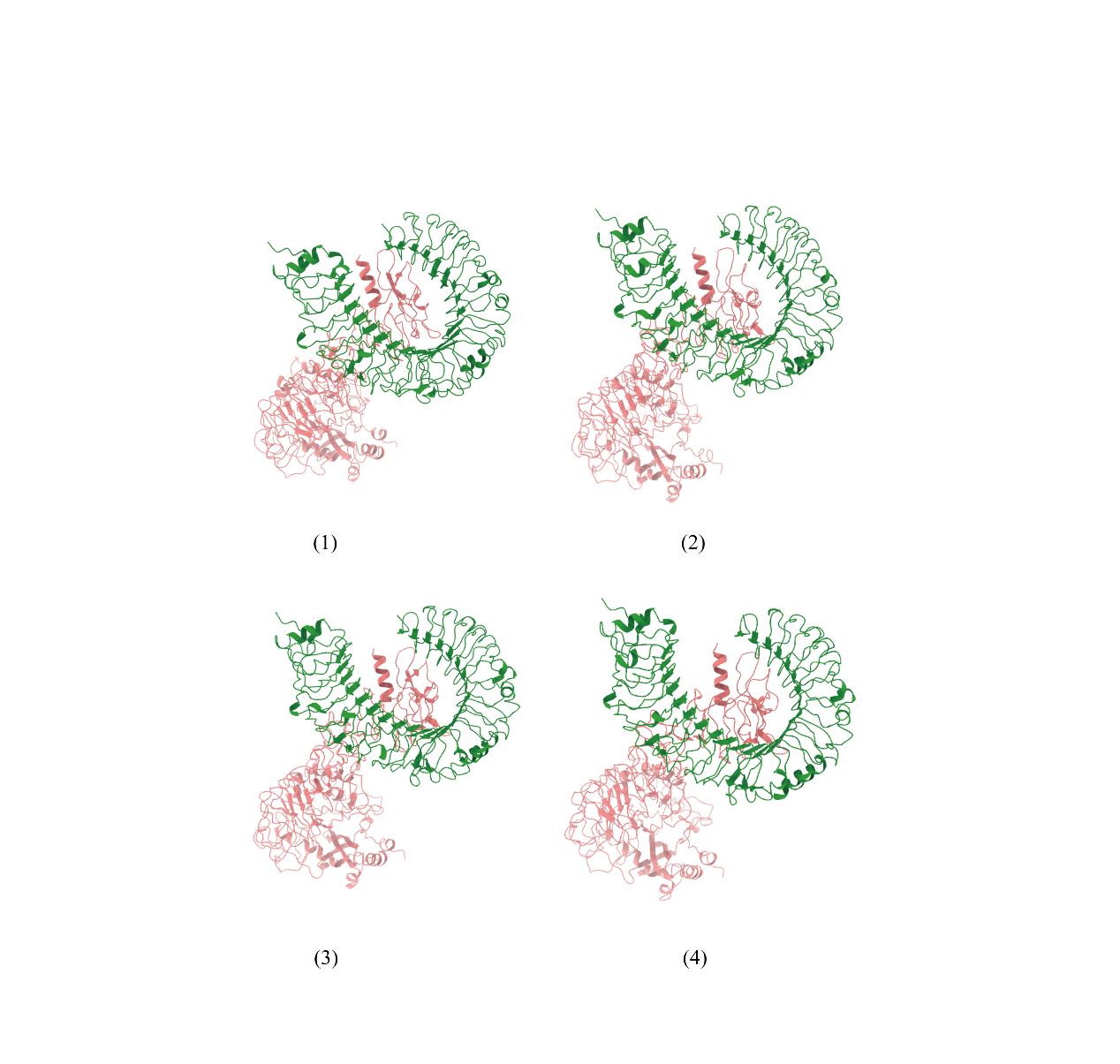


Supplement Figure 13

(1-4): Energy Scoring-Based Docking Results between Vaccine 1 and TLR4 refined by HADDOCK.

### 2.2.14 Supplement Figure 14


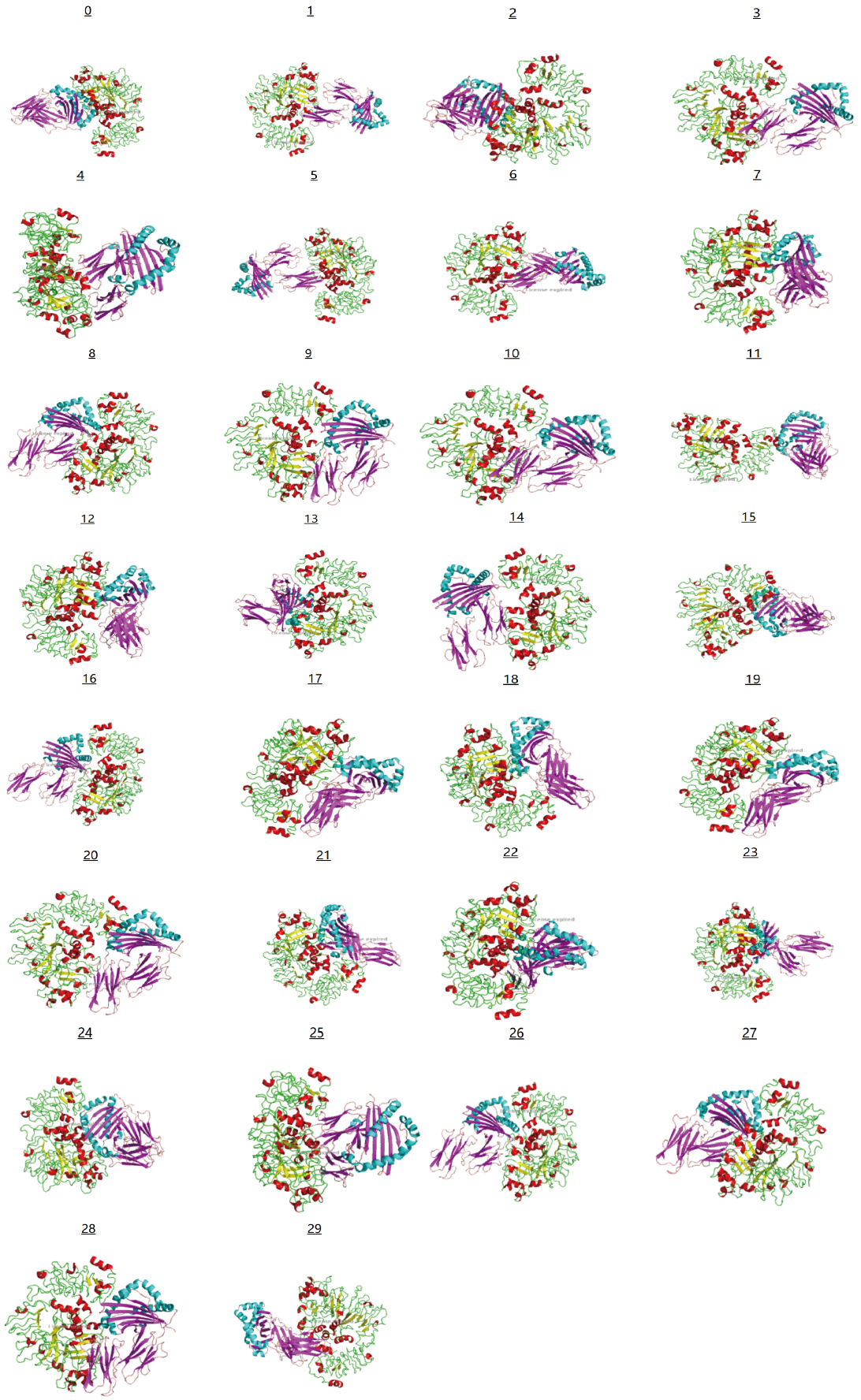


Supplement Figure 14

0-29: The ClusPro server displays 30 optimal docking results between Vaccine 2 and HLA-DRB1*01:01, based on cluster size ranking.

### 2.2.15 Supplement Figure 15


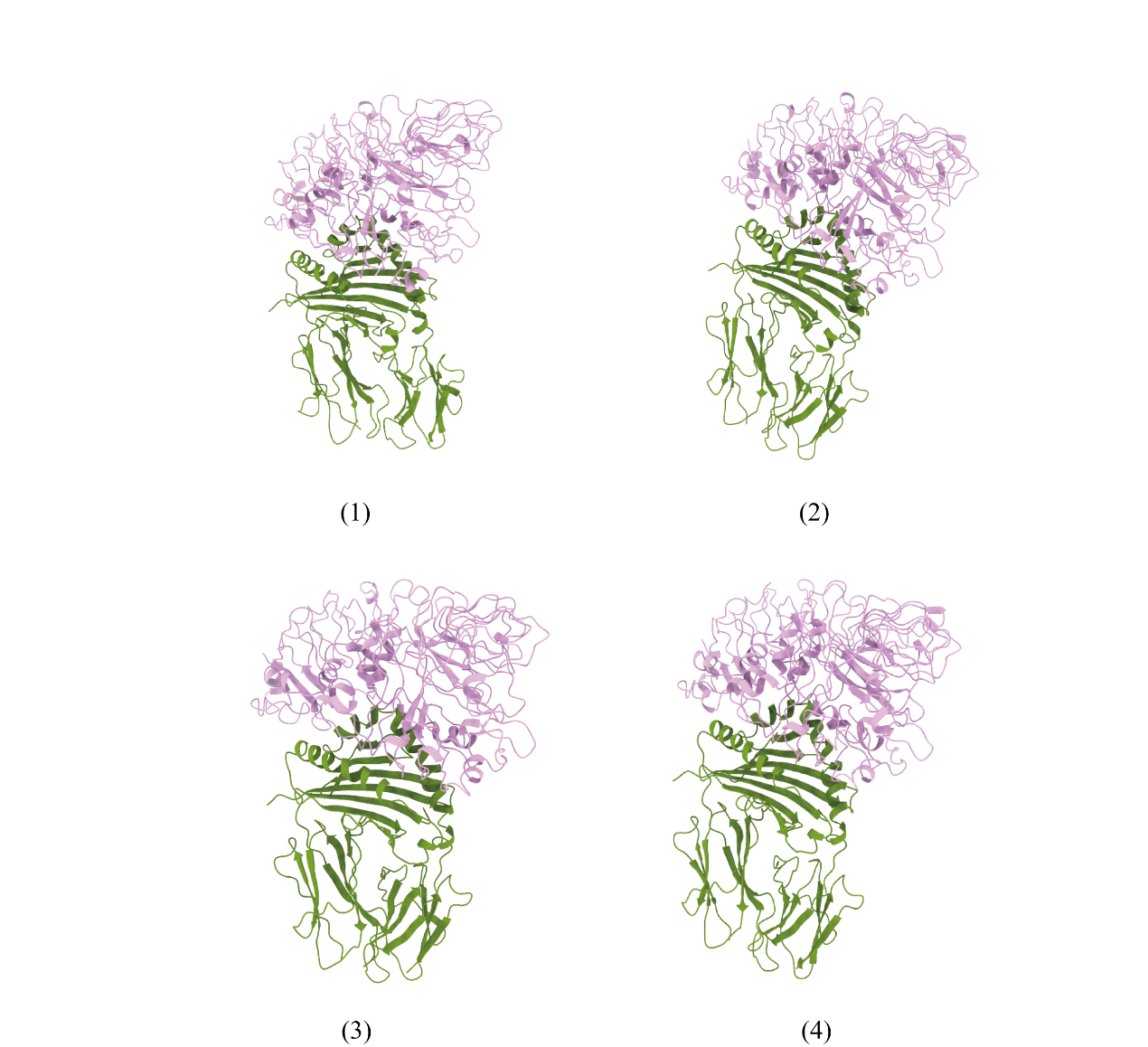


Supplement Figure 15

(1-4): Energy Scoring-Based Docking Results between Vaccine 2 and HLA-DRB1*01:01 refined by HADDOCK

### 2.2.16 Supplement Figure 16


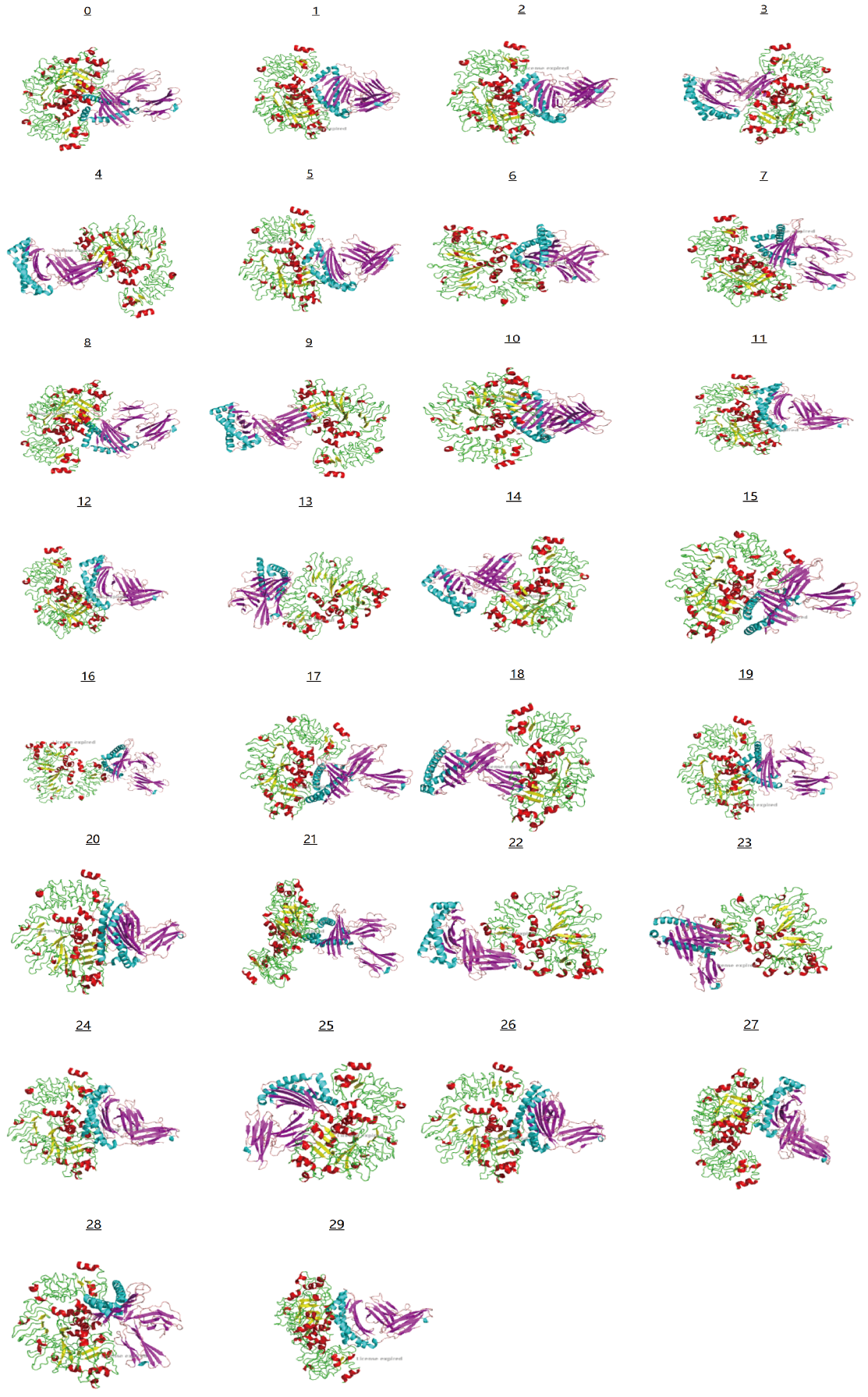


Supplement Figure 16

0-29: The ClusPro server displays 30 optimal docking results between Vaccine 2 and HLA-A*02:01, based on cluster size ranking.

### 2.2.17 Supplement Figure 17


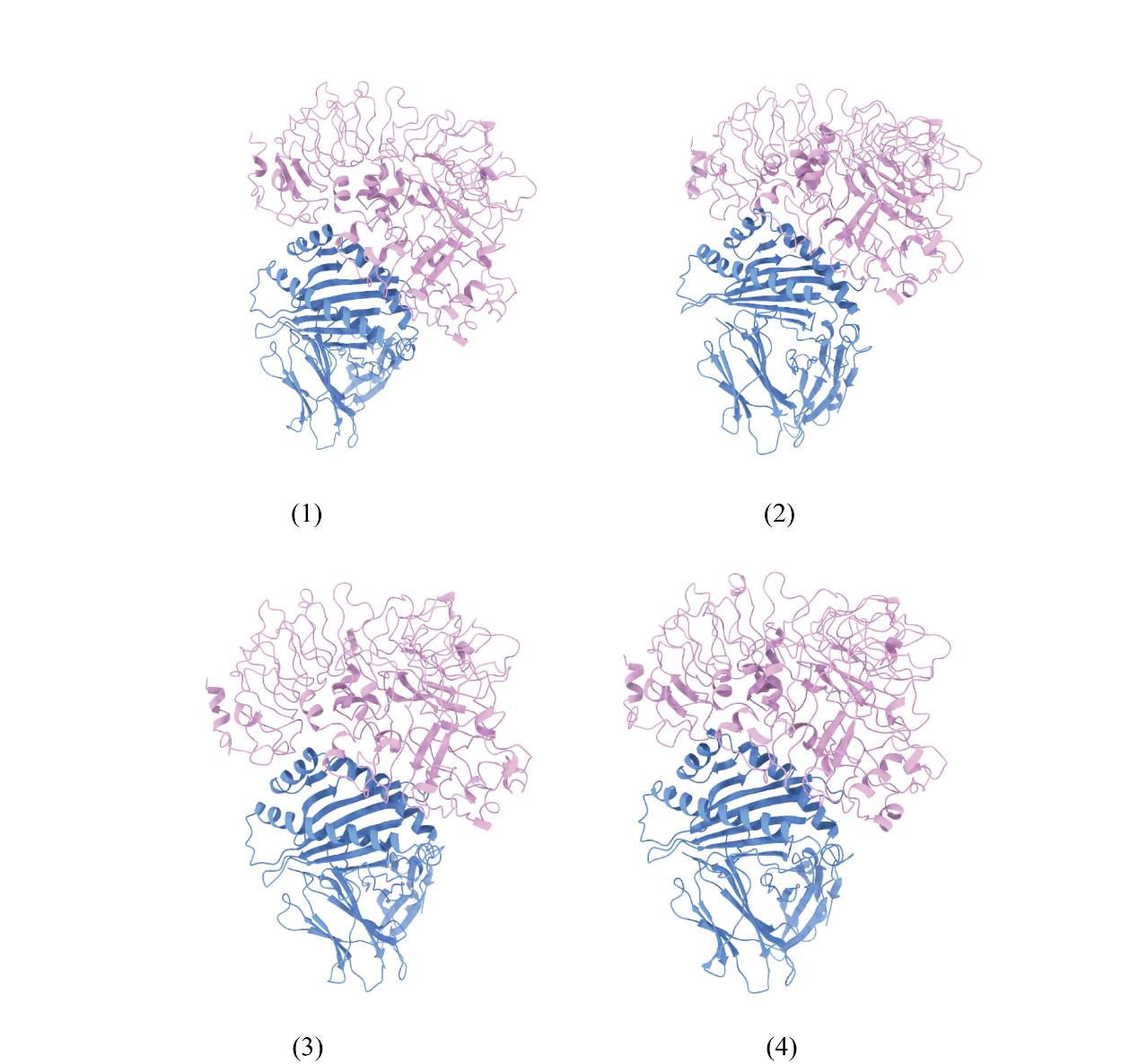


Supplement Figure 17

(1-4): Energy Scoring-Based Docking Results between Vaccine 2 and HLA-A*02:01 refined by HADDOCK

### 2.2.18 Supplement Figure 18


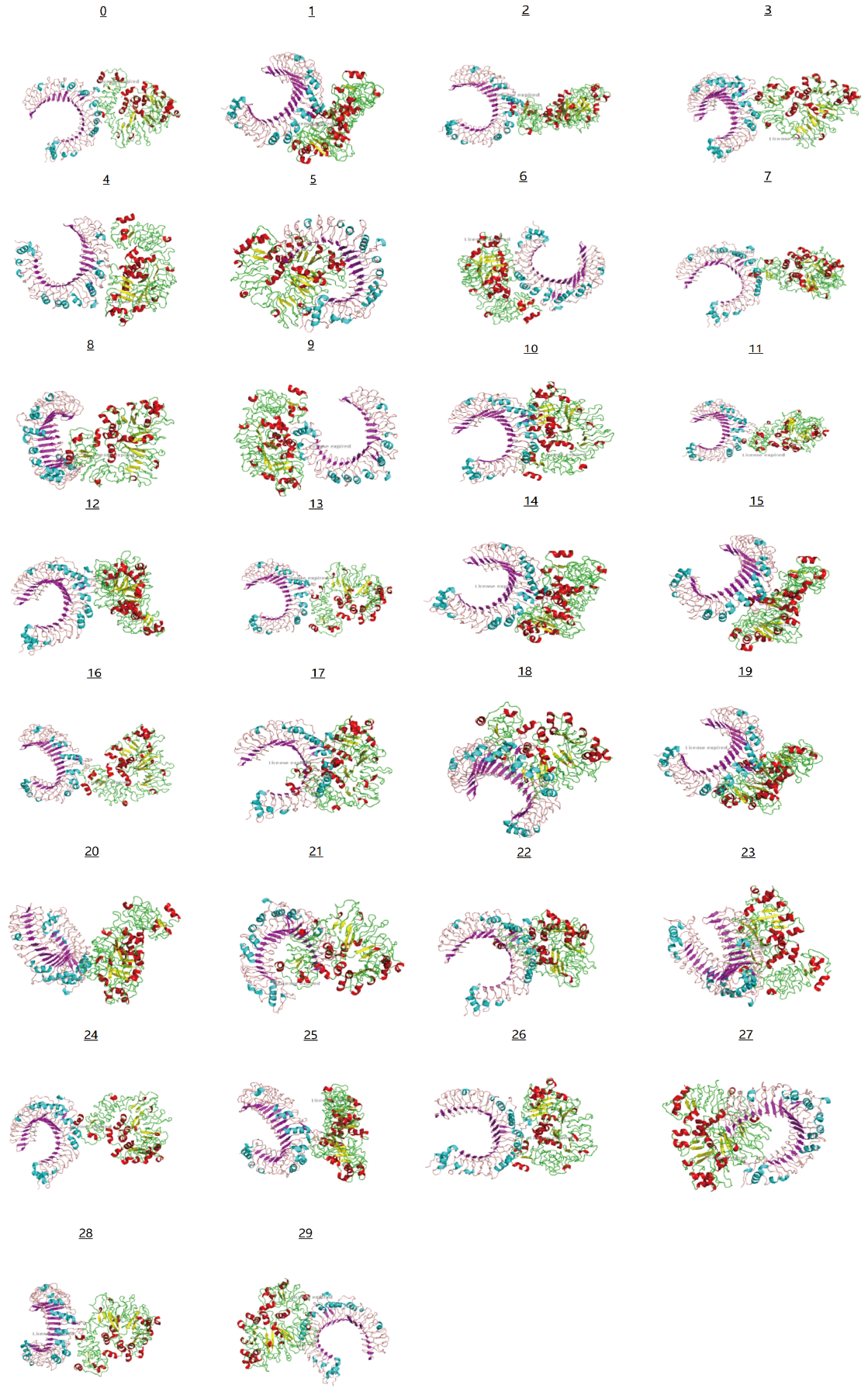


Supplement Figure 18

0-29: The ClusPro server displays 30 optimal docking results between Vaccine 2 and TLR2, based on cluster size ranking.

### 2.2.19 Supplement Figure 19


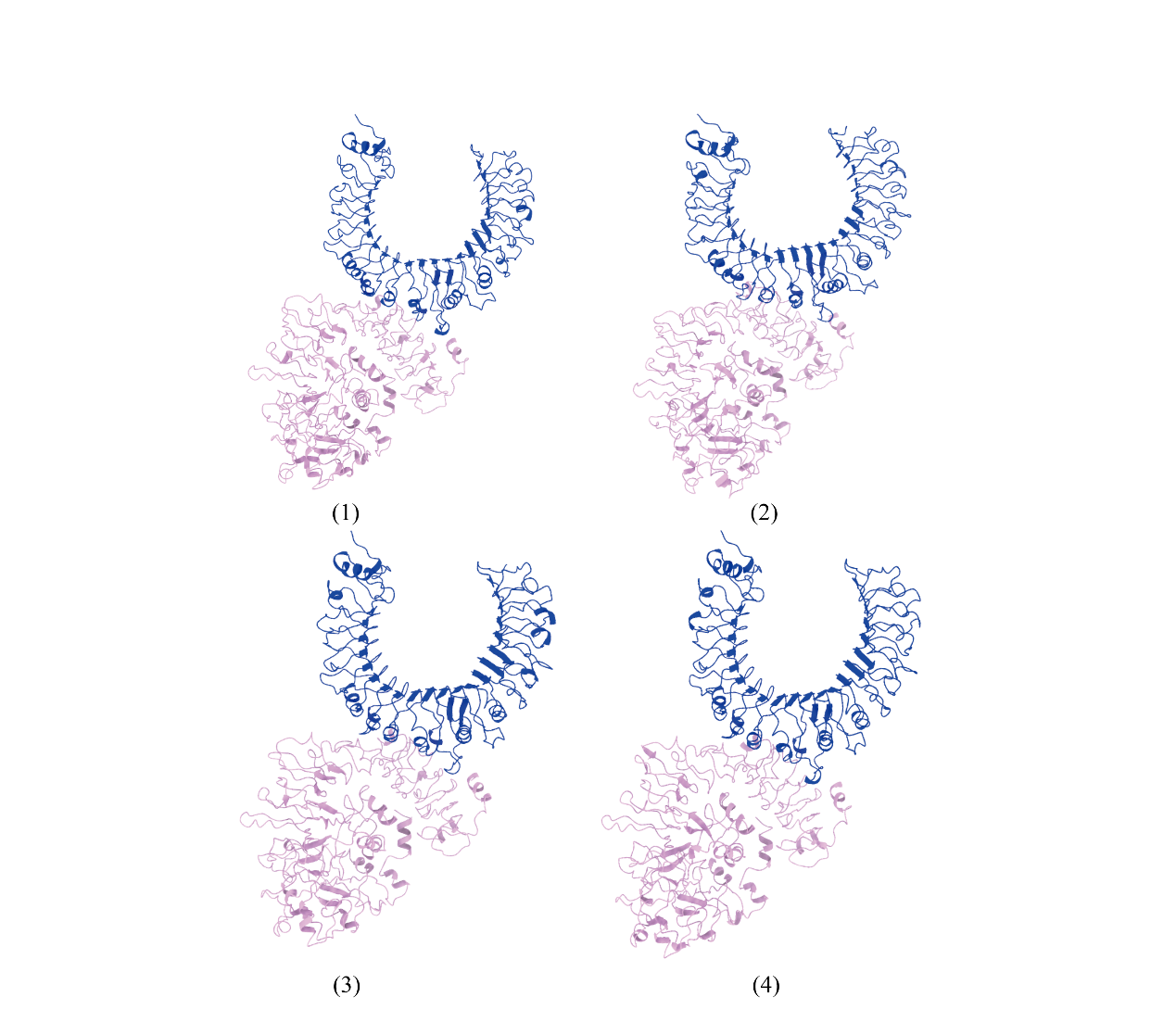


Supplement Figure 19

(1-4): Energy Scoring-Based Docking Results between Vaccine 2 and TLR2 refined by HADDOCK

### 2.2.20 Supplement Figure 20


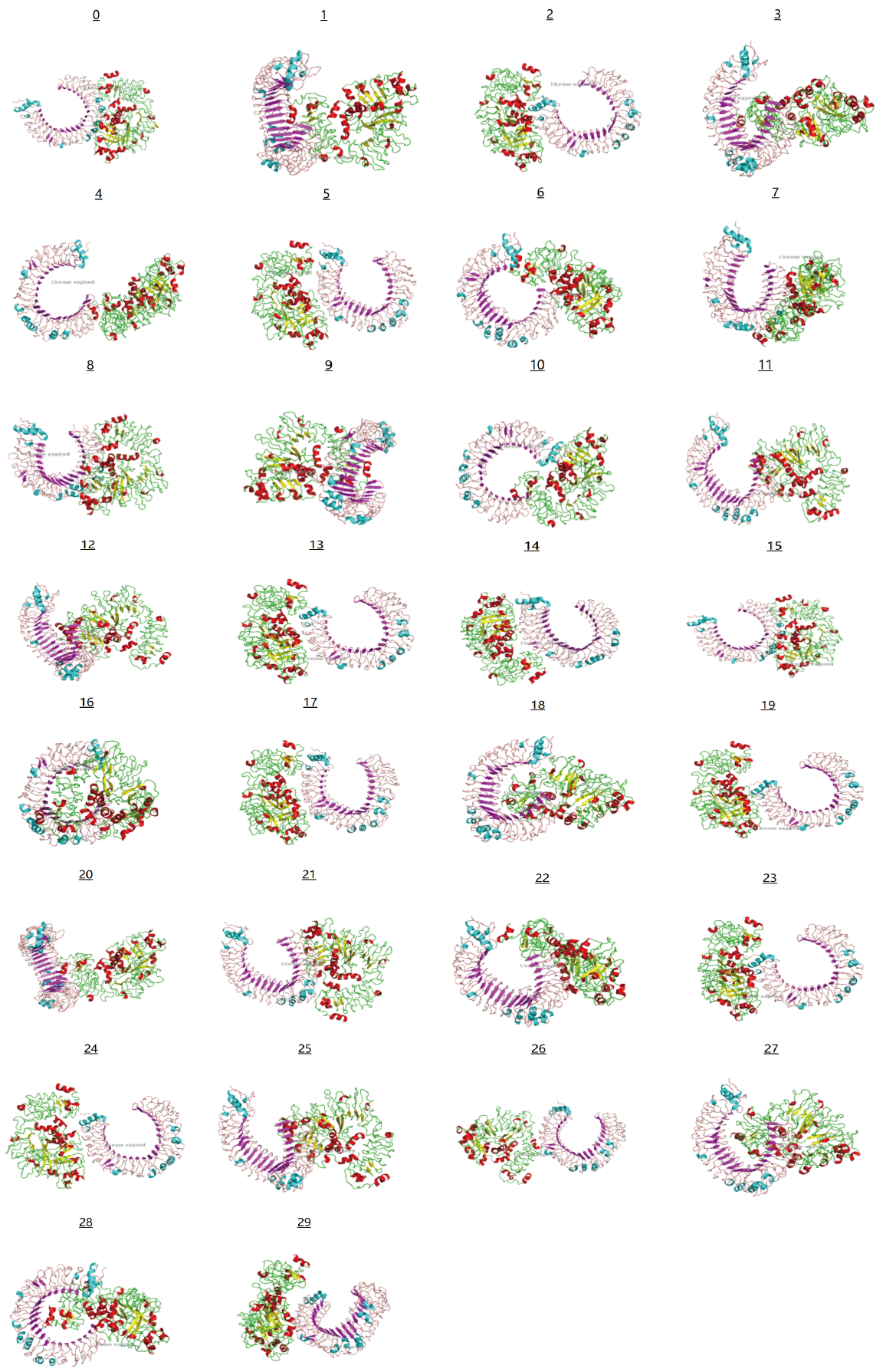


Supplement Figure 20

0-29: The ClusPro server displays 30 optimal docking results between Vaccine 2 and TLR4, based on cluster size ranking.

### 2.2.21 Supplement Figure 21


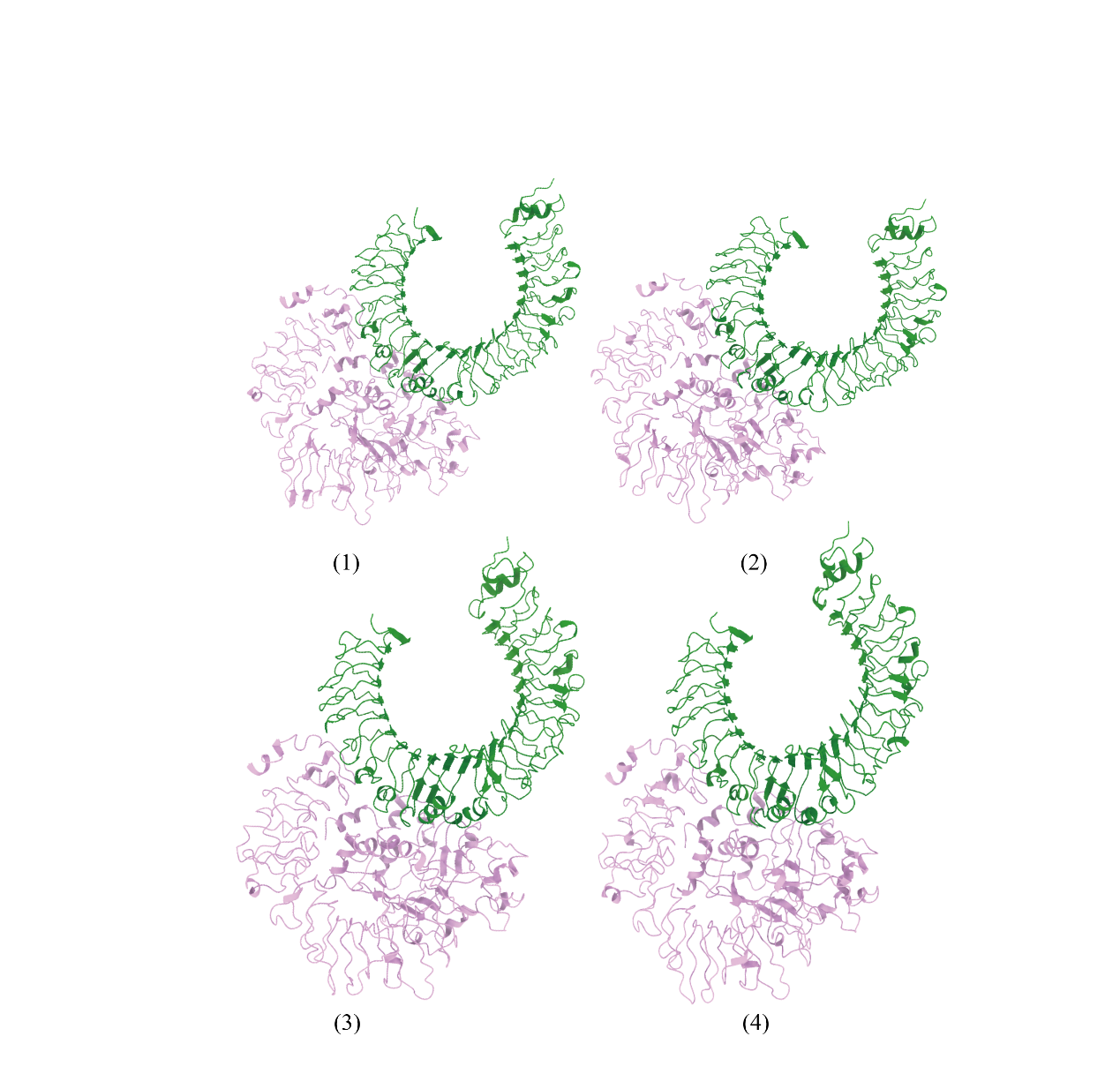


Supplement Figure 21

(1-4): Energy Scoring-Based Docking Results between Vaccine 2 and TLR4 refined by HADDOCK

### 2.2.22 Supplement Figure 22


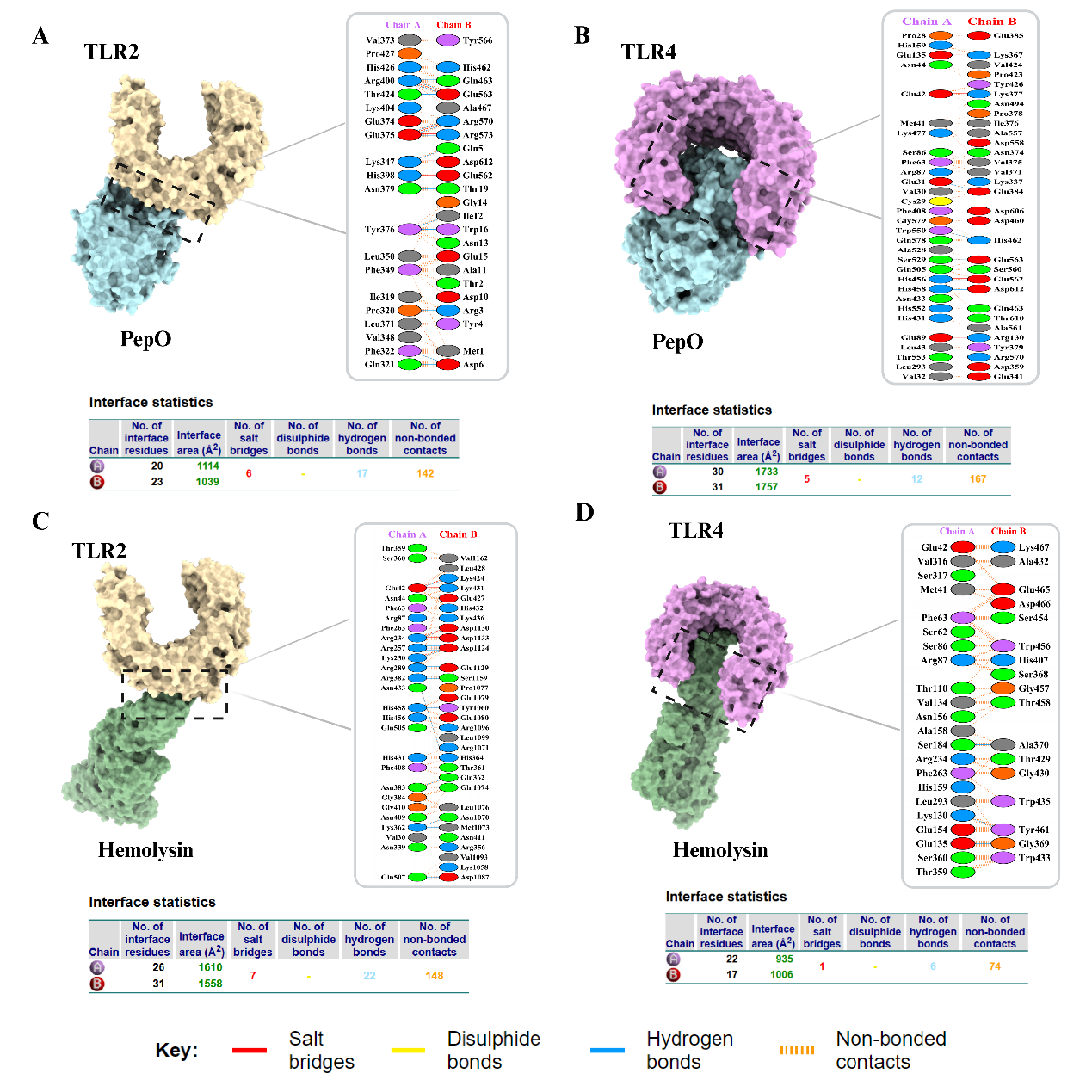


Supplement Figure 22: Visualization of docking and interacting residues of PepO and Hemolysin protein with TLR complexes

(A) PepO-TLR2 complex;(B) PepO-TLR4 complex; (C) Hemolysin-TLR 2 complex; (D) Hemolysin-TLR 4 complex

### 2.2.23 Supplement Figure 23


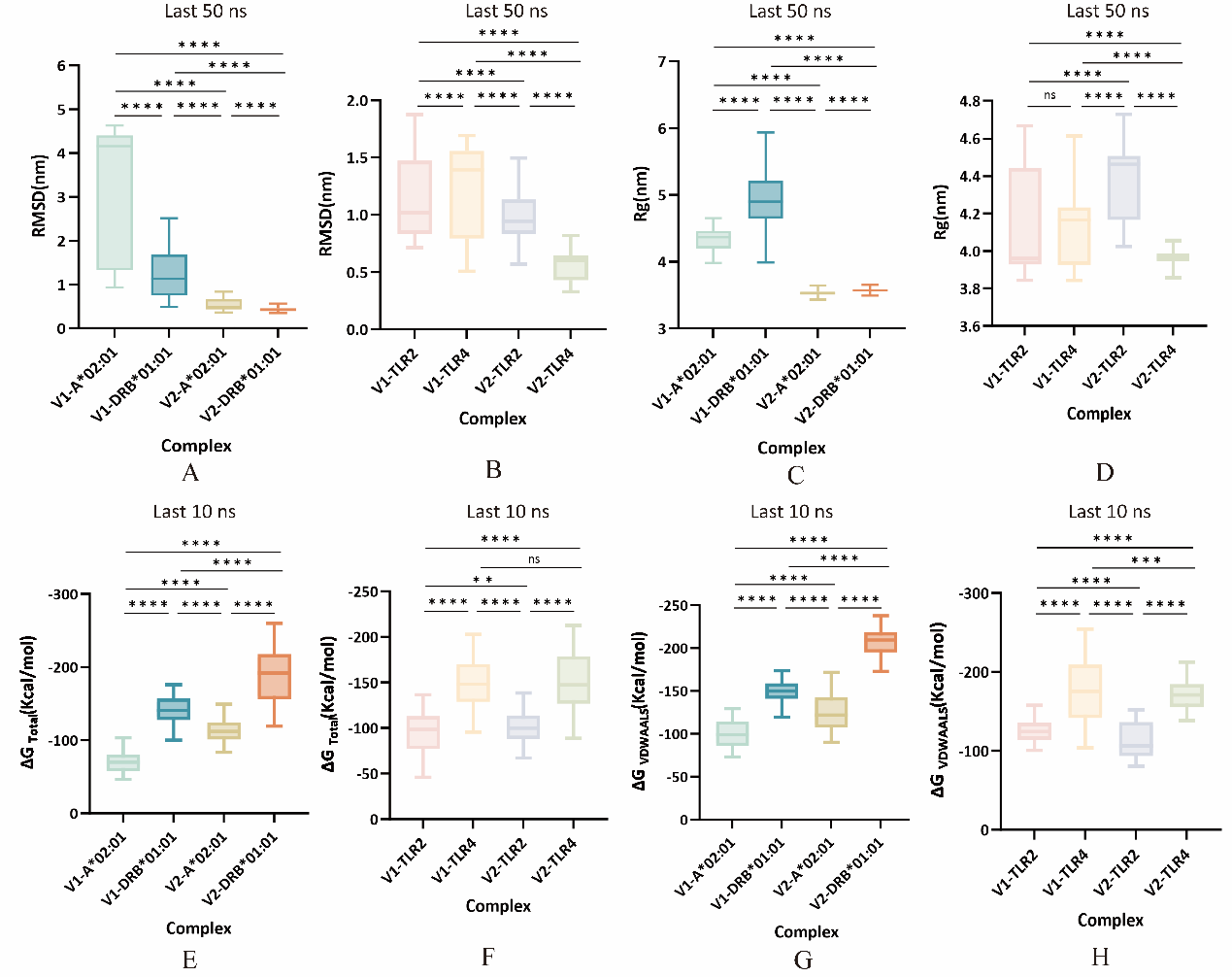


Supplement Figure 23: Structural stability and binding energy analyses of Vaccine 1 and Vaccine 2 in complex with HLA and TLR receptors.

(A–B): RMSD values of the last 50 ns of MD simulations for Vaccine 1 and Vaccine 2 in complexes with HLA-A02:01, HLA-DRB101:01, TLR2, and TLR4. Vaccine 2 consistently exhibited significantly lower RMSD values, indicating enhanced structural stability.

(C–D): Radius of gyration (Rg) of each vaccine–receptor complex, showing that Vaccine 2 maintains a more compact conformation compared with Vaccine 1.

(E–F): Total binding free energy (ΔG_total) calculated by MM/GBSA, demonstrating more favorable binding for Vaccine 2 across all complexes.

(G–H): van der Waals energy (ΔG_vdw) contributions, with Vaccine 2 showing stronger vdw interactions in most complexes.

Statistical differences were determined using one-way ANOVA (ns, not significant; p < 0.05; p < 0.01; p < 0.001; p < 0.0001). These analyses collectively demonstrate superior stability and binding affinity of Vaccine 2 compared with Vaccine 1.

### 2.2.24 Supplement Figure 24


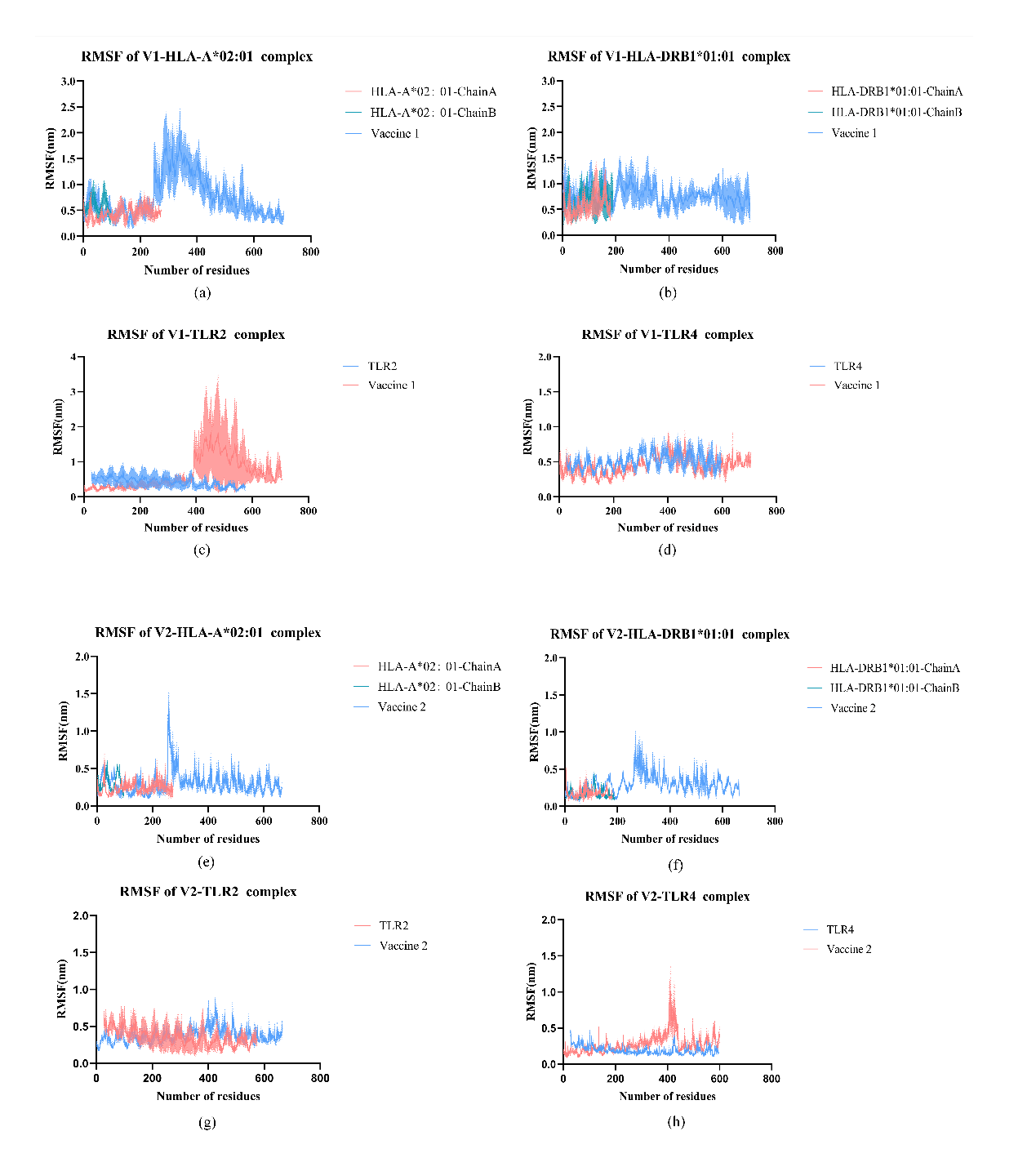


Supplement Figure 24

(A): The RMSF of vaccine 1-HLA-A*02:01 complex; (B): The RMSF of vaccine 1-HLA-DRB1*01:01 complex; (C): The RMSF of vaccine 1-TLR2 complex; (D): The RMSF of vaccine 1-TLR4 complex; (E): The RMSF of vaccine 2-HLA-A*02:01 complex; (F): The RMSF of vaccine 2-HLA-DRB1*01:01 complex; (G): The RMSF of vaccine 2-TLR2 complex; (H): The RMSF of vaccine 2-TLR4 complex.

### 2.2.25 Supplement Figure 25


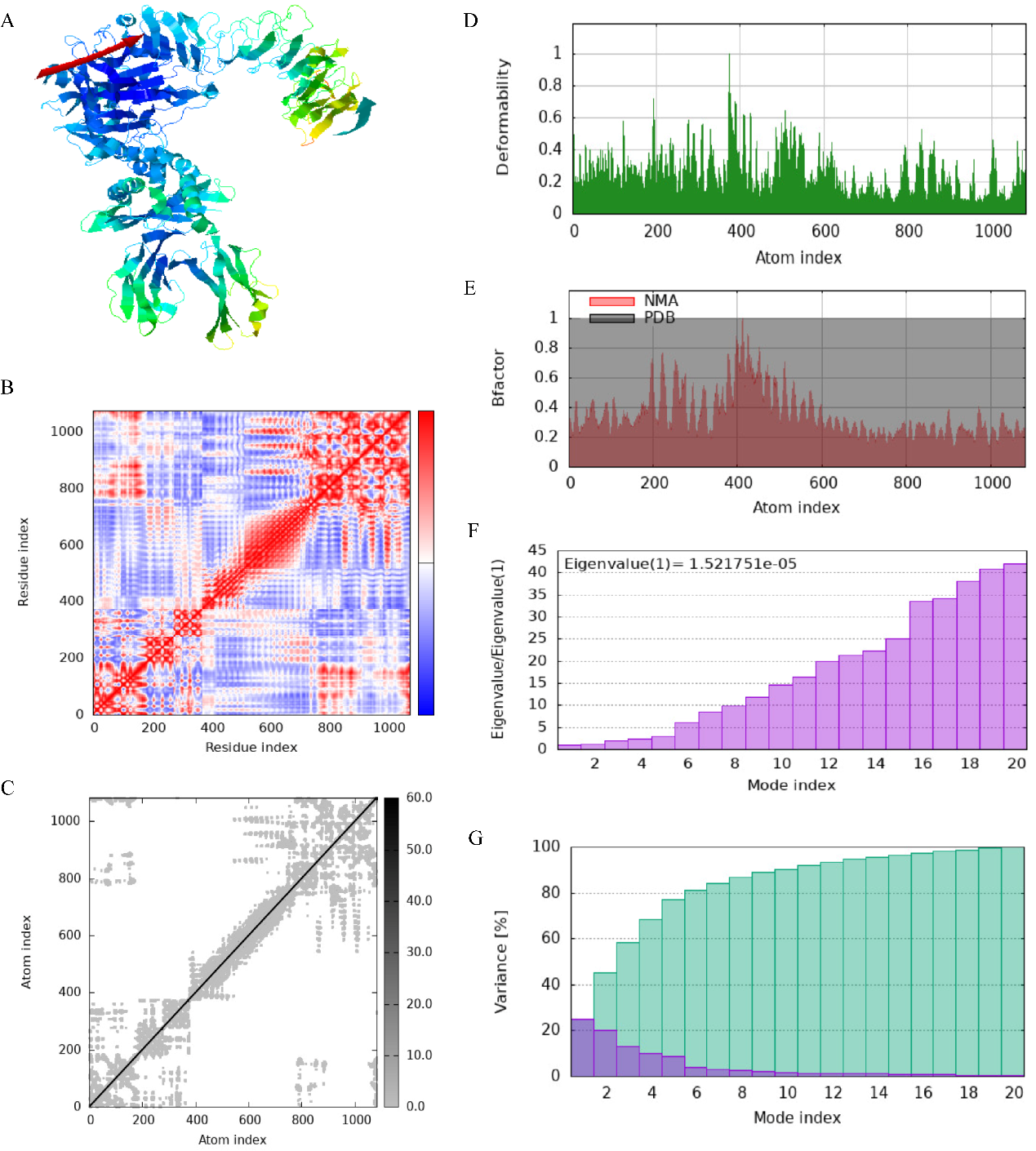


Supplement Figure 25: normal mode analysis and receptor-ligand interactions of vaccine 1-HLA-A*02:01

A: The normal model using the iMODS server; B: covariance matrix; C: elastic network model using the iMODS server; D: deformability graph; E: B-factor graph; F: eigenvalue of the vaccine 1-HLA-A*02:01 complex; G: The variance associated with each normal mode.

### 2.2.26 Supplement Figure 26


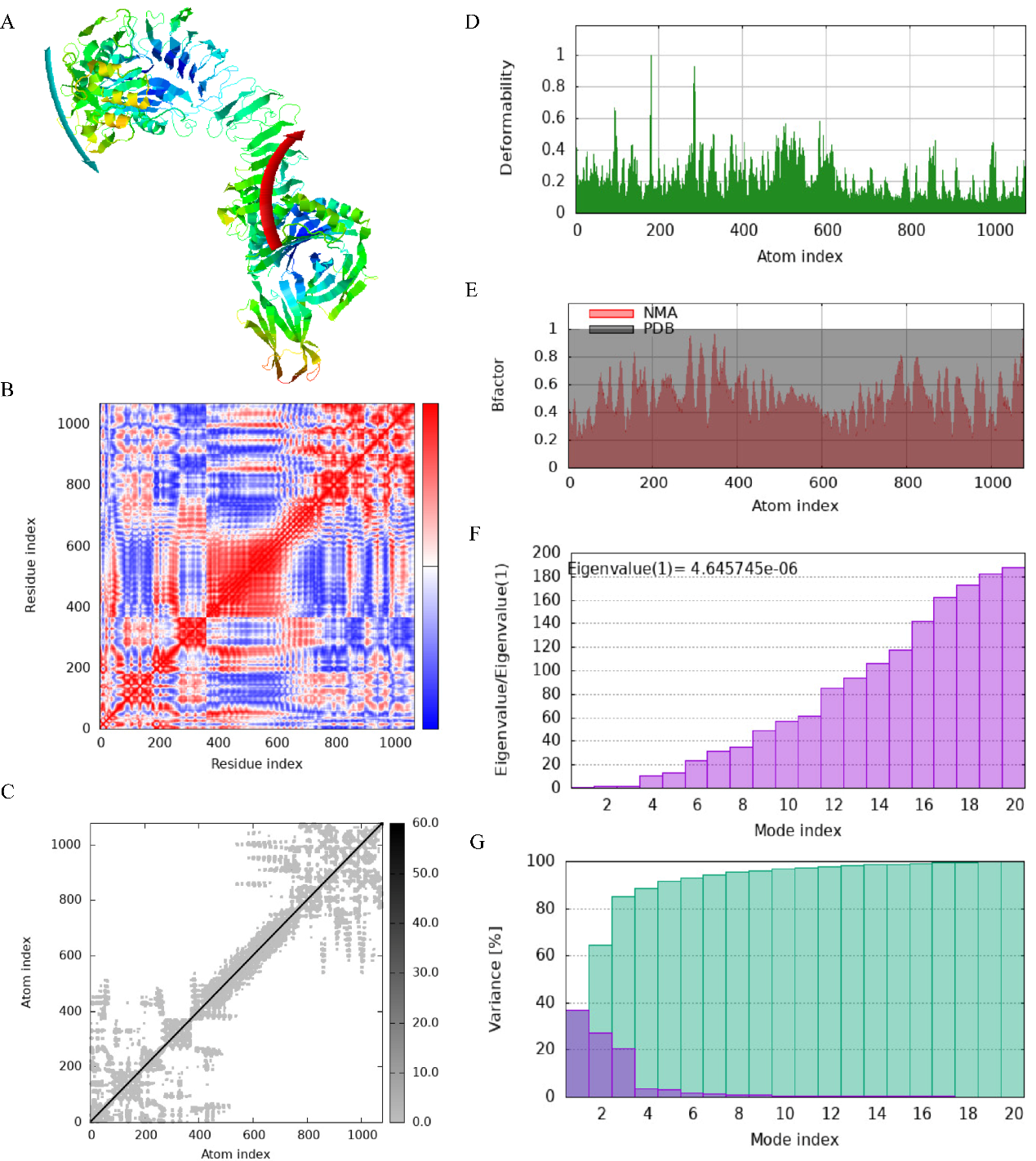


Supplement Figure 26: normal mode analysis and receptor-ligand interactions of vaccine 1-HLA-DRB1*01:01

A: The normal model using the iMODS server; B: covariance matrix; C: elastic network model using the iMODS server; D: deformability graph; E: B-factor graph; F: eigenvalue of the vaccine 1-HLA-DRB1*01:01 complex; G: The variance associated with each normal mode.

### 2.2.27 Supplement Figure 27


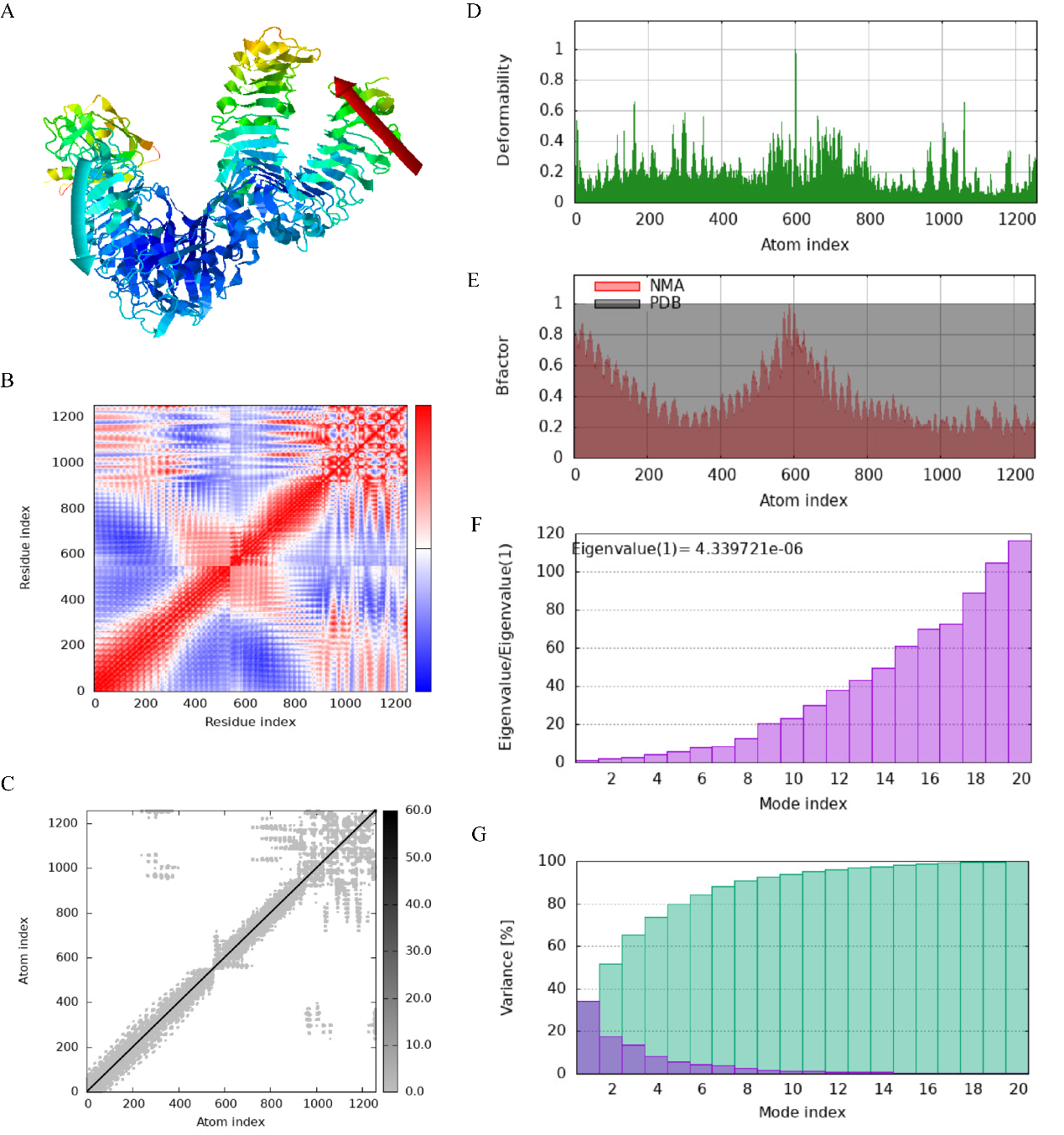


Supplement Figure 27: normal mode analysis and receptor-ligand interactions of vaccine 1-TLR2

A: The normal model using the iMODS server; B: covariance matrix; C: elastic network model using the iMODS server; D: deformability graph; E: B-factor graph; F: eigenvalue of the vaccine 1-TLR2 complex; G: The variance associated with each normal mode.

### 2.2.28 Supplement Figure 28


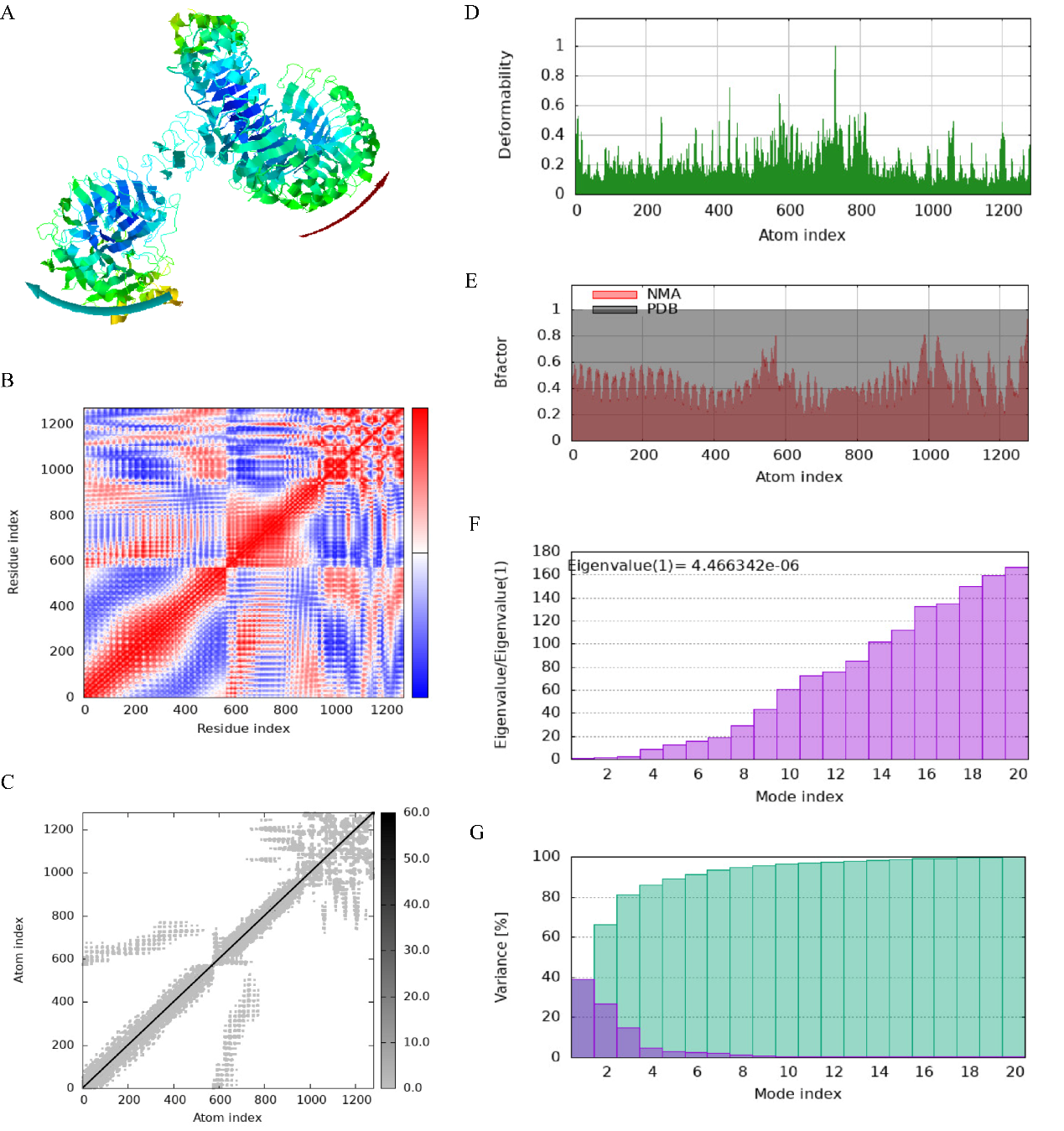


Supplement Figure 28: normal mode analysis and receptor-ligand interactions of vaccine 1-TLR4

A: The normal model using the iMODS server; B: covariance matrix; C: elastic network model using the iMODS server; D: deformability graph; E: B-factor graph; F: eigenvalue of the vaccine 1-TLR4 complex; G: The variance associated with each normal mode.

### 2.2.29 Supplement Figure 29


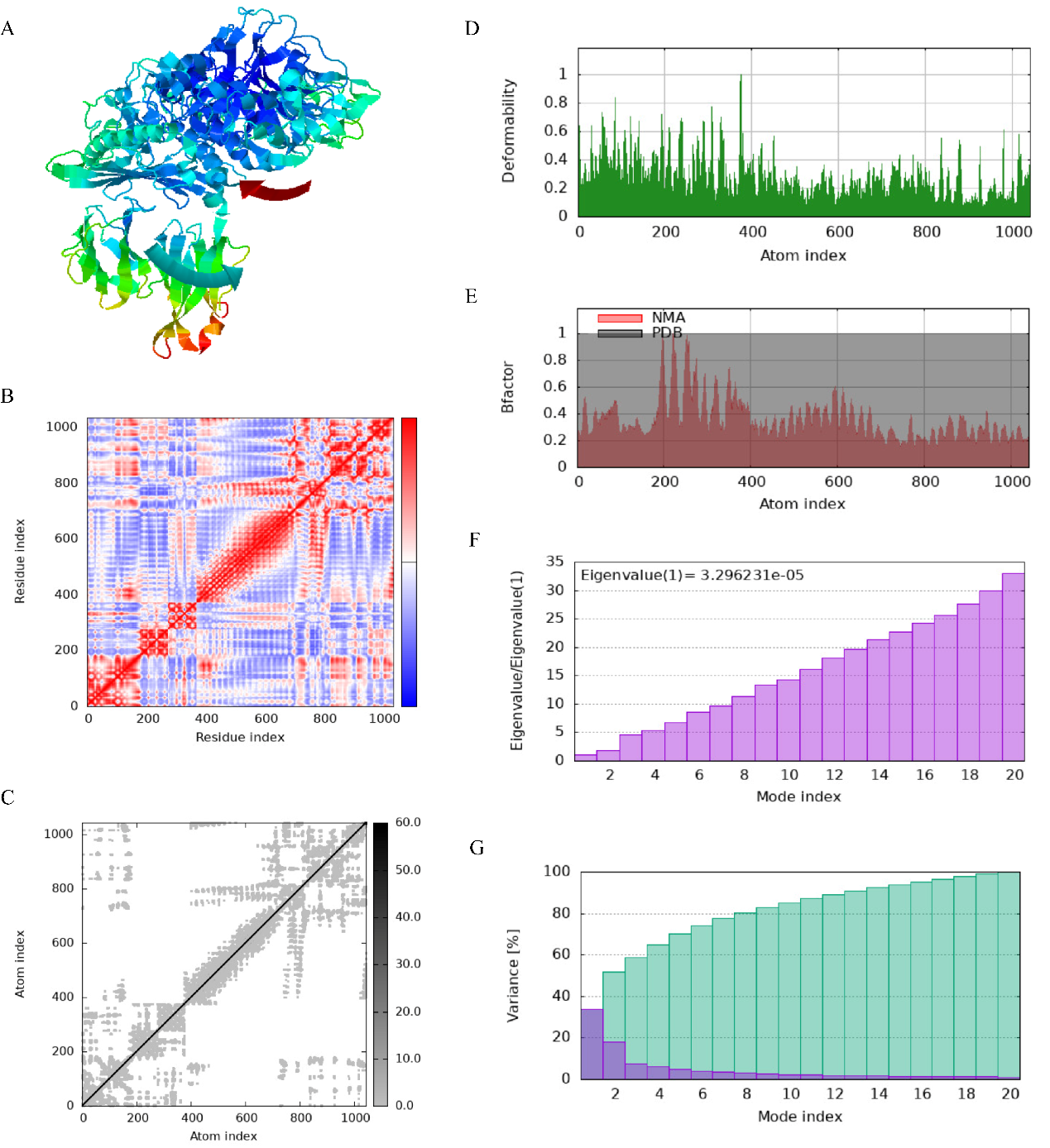


Supplement Figure 29: normal mode analysis and receptor-ligand interactions of vaccine 2-HLA-A*02:01

A: The normal model using the iMODS sever; B: covariance matrix; C: elastic network model using the iMODS server; D: deformability graph; E: B-factor graph; F: eigenvalue of the vaccine 2- HLA-A*02:01 complex; G: The variance associated with each normal mode.

### 2.2.30 Supplement Figure 30


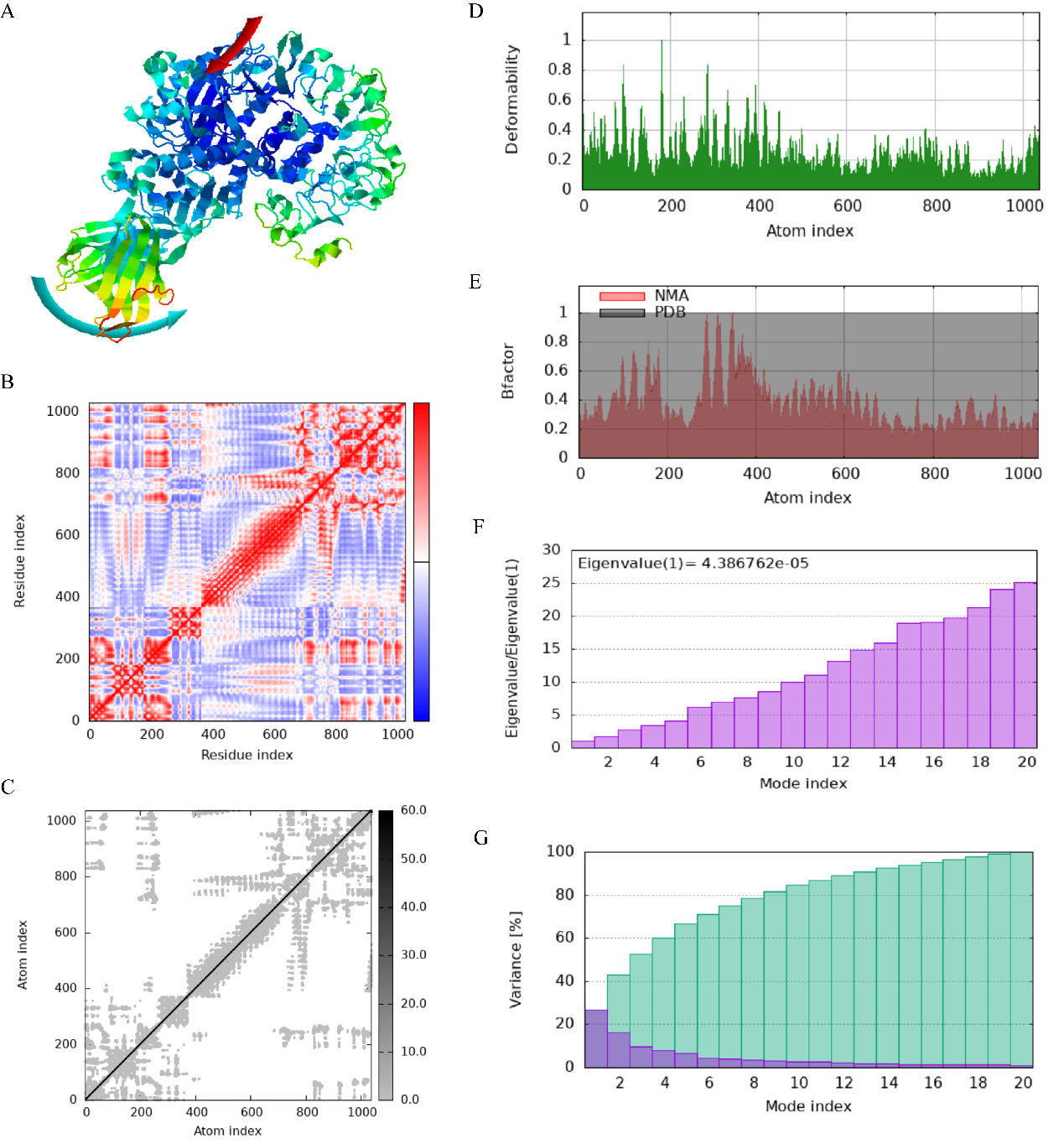


Supplement Figure 30: normal mode analysis and receptor-ligand interactions of vaccine 2-HLA-DRB1*01:01

A: The normal model using the iMODS server; B: covariance matrix; C: elastic network model using the iMODS server; D: deformability graph; E: B-factor graph; F: eigenvalue of the vaccine 2- HLA-DRB1*01:01 complex; G: The variance associated with each normal mode.

### 2.2.31 Supplement Figure 31


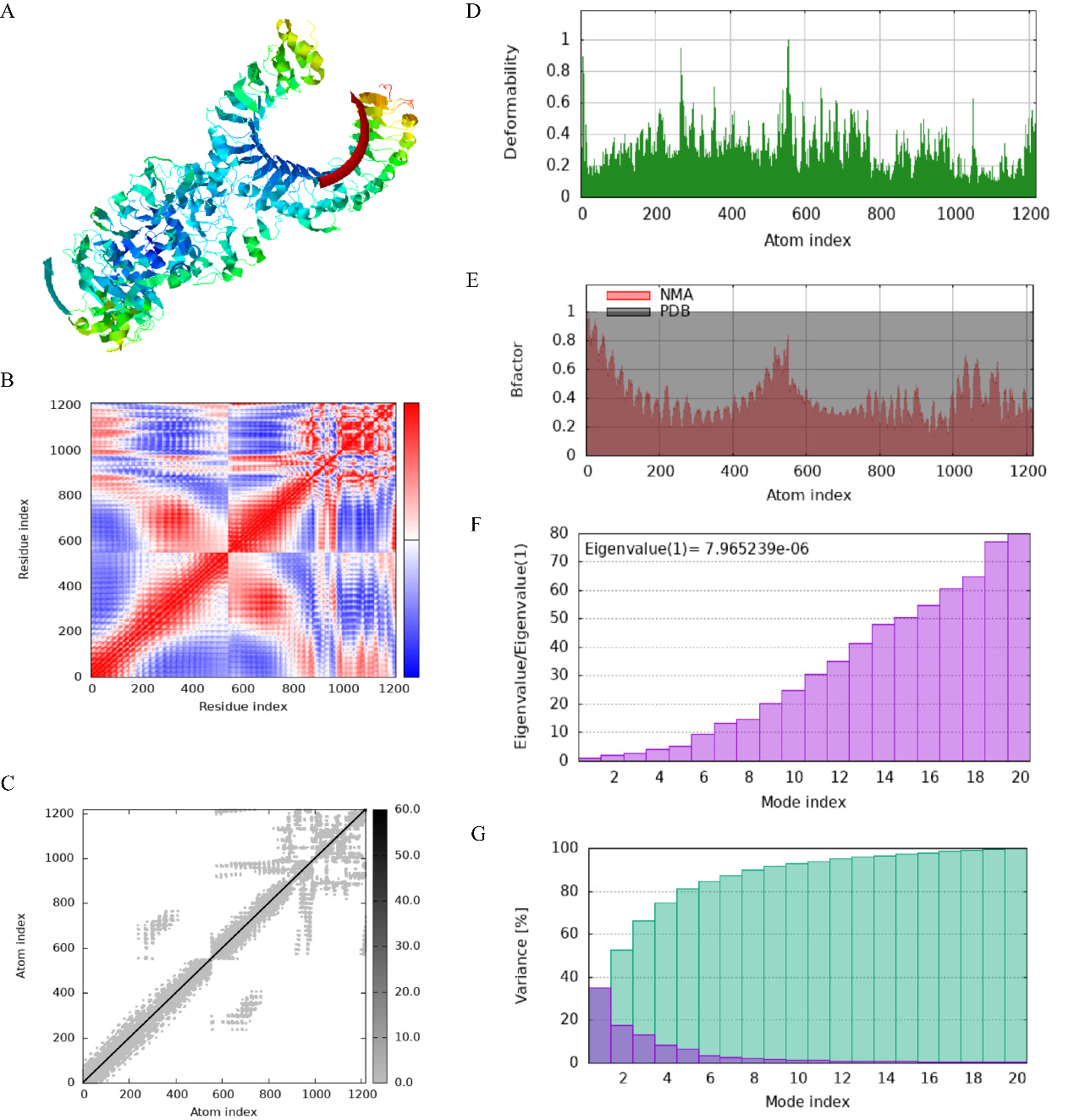


Supplement Figure 31: normal mode analysis and receptor-ligand interactions of vaccine 2-TLR2

A: The normal model using the iMODS server; B: covariance matrix; C: elastic network model using the iMODS server; D: deformability graph; E: B-factor graph; F: eigenvalue of the vaccine 2-TLR2 complex; G: The variance associated with each normal mode.

### 2.2.32 Supplement Figure 32


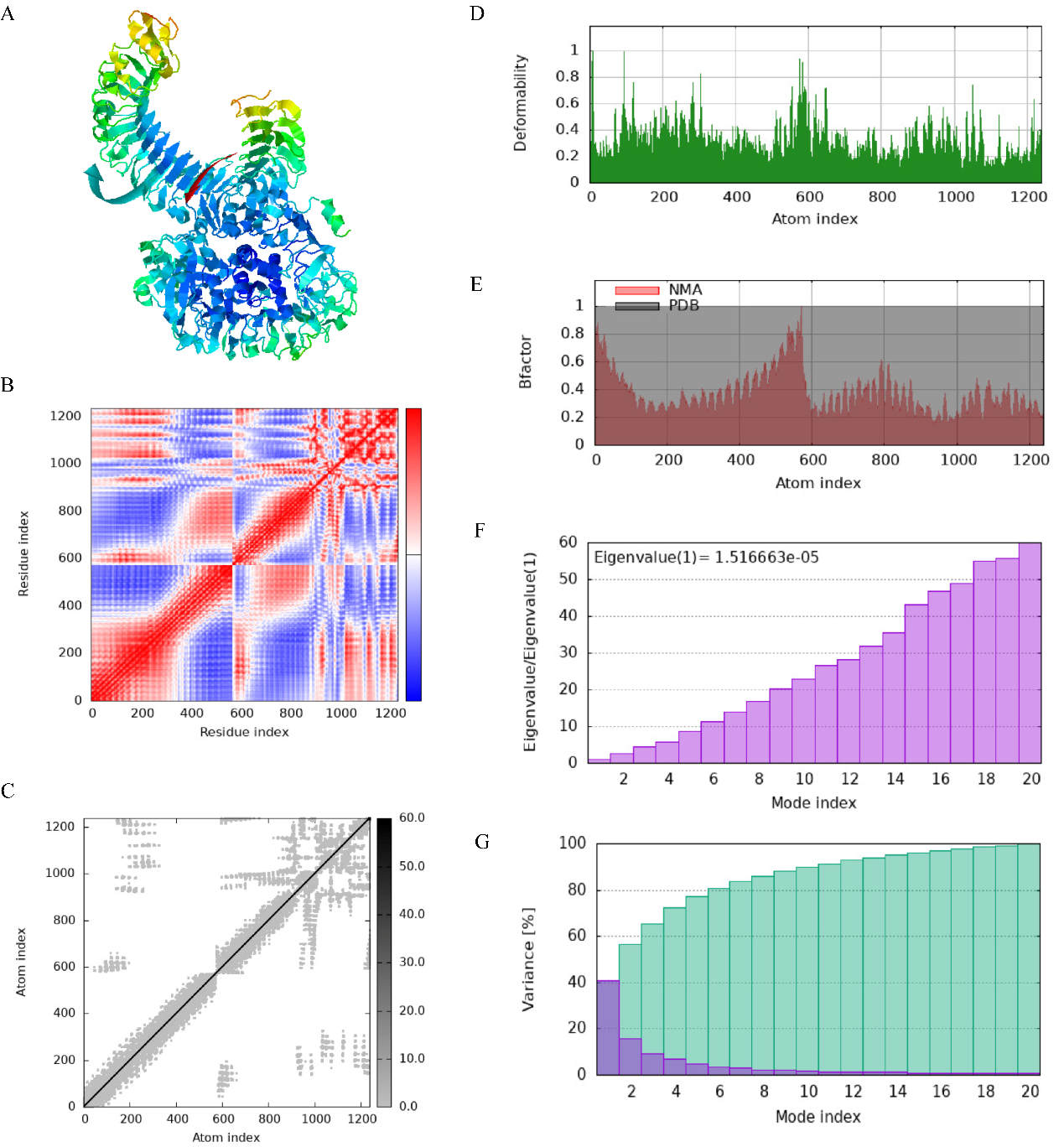


Supplement Figure 32: normal mode analysis and receptor-ligand interactions of vaccine 2-TLR4

A: The normal model using the iMODS server; B: covariance matrix; C: elastic network model using the iMODS server; D: deformability graph; E: B-factor graph; F: eigenvalue of the vaccine 2-TLR4 complex; G: The variance associated with each normal mode.

### 2.2.33 Supplement Figure 33


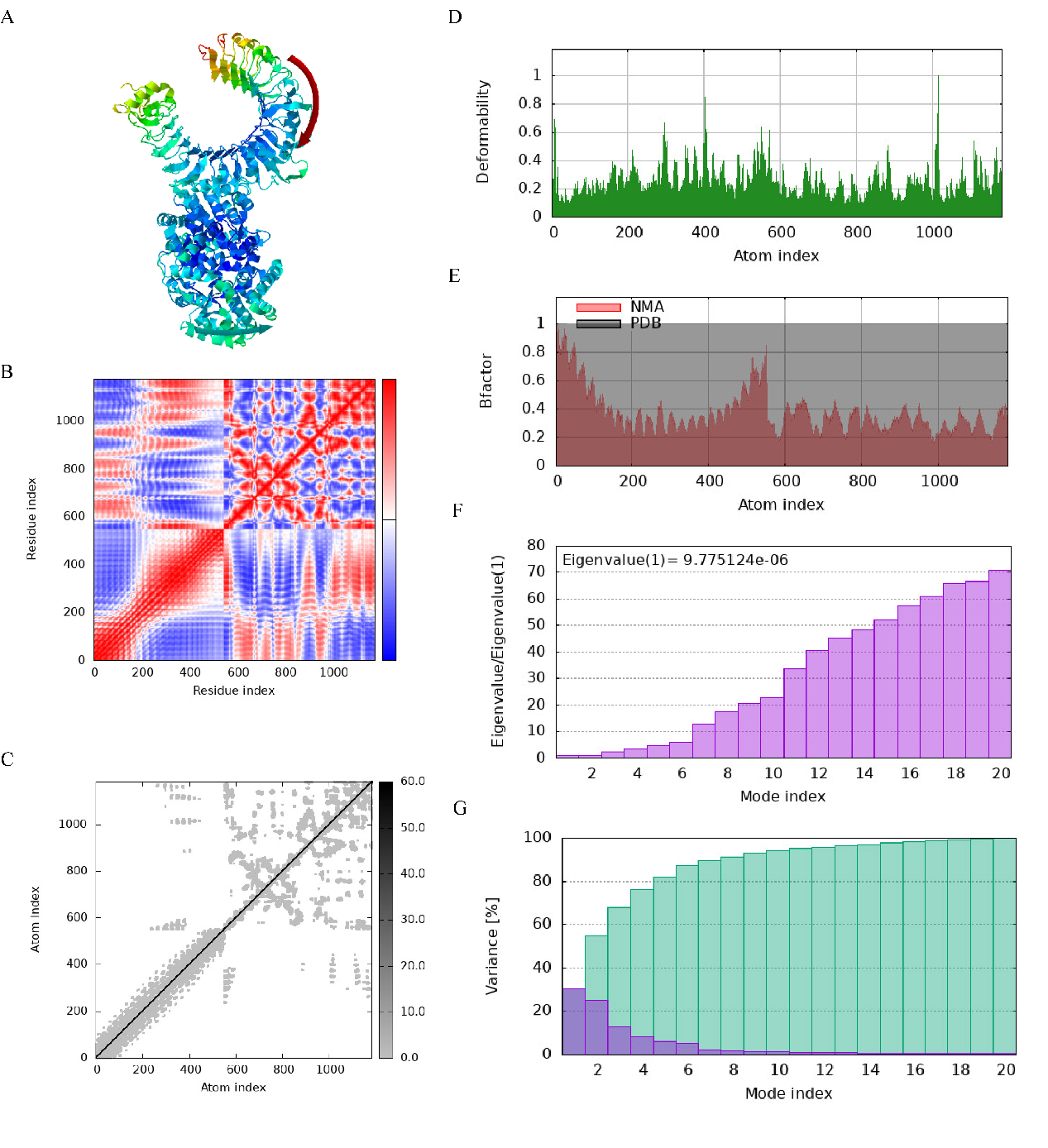


Supplement Figure 33: normal mode analysis and receptor-ligand interactions of PepO-TLR2

A: The normal model using the iMODS server; B: covariance matrix; C: elastic network model using the iMODS server; D: deformability graph; E: B-factor graph; F: eigenvalue of the PepO-TLR2 complex; G: The variance associated with each normal mode.

### 2.2.34 Supplement Figure 34


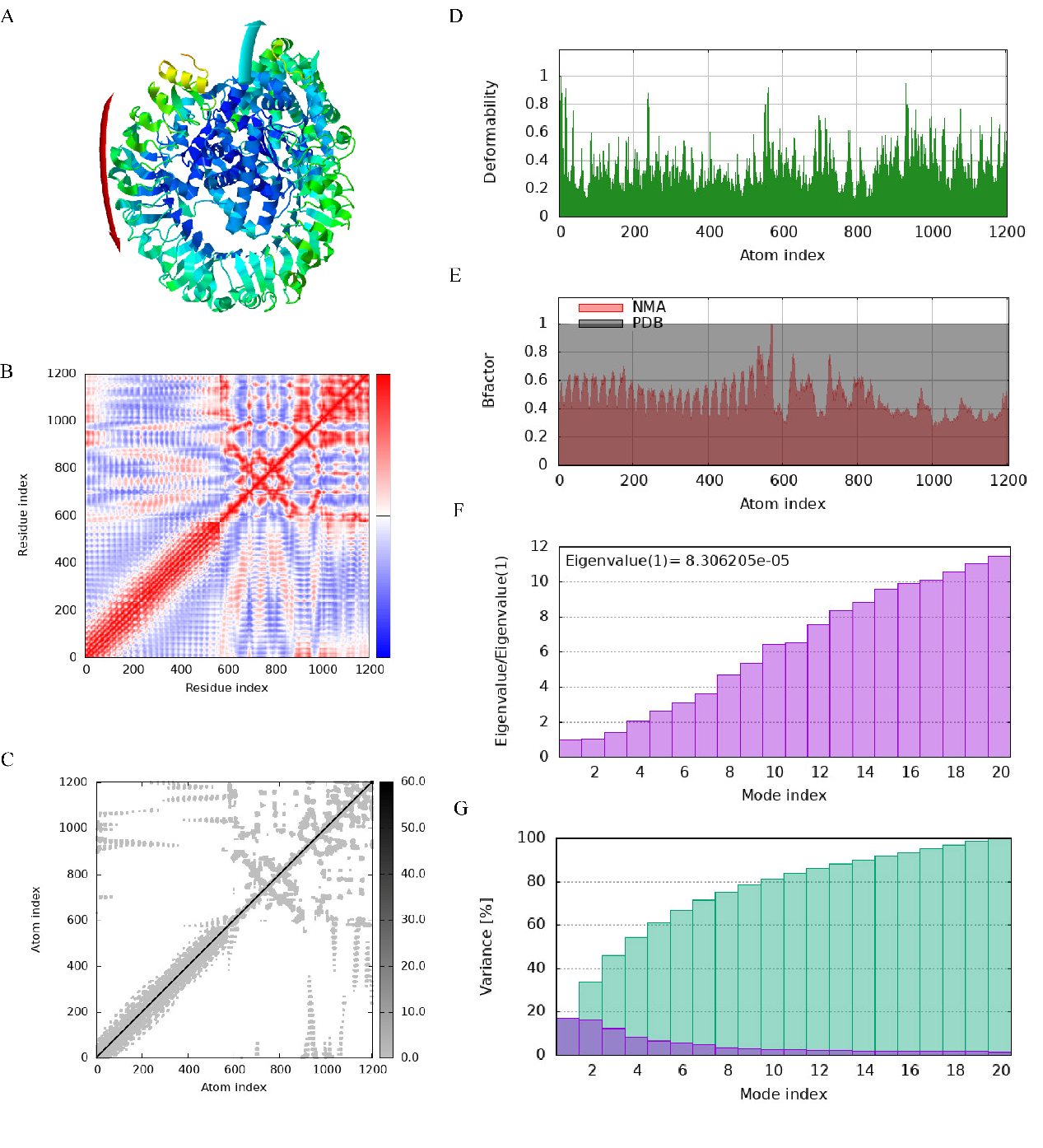


Supplement Figure 34: normal mode analysis and receptor-ligand interactions of PepO-TLR4

A: The normal model using the iMODS server; B: covariance matrix; C: elastic network model using the iMODS server; D: deformability graph; E: B-factor graph; F: eigenvalue of the PepO-TLR4 complex; G: The variance associated with each normal mode.

### 2.2.35 Supplement Figure 35


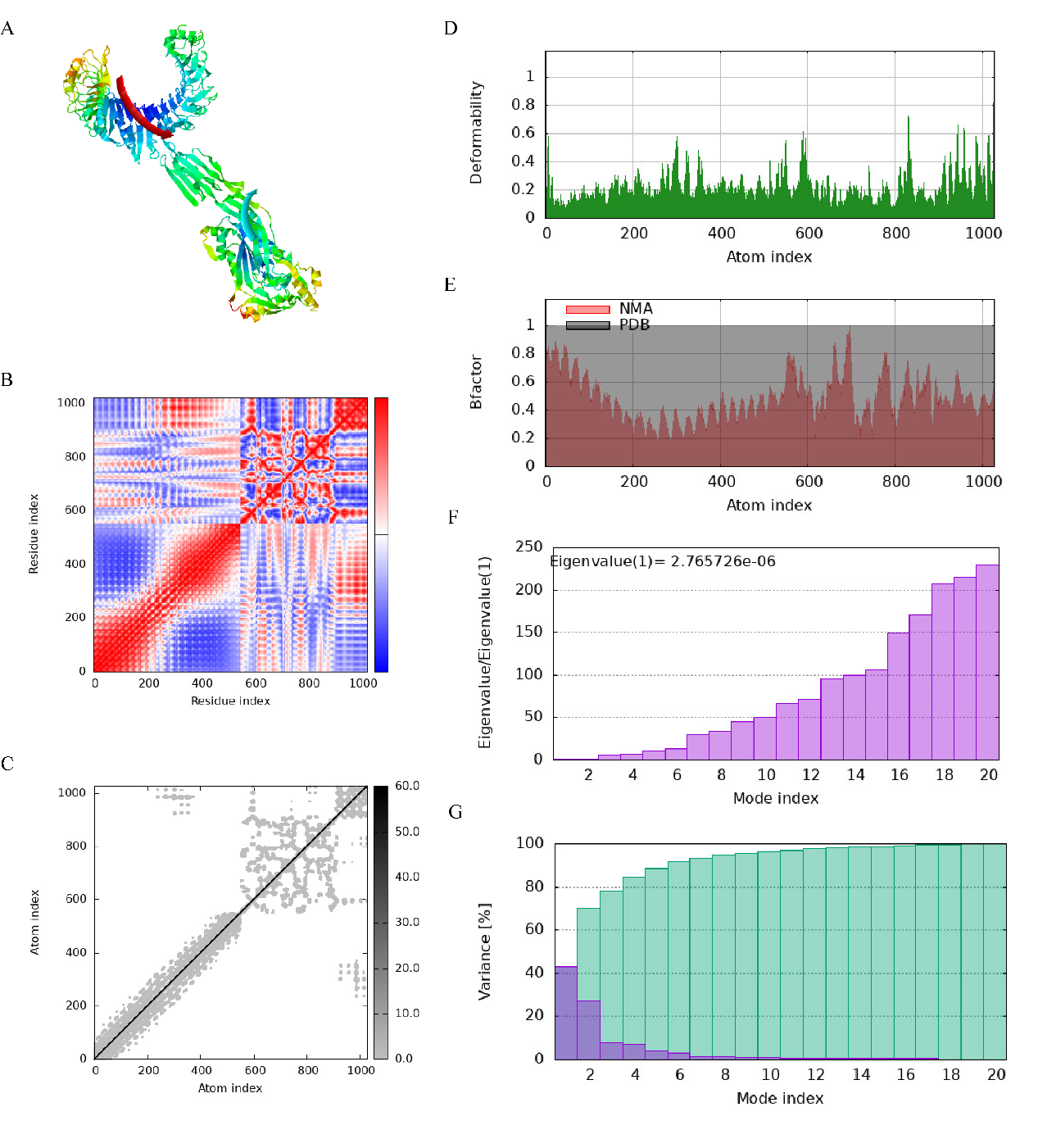


Supplement Figure 35: normal mode analysis and receptor-ligand interactions of Hemolysin-TLR2

A: The normal model using the iMODS server; B: covariance matrix; C: elastic network model using the iMODS server; D: deformability graph; E: B-factor graph; F: eigenvalue of the Hemolysin-TLR2 complex; G: The variance associated with each normal mode.

### 2.2.36 Supplement Figure 36


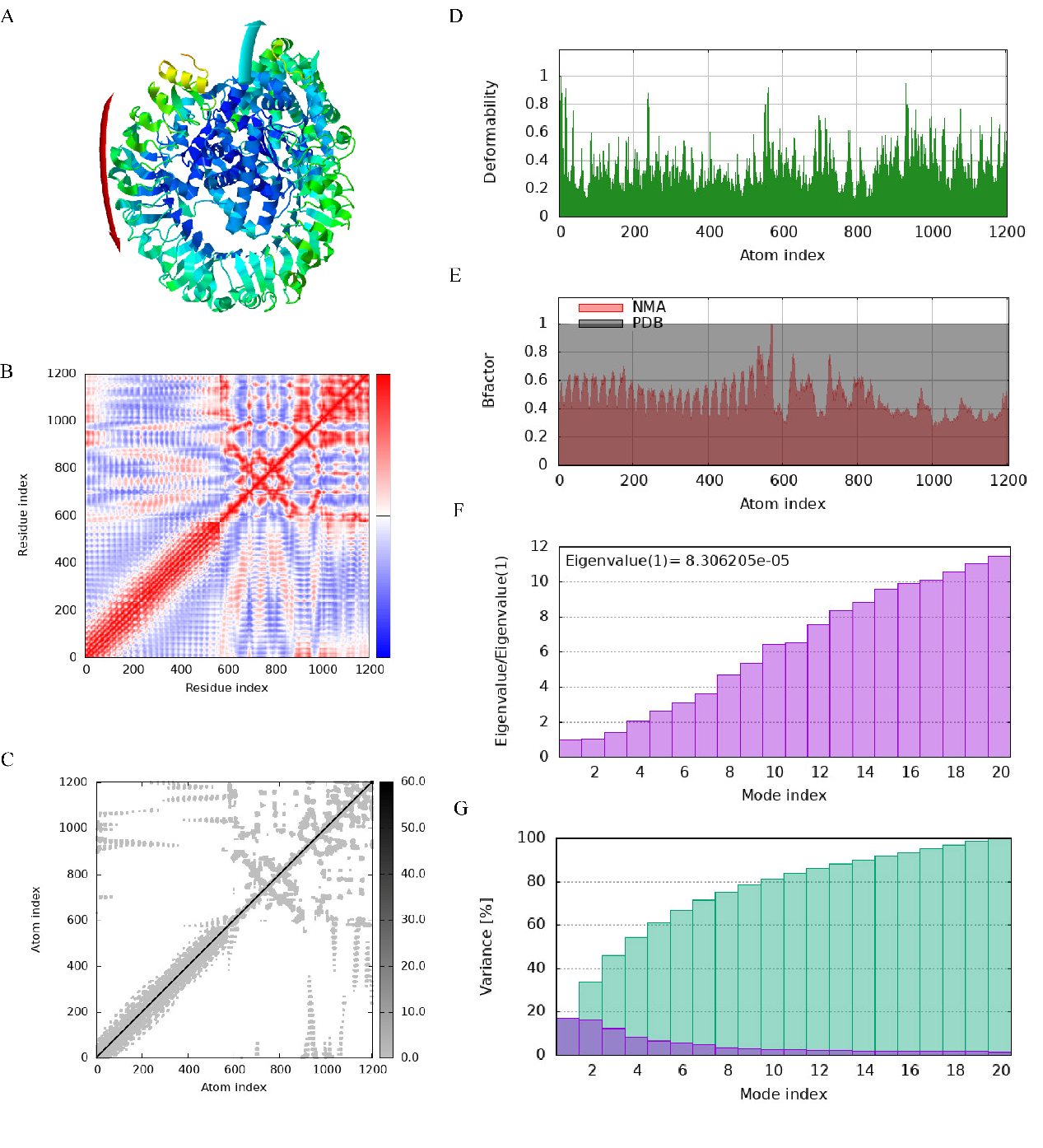


Supplement Figure 36: normal mode analysis and receptor-ligand interactions of Hemolysin-TLR4

A: The normal model using the iMODS server; B: covariance matrix; C: elastic network model using the iMODS server; D: deformability graph; E: B-factor graph; F: eigenvalue of the Hemolysin-TLR4 complex; G: The variance associated with each normal mode.

### 2.2.37 Supplement Figure 37


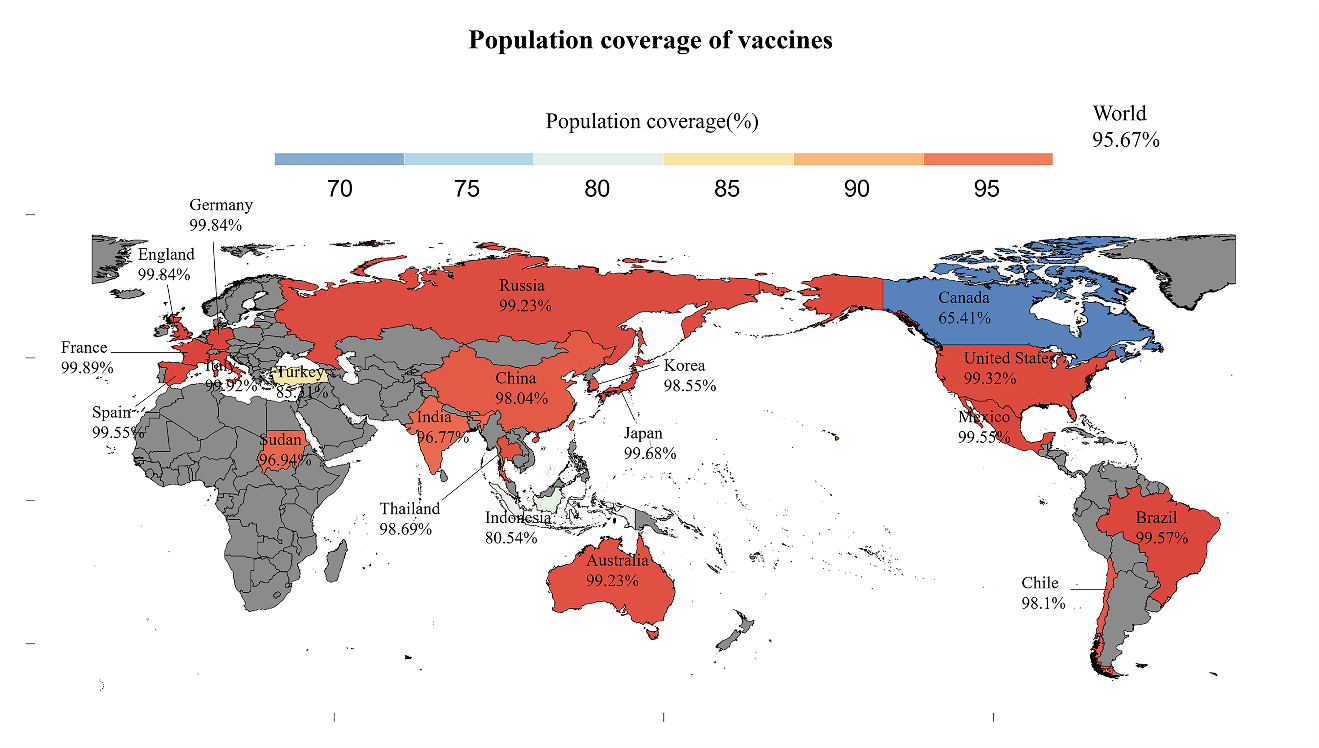


Supplement Figure 37: Population coverage of the vaccines

### 2.2.38 Supplement Figure 38


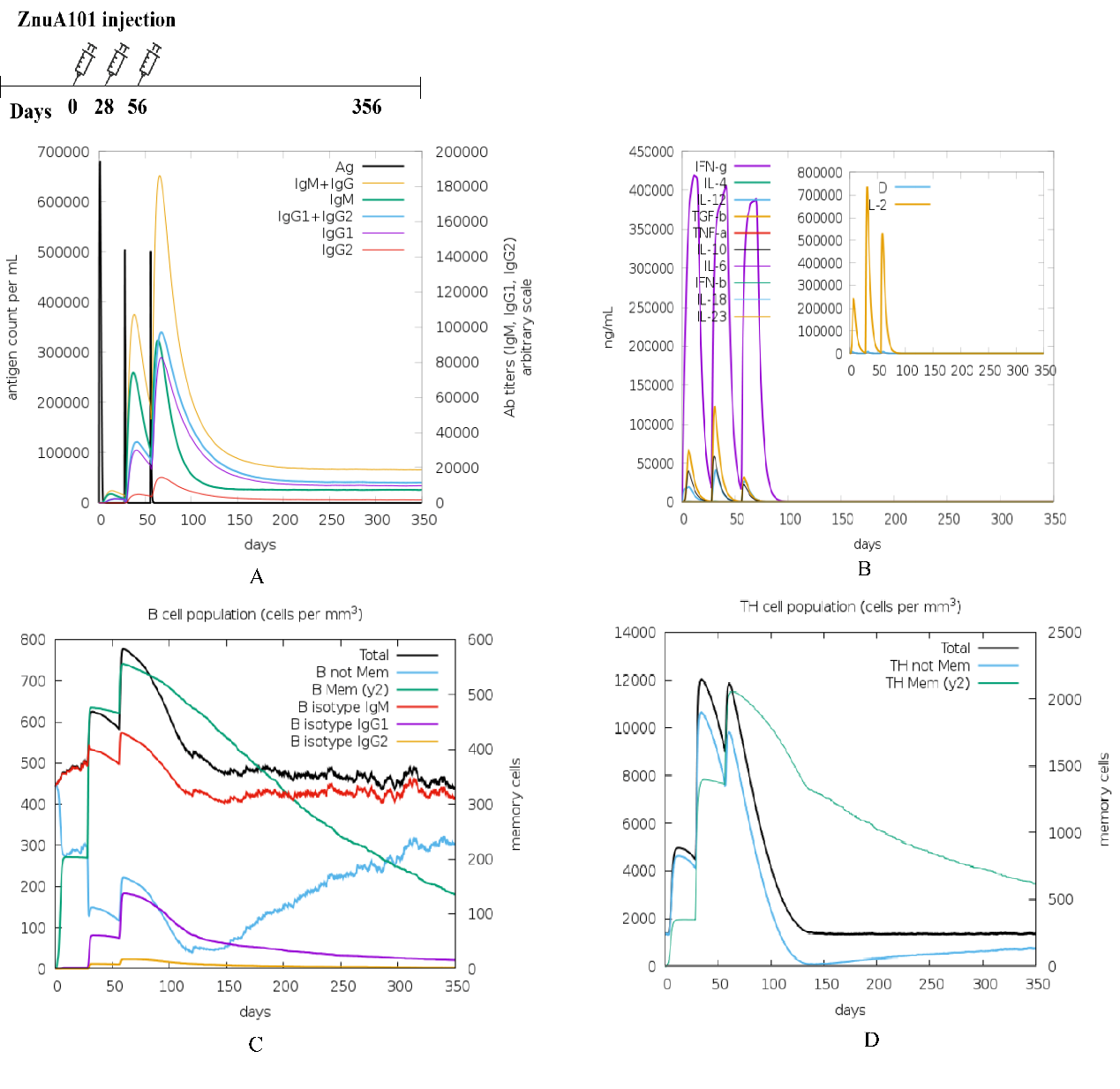


Supplement Figure 38

(A) Antibody levels induced by three doses of *S. aureus* epitope vaccine injections; (B) Levels of cytokines induced by *S. aureus* epitope vaccine; (C) Population of B cells induced by *S. aureus* epitope vaccine; (D) Population of helper T(TH) cells with *S. aureus* epitope vaccine.
